# Drug Delivery Systems for Breast Cancer Treatment: From Design Principles to Clinical Translation

**DOI:** 10.3390/cancers18203393

**Published:** 2026-10-20

**Authors:** Vasile Adrian Sescu, Iustina Petra Solomon Condriuc, Liana Gabriela Spridon, Anca Niculina Cadinoiu, Lucian Miron, Leonard Ionut Atanase, Roxana Daniela Matasariu, Demetra Gabriela Socolov, Alexandru Carauleanu

**Affiliations:** 1 Department of Obstetrics and Gynecology, Faculty of General Medecine, Grigore T. Popa University of Medicine and Pharmacy, 700115 Iasi, Romania; vasile-adrian.sescu@d.umfiasi.ro (V.A.S.); daniela.matasariu@umfiasi.ro (R.D.M.); demetra.socolov@umfiasi.ro (D.G.S.); ale.carauleanu@umfiasi.ro (A.C.); 2 Cristofor Simionescu Faculty of Chemical Engineering and Environmental Protection, Gheorghe Asachi Technical University of Iasi, 700050 Iasi, Romania; liana-gabriela.spridon@student.tuiasi.ro; 3 Faculty of Medicine, Apollonia University of Iasi, 700055 Iasi, Romania; anca.n.cadinoiu@univapollonia.ro (A.N.C.); lucmir@gmail.com (L.M.); 4 Academy of Romanian Scientists, 050044 Bucharest, Romania

**Keywords:** breast cancer, drug delivery systems, nanomedicine, nanocarriers, targeted drug delivery, clinical translation

## Abstract

Background/Objectives: Breast cancer is the most frequently diagnosed malignancy worldwide and remains a major cause of cancer-related mortality among women. Despite advances in screening, molecular classification, and systemic therapies, treatment of advanced and metastatic disease remains challenging because of tumor heterogeneity, multidrug resistance, systemic toxicity, limited drug selectivity, and inadequate penetration into the tumor microenvironment. This review aims to critically evaluate advanced drug delivery systems (DDSs) for breast cancer therapy, emphasizing the relationship between carrier design, tumor biology, therapeutic performance, and clinical translation. Methods: The recent literature on DDSs for breast cancer treatment was comprehensively reviewed, covering liposomes, polymeric nanoparticles and micelles, dendrimers, nanogels, lipid- and inorganic-based nanoparticles, extracellular vesicles, biomimetic platforms, and stimuli-responsive systems. Particular attention was given to active and passive targeting, controlled drug release, strategies for overcoming multidrug resistance and biological barriers, and combination approaches integrating chemotherapy with immunotherapy, gene therapy, and phototherapy. Clinically approved and clinical-stage nanomedicines were also considered. Results: Advanced DDSs can improve drug stability, pharmacokinetics, biodistribution, tumor accumulation, cellular uptake, and controlled release, potentially enhancing therapeutic efficacy while reducing systemic toxicity. Targeted, biomimetic, and stimuli-responsive platforms provide additional opportunities to address tumor heterogeneity, the tumor microenvironment, and therapeutic resistance. Several DDS-based nanomedicines have reached clinical use or advanced clinical development, demonstrating their translational potential. However, manufacturing scalability, reproducibility, long-term safety, regulatory requirements, and patient heterogeneity remain important barriers. Conclusions: Advanced DDSs represent promising tools for improving the precision and efficacy of breast cancer treatment. Future progress will require biologically informed carrier design, reproducible manufacturing, rigorous clinical validation, and patient stratification. Integrating nanomedicine with precision oncology may accelerate the clinical translation of next-generation DDSs and support more effective personalized therapeutic strategies.

## Simple Summary

Breast cancer is the most commonly diagnosed cancer worldwide and remains a major cause of cancer-related deaths among women. Although important advances have been made in diagnosis and treatment, many patients with advanced or metastatic breast cancer still face treatment resistance and significant side effects. This review examines new drug delivery systems designed to help anticancer medicines reach tumors more effectively while reducing damage to healthy tissues. These systems include tiny carriers such as liposomes, polymer-based nanoparticles, micelles, nanogels, and other innovative platforms. They can protect drugs, improve their stability, control how they are released, and help deliver them more specifically to cancer cells. Some approaches also combine drug delivery with immunotherapy, gene therapy, or light-based treatments. Several such systems have already reached clinical use or testing. However, challenges related to safety, manufacturing, reproducibility, and patient differences remain. Continued advances could support more precise and personalized breast cancer treatment.

## 1. Introduction

Breast cancer remains one of the major global health challenges, representing the most frequently diagnosed malignancy worldwide and one of the leading causes of cancer-related mortality among women. According to the most recent GLOBOCAN-based estimates, approximately 2.3 million new cases of female breast cancer and 670,000 deaths occurred globally in 2022 [1]. Although substantial improvements in screening, early diagnosis, and treatment have contributed to declining mortality in several high-income countries, pronounced geographical disparities persist. In particular, countries with low and medium human development indices continue to experience higher mortality rates and more limited access to timely diagnosis and effective treatment [1,2]. These disparities are expected to become increasingly important as the global burden of breast cancer continues to rise. If current incidence patterns persist, the annual number of new cases is projected to increase by approximately 38% by 2050, reaching approximately 3.2 million cases, while annual deaths could increase by approximately 68%, reaching approximately 1.1 million deaths [1]. The disproportionate increase expected in low-human-development countries highlights the urgent need not only for improved prevention and early detection but also for affordable, effective, and scalable therapeutic strategies [1,2,3].

The epidemiological landscape of breast cancer is continuously evolving. Asia accounts for the largest absolute number of newly diagnosed cases, primarily reflecting its large population, whereas countries in Africa and other low-resource settings experience substantially higher mortality relative to incidence, reflecting inequalities in cancer awareness, screening, diagnostic capacity, treatment availability, and access to modern systemic therapies [1,2,3]. Age-related patterns are also changing, with several countries reporting increasing breast cancer incidence among women younger than 50 years [1,4]. Despite excellent outcomes for patients diagnosed with localized disease, for whom 5-year relative survival exceeds 99%, survival decreases substantially with regional and particularly distant disease, emphasizing the continued need for more effective strategies for preventing recurrence and treating advanced breast cancer [5,6].

Breast cancer is not a single disease but a highly heterogeneous group of malignancies characterized by distinct molecular profiles, biological behaviors, prognoses, and therapeutic responses [7,8]. The major intrinsic molecular subtypes include luminal A, luminal B, human epidermal growth factor receptor 2 (HER2)-enriched, and basal-like breast cancers, with triple-negative breast cancer (TNBC) representing an important clinically defined subgroup lacking expression of estrogen receptor (ER), progesterone receptor (PR), and HER2 [7,8,9]. Molecular classification has substantially improved patient stratification and enabled the development of targeted treatments that have transformed the management of hormone receptor-positive and HER2-positive disease. Nevertheless, TNBC remains particularly challenging because of its aggressive phenotype, increased metastatic potential, limited availability of validated molecular targets, and relatively high risk of recurrence, particularly during the first few years following diagnosis [9,10]. Furthermore, intratumoral and intertumoral heterogeneity, dynamic clonal evolution, phenotypic plasticity, and complex interactions between malignant cells and the tumor microenvironment contribute to disease progression and therapeutic resistance, complicating treatment selection and limiting durable responses [11,12].

Current systemic treatment strategies include cytotoxic chemotherapy, endocrine therapy, anti-HER2 targeted therapy, immune checkpoint inhibition, and, increasingly, antibody-drug conjugates. These therapeutic advances have significantly improved outcomes for multiple breast cancer subtypes; however, important limitations remain, particularly in advanced and metastatic disease [8,13]. One of the principal challenges is the development of intrinsic or acquired therapeutic resistance. In hormone receptor-positive breast cancer, endocrine resistance can arise through multiple mechanisms, including alterations in ER signaling, activation of compensatory growth pathways, changes in hormone receptor expression, and clonal evolution under therapeutic pressure [14,15]. Resistance to chemotherapy may similarly arise through diverse mechanisms involving altered drug transport and metabolism, enhanced DNA repair, evasion of apoptosis, cancer stem-cell populations, epithelial–mesenchymal plasticity, and adaptive changes within the tumor microenvironment [16,17]. Even molecularly targeted therapies can ultimately lose efficacy because of target alteration, pathway reactivation, bypass signaling, or clonal selection, underscoring the need for therapeutic strategies capable of addressing the multifactorial nature of resistance [11,18].

Dose-limiting toxicities—including anthracycline-associated cardiotoxicity, chemotherapy-induced peripheral neuropathy, myelosuppression, gastrointestinal toxicity, alopecia, fatigue, and other treatment-related adverse effects—may compromise treatment intensity, adherence, and quality of life [19,20]. Endocrine therapies, although generally less systemically toxic than cytotoxic chemotherapy, can also produce clinically relevant long-term adverse effects, including vasomotor symptoms, musculoskeletal complications, sexual dysfunction, and treatment-associated bone loss [21]. Moreover, metastatic breast cancer remains generally incurable, and treatment is therefore primarily directed toward prolonging survival, controlling disease progression, alleviating symptoms, and maintaining quality of life rather than achieving definitive eradication of disease [22].

The abnormal architecture and function of tumor-associated vasculature, elevated interstitial fluid pressure, dense extracellular matrix, and heterogeneous tumor perfusion can severely restrict drug penetration and distribution within malignant tissues [23,24]. Although the enhanced permeability and retention (EPR) effect has provided an important conceptual basis for nanoparticle-mediated tumor targeting, its magnitude and therapeutic relevance are highly heterogeneous among tumors and patients. Nanoparticles may therefore accumulate preferentially in some tumor regions while remaining poorly distributed in others, limiting therapeutic efficacy [23,24,25].

These limitations have stimulated extensive research into advanced drug delivery systems (DDSs) designed to improve the therapeutic index of anticancer agents by modifying their pharmacokinetics, biodistribution, stability, release behavior, and cellular uptake [26,27,28]. Nanotechnology-based delivery platforms—including liposomes, polymeric nanoparticles, polymeric micelles, dendrimers, nanogels, lipid nanoparticles, inorganic nanoparticles, extracellular vesicles, and biomimetic systems—provide versatile approaches for the delivery of structurally and mechanistically diverse therapeutic cargos, including small-molecule chemotherapeutics, nucleic acids, proteins, peptides, and immunomodulatory agents [26,27,28,29]. Depending on their physicochemical characteristics and biological interactions, these systems may facilitate prolonged circulation, improve drug solubility and stability, reduce premature degradation, enhance cellular uptake, and enable controlled drug release. Targeting strategies can involve passive accumulation based on tumor-associated vascular and stromal characteristics or active targeting through ligands directed toward receptors overexpressed by tumor cells or components of the tumor microenvironment, including HER2, folate receptor, transferrin receptor, epidermal growth factor receptor (EGFR), and CD44 [27,30].

More recently, stimuli-responsive or “smart” delivery systems have attracted considerable interest because they can be engineered to respond to endogenous characteristics of tumors—including acidic pH, redox gradients, enzymatic activity, hypoxia, and reactive oxygen species—or to exogenous stimuli such as temperature, ultrasound, magnetic fields, and light [31,32]. Such systems aim to achieve temporally and spatially controlled drug release, potentially improving drug exposure at the desired site while reducing systemic toxicity.

Despite impressive preclinical results, however, the clinical translation of nanomedicine remains considerably more challenging than anticipated. The biological complexity of human tumors, interpatient variability in vascular permeability and nanoparticle accumulation, heterogeneous tumor microenvironments, limitations of passive targeting, and uncertainty regarding the predictive value of preclinical models can all contribute to discrepancies between experimental and clinical outcomes [23,25,33]. Additional barriers include large-scale manufacturing, batch-to-batch reproducibility, control of critical quality attributes, long-term safety assessment, regulatory requirements, formulation stability, scalability, and cost-effectiveness [33,34,35]. Consequently, only a limited number of nanomedicine platforms have successfully progressed from laboratory development to routine clinical application, despite the large number of candidates reported in the preclinical literature [33,34]. This translational gap emphasizes that successful nanomedicine development requires consideration not only of carrier composition and physicochemical properties but also of biological barriers, patient-specific tumor characteristics, manufacturing processes, regulatory requirements, and clinically meaningful endpoints.

Against this background, the present review provides a comprehensive and translationally oriented overview of drug delivery systems developed for breast cancer therapy, spanning the continuum from fundamental carrier design principles to clinical implementation. An electronic literature search was conducted from database inception through July 2026 using PubMed (NCBI), Scopus (Elsevier), and Web of Science (Clarivate). In addition, the reference lists of all articles included in the systematic review were manually screened to identify potentially relevant publications that may not have been retrieved through the electronic database search. No authors were contacted to obtain missing or additional information, as all required documents were accessible.

Unlike reviews focusing predominantly on individual nanocarrier platforms or specific therapeutic modalities, this work integrates the physicochemical principles governing carrier engineering with the biological interactions that determine transport, tumor penetration, cellular uptake, intracellular trafficking, and therapeutic response. Particular emphasis is placed on the relationship between nanocarrier composition, architecture, physicochemical properties, and biological performance; strategies for overcoming multidrug resistance and tumor-associated delivery barriers; active and passive targeting approaches; emerging stimuli-responsive and multifunctional systems; and combination strategies integrating chemotherapy with immunotherapy, gene therapy, phototherapy, or other therapeutic modalities. The review further examines clinically approved nanomedicines and selected clinical-stage technologies, together with the manufacturing, regulatory, safety, reproducibility, and patient-selection challenges that influence successful translation. Finally, future perspectives are discussed with emphasis on the development of next-generation precision DDSs capable of improving therapeutic efficacy, overcoming biological resistance, minimizing systemic toxicity, and ultimately enhancing survival and quality of life for patients with breast cancer.

## 2. Biological Barriers and Challenges of Therapeutic Delivery in Breast Cancer

Breast cancer represents one of the oncological pathologies with the most complex biological and molecular profile, and equitable access to diagnosis and treatment remains a major challenge even within advanced healthcare systems [36,37]. From a biological and molecular standpoint, the efficacy of drug delivery systems is conditioned by the multitude of barriers intrinsic to the tumor and its immunosuppressive microenvironment, whose thorough understanding constitutes the indispensable prerequisite of any next-generation therapeutic strategy [38]. Thus, among the ensemble of biological barriers in breast cancer, one can identify aspects ranging from the heterogeneity of the tumor microenvironment to cellular and subcellular obstacles alongside complex resistance mechanisms. These collectively constitute the biological expression of an active and adaptive tumor ecosystem, whose systematic and rational circumvention through intelligent, stimuli-responsive, and multifunctional nanoparticulate systems remains the sine qua non condition of any meaningful therapeutic progress in contemporary breast oncology [39].

### 2.1. Tumor Heterogeneity and Subtype-Specific Barriers (HR^+^, HER2^+^, TNBC)

Breast cancer does not constitute a unitary nosological entity, but rather a spectrum of biologically distinct tumors, for which two complementary levels of classification should be distinguished. In routine clinical practice, tumors are stratified into receptor-defined groups on the basis of the immunohistochemical (IHC) assessment of estrogen receptor (ER) and progesterone receptor (PR) expression [40] and the IHC and in situ hybridization assessment of human epidermal growth factor receptor 2 (HER2) status [41]: hormone receptor-positive/HER2-negative (HR^+^/HER2^−^), HER2-positive (either HR-positive or HR-negative), and triple-negative breast cancer (TNBC; ER^−^/PR^−^/HER2^−^). More recently, HER2-low and HER2-ultralow categories have also acquired therapeutic relevance following the introduction of HER2-directed antibody–drug conjugates [42]. These clinical groups are not equivalent to the intrinsic molecular subtypes defined by gene-expression profiling, namely luminal A, luminal B, HER2-enriched, and basal-like [7,8], and the correspondence between the two classification systems is only partial: not all clinically HER2-positive tumors belong to the HER2-enriched intrinsic subtype and, although most TNBCs are basal-like, TNBC is itself molecularly heterogeneous [7,10]. Because therapeutic decisions and most DDS studies rely on receptor status, the following paragraphs are organized according to these clinically defined groups, each of which is associated with a distinct profile of tumor progression, therapeutic response, and resistance mechanisms, with direct implications for the efficacy of drug delivery systems.

The HR^+^/HER2^−^ group accounts for approximately 70% of all breast cancers and is characterized by a functional dependence on estrogenic signaling pathways, conferring a phenotype that is generally less aggressive than that of the other groups [6,8]. Compensatory activation of the PI3K/AKT/mTOR pathway and cyclin-dependent kinases (CDK4/6) confers a proliferative advantage independent of estrogenic stimulation, generating intrinsic or acquired resistance to endocrine therapy and to therapeutic vectors targeting hormone receptors [15].

The HER2-positive group, defined by ERBB2 gene amplification or protein overexpression of the HER2 receptor, theoretically offers a well-individualized molecular target for directed delivery systems, with the paradigm of trastuzumab-based therapy and antibody-drug conjugates (ADCs) such as trastuzumab deruxtecan successfully illustrating this principle [42]. Nevertheless, the barriers specific to this group remain considerable and multidimensional. HER2 expression is not uniform within the tumor mass: intratumoral variability in antigenic density leads to heterogeneous internalization of ligand-nanoparticle complexes, reducing intracellular concentration and favoring the emergence of resistant subclones with reduced or absent receptor expression. Patients with HER2-positive disease also exhibit an increased risk of brain metastases, a context in which the blood–brain barrier adds an additional layer of complexity to the design of delivery systems, necessitating specialized strategies based on receptor-mediated transcytosis [43].

The triple-negative group (TNBC) remains the most challenging from the perspective of pharmacological delivery, representing approximately 10% of all breast cancers [6] and disproportionately affecting young or premenopausal women, who are frequently diagnosed at advanced stages [9,10]. Defined by the concomitant absence of ER, PR, and HER2 expression, this group is deprived of any conventional hormonal or anti-HER2 therapeutic option, leaving cytotoxic chemotherapy as the principal therapeutic pillar, with all its well-recognized limitations [9,10]. The profound molecular heterogeneity of TNBC, underpinned by frequent mutations in BRCA1/2 and TP53 genes and marked genomic instability, directly translates into the absence of universally applicable therapeutic targets [44,45]. Taken together, these characteristics create an environment in which conventional nanoparticulate systems are rapidly degraded, phagocytosed, or eliminated prior to achieving the requisite intracellular therapeutic concentration [46]. Beyond this classification, tumor heterogeneity itself represents a major challenge for breast cancer treatment. Intertumoral heterogeneity, that is, the biological differences between tumors of different patients, even within the same clinical group, results in markedly different responses to the same therapy [7,8]. Intratumoral heterogeneity, arising from the coexistence of genetically and phenotypically distinct subclones within a single tumor, means that receptor expression, proliferative activity, and drug sensitivity may vary between regions of the same lesion; thus, a single biopsy may not fully represent the disease and a therapy effective against the dominant clone may spare minor resistant populations [10,18]. This heterogeneity is also dynamic: under therapeutic pressure, clonal selection and evolution can generate resistant subpopulations, as illustrated by the emergence of ESR1 mutations during endocrine therapy [14], while phenotypic plasticity, including epithelial–mesenchymal transition and the acquisition of stem-like features, allows tumor cells to adapt to treatment [16,17]. In addition, the receptor status of metastatic lesions may differ from that of the primary tumor, which is why biopsy of metastatic lesions is recommended when feasible [8]. Finally, heterogeneity extends to the tumor microenvironment, in which uneven vascular perfusion, stromal density, and immune infiltration create regions with different levels of drug exposure [11,12]. For drug delivery systems, these features imply that strategies relying on a single molecular target or on uniform passive accumulation may fail to reach all malignant cell populations, providing a rationale for multi-targeted, combination, and microenvironment-modulating approaches, as discussed in the following sections.

Viewed collectively, these clinically defined groups and the multiple levels of tumor heterogeneity illustrate the fundamental principle that the efficacy of any drug delivery system is contingent not only upon its intrinsic physicochemical properties, but also upon the specific biology of the target tumor.

### 2.2. Tumor Microenvironment

The tumor microenvironment (TME) represents the complex and dynamic ensemble of non-neoplastic cells, signaling molecules, and structural components that surround and sustain the entire tumor mass, and constitutes one of the most formidable barriers to the efficacy of drug delivery systems [47]. In contrast to normal tissues, in which vascular, extracellular matrix, and immunological organization follows well-defined physiological principles, the tumor TME is characterized by profound structural and functional disorganization, generated and perpetuated by bidirectional interactions between neoplastic cells and their stromal components [47,48]. This disorganization is not incidental, but rather the result of an active evolutionary process through which the tumor remodels its microenvironment to ensure its own survival, proliferation, and evasion of host defense mechanisms [48].

In the context of breast cancer, the TME exhibits particular complexity, conditioned both by the molecular subtype and by disease stage. The direct consequence for nanoparticulate delivery systems is that they simultaneously confront obstacles of a physical, metabolic, and immunological nature, acting synergistically to limit penetration, uniform distribution, and therapeutically effective intracellular drug release [49]. Beyond its physical components, the breast cancer TME contains a heterogeneous population of immune and stromal cells that actively contribute to therapeutic failure [11,12]. Tumor-associated macrophages (TAMs), which are frequently polarized toward an immunosuppressive, pro-tumoral (M2-like) phenotype, promote angiogenesis, invasion, and metastasis, secrete cytokines and growth factors that protect tumor cells from chemotherapy-induced cell death, and suppress antitumor T-cell responses [11,46]. Myeloid-derived suppressor cells (MDSCs) and regulatory T cells (Tregs) further inhibit effector T-cell and natural killer (NK)-cell activity, while immunosuppressive metabolites, such as extracellular adenosine generated by ectonucleotidases, reinforce this immunosuppressive state [12,48]. The density and composition of tumor-infiltrating lymphocytes also differ among breast cancer subtypes, being generally higher in TNBC and HER2-positive disease than in HR^+^/HER2^−^ tumors, which are frequently immunologically “cold” [13,47]. Together with cancer-associated fibroblasts (Section 2.2.3) and hypoxia (Section 2.2.2), these cellular components hinder treatment in several ways: they reduce the efficacy of cytotoxic chemotherapy and immune checkpoint inhibitors, contribute to acquired resistance, and influence the response to treatment [46,49]. From a drug delivery perspective, macrophages within the TME and in the mononuclear phagocyte system also internalize a substantial fraction of administered nanoparticles, thereby reducing the dose available to tumor cells [37]; conversely, these same cell populations represent potential therapeutic targets for immunomodulatory nanocarriers (Section 5.3).

#### 2.2.1. Aberrant Vascularization

Tumor vascularization represents one of the most distinctive and extensively studied pathological features of the neoplastic microenvironment and constitutes a primary barrier to the efficient delivery of therapeutic agents within the tumor mass. The tumor vascular network is formed through processes of disorganized angiogenesis, initiated and perpetuated by the uncontrolled secretion of vascular endothelial growth factor (VEGF) and platelet-derived growth factor (PDGF), secreted by neoplastic cells during rapid proliferation and by stromal tumor components [50]. The result of this aberrant angiogenic process is a profoundly dysfunctional vascular network, characterized by exacerbated tortuosity, chaotic branching, non-uniform caliber, deficient pericyte coverage, and thickened, discontinuous basement membranes.

The pathophysiological consequences of this disordered vascular architecture are multiple and interdependent. First, intratumoral blood flow is profoundly heterogeneous: hypervascular tumor regions coexist with hypoperfused or even avascular zones, in which neoplastic cells survive under severe conditions of oxygen and nutrient deprivation, developing aggressive and resistant phenotypes [51]. This non-uniform distribution of perfusion drastically limits the access of systemically administered drugs, including therapeutic nanoparticles, to all tumor regions, generating heterogeneous distribution and subpopulations of cells insufficiently exposed to therapeutically effective concentrations [51,52].

Furthermore, the increased vascular permeability, resulting from loose inter-endothelial junctions and pericyte deficiency, underlies the enhanced permeability and retention (EPR) effect, classically exploited in nanomedicine as a mechanism of passive preferential accumulation of nanoparticles within tumor tissue [52]. Nonetheless, recent research has significantly challenged the practical translational value of the EPR effect in the human clinical context. Solid human tumors exhibit marked heterogeneity in vascular conductivity, with non-uniform permeability, resulting in heterogeneous pore size distribution and, consequently, heterogeneous nanoparticle extravasation and delivery [53]. In a widely cited meta-analysis of preclinical studies, Wilhelm et al. reported a median nanoparticle delivery efficiency of approximately 0.7% of the injected dose (%ID) across 232 heterogeneous datasets obtained with different nanoparticle types, tumor models, and sampling times [54]. This value should be regarded as a heterogeneous preclinical benchmark rather than as a universal or clinical estimate of nanoparticle delivery to human breast tumors, particularly since the underlying time-averaged %ID metric has subsequently been questioned (see Section 3.1).

#### 2.2.2. Hypoxia and Acidic pH

Hypoxia represents one of the most universal and best-documented characteristics of the solid tumor microenvironment, including in breast cancer, and constitutes a major determinant of biological aggressiveness, therapeutic resistance, and the failure of drug delivery systems [55]. Its pathological origin resides in the fundamental imbalance between the accelerated oxygen consumption of rapidly proliferating neoplastic cells and the profoundly deficient supply capacity of the aberrant tumor vasculature, which fails to ensure uniform and efficient tissue perfusion. The direct consequence is the emergence of extensive hypoxic zones, with partial oxygen tensions below 10 mmHg, distributed non-uniformly within the tumor mass and coexisting with relatively normoxic regions situated in proximity to functional vessels [55,56].

**Figure 1:**
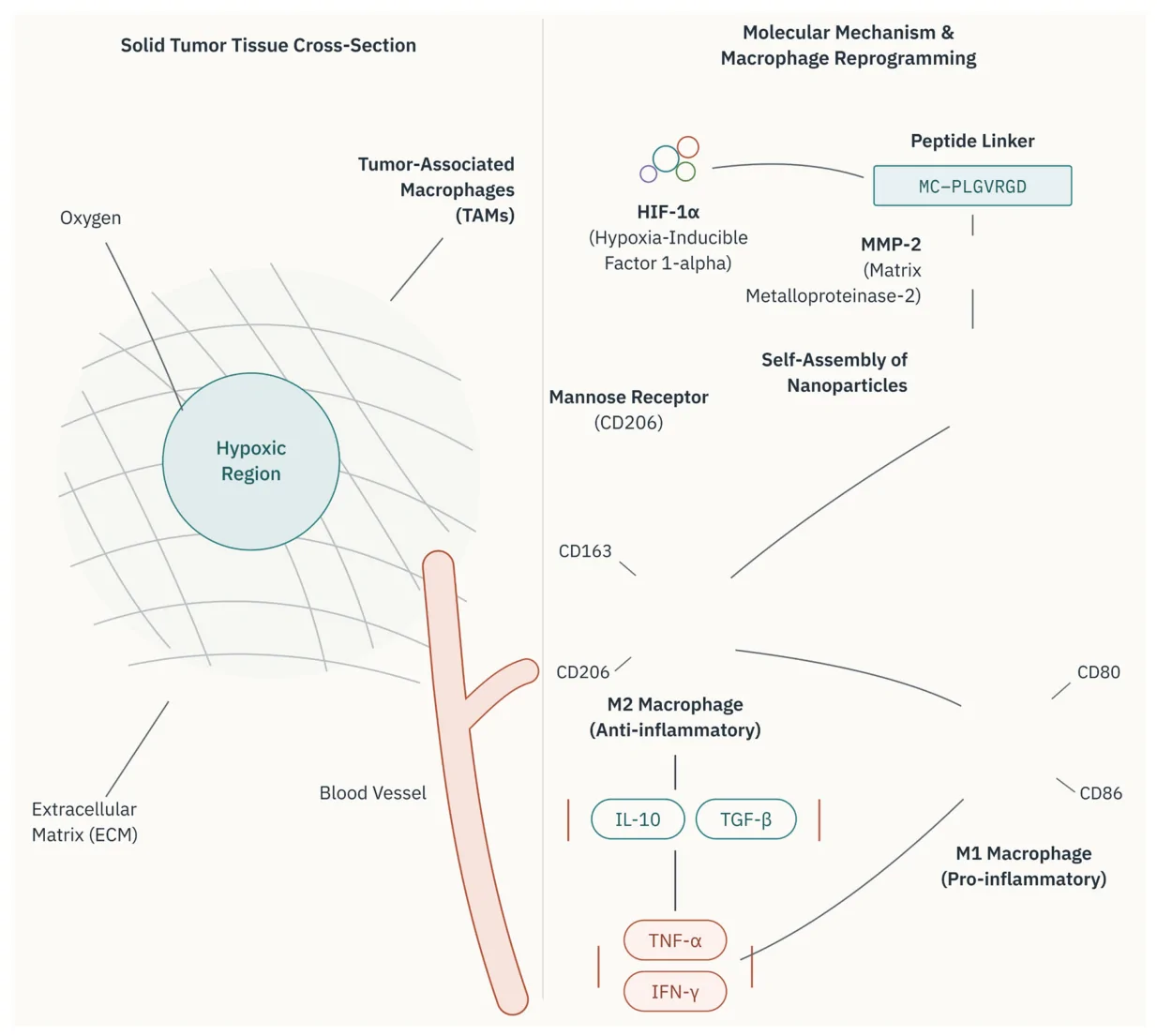
Schematic illustration of hypoxia-triggered self-assembly. Adapted from [52].

The cellular response to hypoxia is centrally orchestrated by hypoxia-inducible factor 1-alpha (HIF-1α), a transcription factor selectively stabilized under conditions of oxygen deficiency [56]. In hypoxic tumor cells, HIF-1α initiates a broad transcriptional program with profound consequences (Figure 1): it stimulates angiogenesis through VEGF induction, reprograms cellular metabolism toward aerobic glycolysis (the Warburg effect) with massive lactate production, promotes epithelial-to-mesenchymal transition, inhibits apoptosis, and activates resistance mechanisms to chemotherapy and radiotherapy [56,57]. From the perspective of drug delivery systems, the stabilization of HIF-1α and its transcriptional consequences generate a particularly unfavorable context.

Notable among these consequences are the overexpression of ATP-binding cassette (ABC) efflux pumps, reduced membrane permeability, and alteration of nanoparticle cellular internalization pathways in hypoxic zones, collectively contributing to a dramatic reduction in therapeutic efficacy [57].

Accelerated aerobic glycolysis generates massive quantities of lactic acid which, actively exported into the extracellular space via monocarboxylate transporters and H^+^ pumps, leads to pronounced acidification of the interstitial microenvironment, with extracellular pH values ranging between 6.0 and 6.8, compared to the physiological pH of 7.4 in normal tissues [57]. This extracellular acidity exerts complex and unfavorable pharmacological effects, including modification of the ionization state of weakly basic drug molecules, reducing their membrane permeability and intracellular accumulation capacity through the phenomenon of reversed ion trapping [56]. Additionally, the acidic pH promotes the instability of certain nanoformulations, compromising the integrity of liposomal lipid bilayers and altering the surface properties of polymeric nanoparticles, with a direct impact on targeting efficiency and drug release profiles [56,57].

From the perspective of delivery system design, however, the hypoxia-acidity duality of the TME also represents a valuable strategic opportunity: these characteristics, frequently, although heterogeneously, present in solid tumors and generally less pronounced in most normal tissues, can be exploited as endogenous stimuli for stimuli-responsive delivery systems capable of releasing the drug preferentially within the tumor microenvironment [58]. pH-sensitive nanoparticles, hypoxia-triggered nanocarriers, and redox-responsive systems that exploit elevated intracellular glutathione levels, indirectly induced by hypoxia, represent rapidly expanding research directions with significant translational potential for the clinical oncology of breast cancer [58,59].

#### 2.2.3. Dense Extracellular Matrix

The tumor extracellular matrix (ECM) represents the foremost physical and biochemical obstacle to the intratumoral penetration of therapeutic agents, including nanoparticulate delivery systems, constituting a major component of microenvironmental barriers in breast cancer [60]. In contrast to the ECM of normal tissues, whose composition and three-dimensional architecture are homeostatically regulated, the tumor ECM is the product of an active desmoplastic remodeling process orchestrated predominantly by cancer-associated fibroblasts (CAFs), which deposit excessive quantities of type I and III collagen, fibronectin, laminin, hyaluronic acid, and proteoglycans, generating a dense, rigid, and extensively crosslinked matrix network.

The direct physical consequence of this matrix densification is a dramatic reduction in the porosity of the interstitial space and, consequently, in the diffusion coefficients of macromolecules and nanoparticles. Mathematical modeling and experimental studies demonstrate that the increased stiffness of the tumor ECM exerts mechanical compression upon intratumoral vessels and lymphatic channels, exacerbating the tissue ischemia and elevated interstitial pressure already generated by the aberrant vasculature [60,61]. This combined effect creates a pressure gradient that actively opposes the convective transport of nanoparticles from the circulation into the tumor interior, forcing delivery systems to rely exclusively on passive diffusion, an extremely slow and inefficient mechanism in the presence of a dense ECM [61].

A central role in the architecture and function of the tumor ECM is played by CAFs, activated stromal cells that secrete not only the structural components of the matrix, but also a broad range of soluble factors, including cytokines, CXCL12, TGF-β, and matrix metalloproteinases (MMPs), that simultaneously modulate ECM composition, tumor immunity, and the invasive behavior of neoplastic cells [62]. The action of lysyl oxidase (LOX) and lysyl oxidase-like 2 (LOXL2), enzymes secreted by CAFs and hypoxic tumor cells, catalyzes the crosslinking of collagen and elastin, consolidating matrix rigidity and perpetuating a mechanical environment incompatible with therapeutic penetration [63]. Pharmacological inhibition of LOXL2 has demonstrated not only reduced collagen deposition and attenuated CAF activity, but also partial ECM normalization and improved drug accessibility within the tumor [62,63,64].

### 2.3. Physical (Cellular and Subcellular) Barriers

Even in circumstances where a nanoparticulate system successfully overcomes the vascular and matrix barriers of the tumor microenvironment and reaches the vicinity of the target cell, it confronts a further level of obstacles, of a cellular and subcellular nature, that decisively condition ultimate therapeutic efficacy. These barriers operate at a considerably finer scale than tissue-level obstacles, governing the access of the therapeutic agent to the specific intracellular compartment (cytosol, nucleus, mitochondria, or endoplasmic reticulum) and representing the principal cause of translational failure of many nanoparticulate systems that demonstrate satisfactory tumor accumulation yet insufficient cytotoxic effect [65].

The first cellular barrier is constituted by the plasma membrane, a bilayer phospholipid structure with integral proteins, functioning as a selectively permeable barrier that precisely controls the passage of substances from the extracellular environment into the cytosol [65,66]. Nanoparticles cannot passively traverse this barrier except under exceptional circumstances. The predominant internalization pathway is endocytosis, an active, energy-dependent process through which the plasma membrane invaginates and forms intracellular vesicles that capture the nanoparticle along with its surface ligands [66]. Endocytic mechanisms are multiple and subtype-specific. Clathrin-mediated endocytosis, caveolae-mediated endocytosis, macropinocytosis, and phagocytosis represent the principal pathways through which tumor cells internalize nanoparticles, each leading to distinct intracellular compartments [66,67]. The size, shape, surface charge, and ligand functionalization of the nanoparticle determine which endocytic pathway will be preferentially activated, with direct consequences for subsequent intracellular trafficking and delivery efficiency [68].

An obstacle of critical importance, frequently underestimated in delivery system design, is the protein corona, the dynamic layer of plasma proteins adsorbed onto the nanoparticle surface immediately upon contact with the biological milieu [68]. Once introduced into the circulation or the tumor interstitial space, nanoparticles are rapidly enveloped by serum proteins, including albumin, fibrinogen, immunoglobulins, and apolipoproteins, which profoundly alter their biological identity, masking targeting ligands on their surface and modifying the specificity of interaction with cellular receptors [68,69]. The protein corona thus transforms a highly specific nanoparticle, engineered to recognize a specific tumor receptor, into a biological object with unpredictable recognition properties, frequently recognized by macrophages of the mononuclear phagocytic system and rapidly cleared from the circulation [69].

The most formidable subcellular obstacle, however, remains the endosomal trap and lysosomal degradation [69]. Once internalized via endocytosis, nanoparticles are captured within endosomal vesicles at a pH of approximately 6.0–6.5, which progressively mature into late endosomes (pH ~5.5) and lysosomes (pH ~4.5–5.0), the cellular degradation compartments. Lysosomes harbor a formidable hydrolytic enzymatic arsenal comprising proteases, nucleases, lipases, and glucosidases, which efficiently degrade their internal contents, including the therapeutic payload of nanoparticles. It is estimated that less than 2% of the therapeutic agent internalized via endocytosis ultimately reaches the free cytosol, with the remainder being degraded or re-exported via exocytosis [69,70]. For therapeutic agents with cytoplasmic or nuclear targets, including siRNA, mRNA, plasmid DNA, and kinase inhibitors, failure of endosomal escape is equivalent to complete abrogation of the therapeutic effect [70].

### 2.4. Drug Resistance and Efflux Mechanisms

Drug resistance, both primary, manifested prior to any treatment, and acquired, emerging as an adaptive response to therapeutic pressure, represents the principal cause of systemic chemotherapy failure in breast cancer and constitutes a fundamental obstacle to the efficacy of any drug delivery system, including nanoparticulate platforms [71]. The phenomenon of multidrug resistance (MDR) describes the capacity of tumor cells to simultaneously resist structurally and functionally distinct chemotherapeutic agents through the concomitant activation of multiple molecular defense mechanisms, rendering monovalent therapeutic approaches invariably of limited long-term efficacy [72].

The most thoroughly characterized mechanism with the most direct impact upon drug delivery systems is the overexpression of membrane efflux pumps belonging to the ATP-binding cassette (ABC) transporter superfamily [71]. The principal representatives implicated in breast cancer MDR are P-glycoprotein (P-gp, encoded by *ABCB1/MDR1*), multidrug resistance-associated protein 1 (MRP1/ABCC1), and breast cancer resistance protein (BCRP/ABCG2) [72]. These transmembrane proteins function as ATP hydrolysis-dependent molecular pumps that actively recognize and expel a broad range of hydrophobic molecules from the intracellular compartment into the extracellular space, dramatically reducing intracellular drug concentrations below the effective therapeutic threshold [73]. The breadth of substrate recognition, P-gp alone recognizes hundreds of structurally diverse molecules, including anthracyclines, taxanes, vinca alkaloids, and kinase inhibitors, renders the overexpression of these transporters a transversal barrier that simultaneously compromises the efficacy of the majority of cytostatic drug classes employed clinically [73,74].

Beyond efflux mechanisms, resistance to therapy in breast cancer involves a broad spectrum of additional molecular adaptations with direct impact on the intracellular concentration and activity of the therapeutic compound. Lysosomal drug sequestration, a phenomenon whereby weakly basic molecules are captured and inactivated within acidic lysosomal compartments—rendered unable to reach their cytoplasmic or nuclear target— constitutes a frequently underestimated but clinically significant resistance mechanism [74]. Dysregulation of apoptotic signaling pathways, through overexpression of Bcl-2 family anti-apoptotic proteins and downregulation of effector caspases, reduces tumor cell sensitivity to drug-induced cell death even when intracellular concentrations are adequate [75]. Enhanced DNA repair capacity neutralizes the cytotoxic effect of agents inducing genotoxic lesions, including cisplatin, anthracyclines, and taxanes, through rapid and efficient repair of double-strand breaks and DNA adducts.

## 3. Design Principles of DDS for Breast Cancer

**Figure 2:**
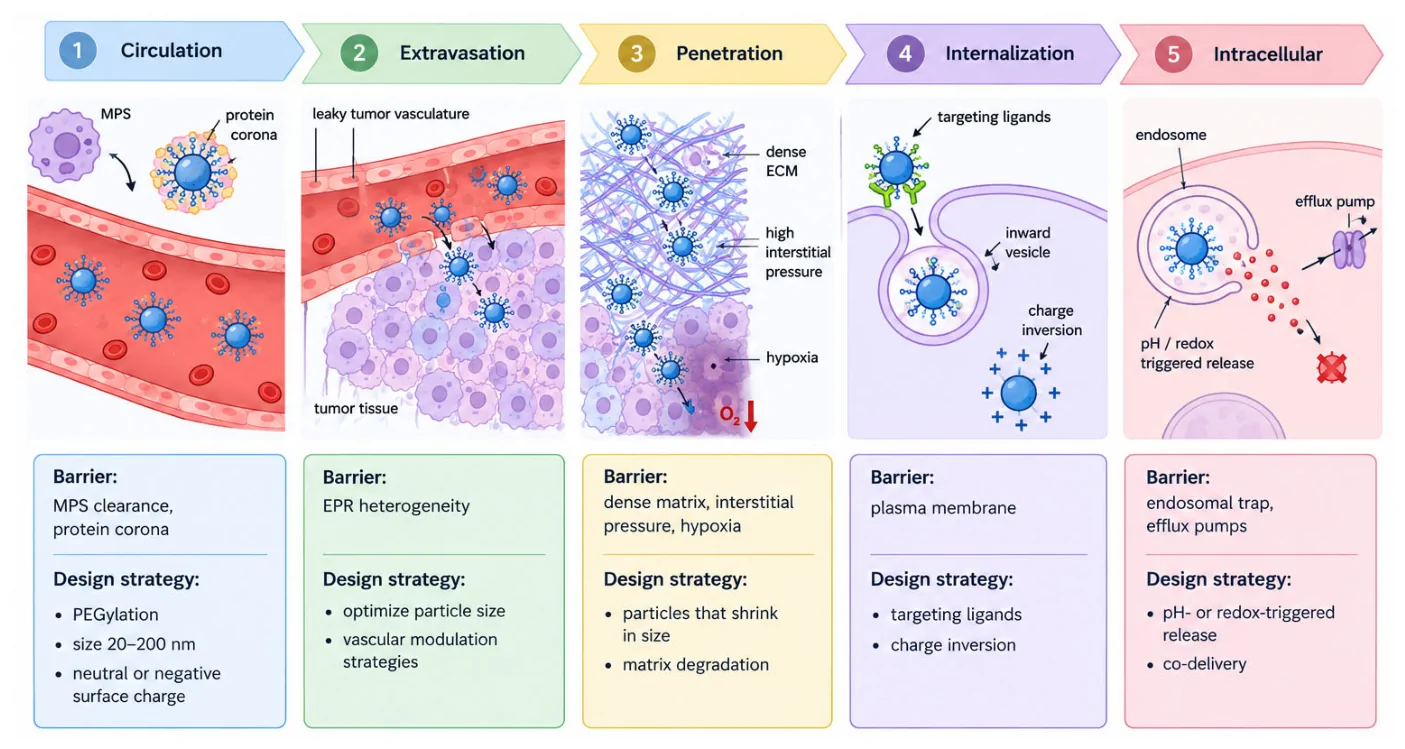
Sequential biological barriers encountered by drug delivery systems in breast cancer and corresponding design strategies. From systemic circulation to intracellular drug release, each barrier (upper row) imposes specific requirements on carrier design (lower row): (1) opsonization and clearance by the mononuclear phagocyte system (MPS); (2) heterogeneous extravasation from tumor vessels; (3) penetration through the dense extracellular matrix (ECM) under elevated interstitial fluid pressure (IFP) and hypoxia; (4) heterogeneous receptor expression on tumor cells; and (5) endolysosomal entrapment and drug efflux.

Before discussing specific design parameters, it is useful to outline how DDS can address the main limitations of conventional breast cancer therapy. First, the unfavorable physicochemical and pharmacokinetic properties of many anticancer drugs, such as low aqueous solubility, limited stability, and rapid clearance, can be improved by encapsulation, which protects the cargo, avoids toxic solubilizing excipients, and prolongs systemic circulation [26,28]. Second, insufficient selectivity and the resulting dose-limiting toxicity can be mitigated by modifying drug biodistribution, as exemplified clinically by the reduced cardiotoxicity of liposomal doxorubicin and the solvent-free formulation of nab-paclitaxel (Section 7) [28]. Third, limited and heterogeneous tumor penetration may be partially overcome by optimizing carrier size and surface properties or by microenvironment-modulating strategies, although the heterogeneity of the EPR effect remains a major constraint [23,24,25]. Fourth, resistance mechanisms, including drug efflux and compensatory survival signaling, may be addressed by co-delivering synergistic agents at defined ratios or by ligand-mediated internalization, although clinical evidence for these approaches remains limited [18,26]. Finally, controlled and stimulus-responsive release may improve the spatial and temporal control of drug exposure [31,32]. Importantly, these potential advantages are not intrinsic to nanocarriers but depend on rational design, which is the focus of the following subsections (Figure 2).

The ensemble of barriers described necessitates an integrated and multidimensional approach to drug delivery system design, with each identified obstacle translating into specific technical requirements for the nanoparticulate vector [76]. Nanoparticle size, surface charge, geometry, and surface characteristics represent the primary physicochemical parameters governing simultaneously systemic circulation, tumor extravasation, matrix penetration, and cellular internalization efficiency, parameters that frequently carry contradictory requirements and necessitate rational trade-offs and context-specific, subtype-tailored optimization [77].

Active targeting strategies employing molecular ligands, stimuli-responsive platforms exploiting the acidic pH, hypoxia, or elevated intracellular glutathione levels of the TME, as well as biomimetic systems coated with natural cell membranes, represent the leading directions in nanomedicine applied to breast cancer. These approaches offer solutions to overcome tumor heterogeneity, the limited EPR effect, and multidrug resistance mechanisms [78]. The simultaneous co-delivery of cytotoxic agents with efflux pump inhibitors or gene-silencing molecules within a single multifunctional vector enables the concomitant neutralization of multiple resistance mechanisms, thereby restoring the therapeutic sensitivity of neoplastic cells.

**Figure 3:**
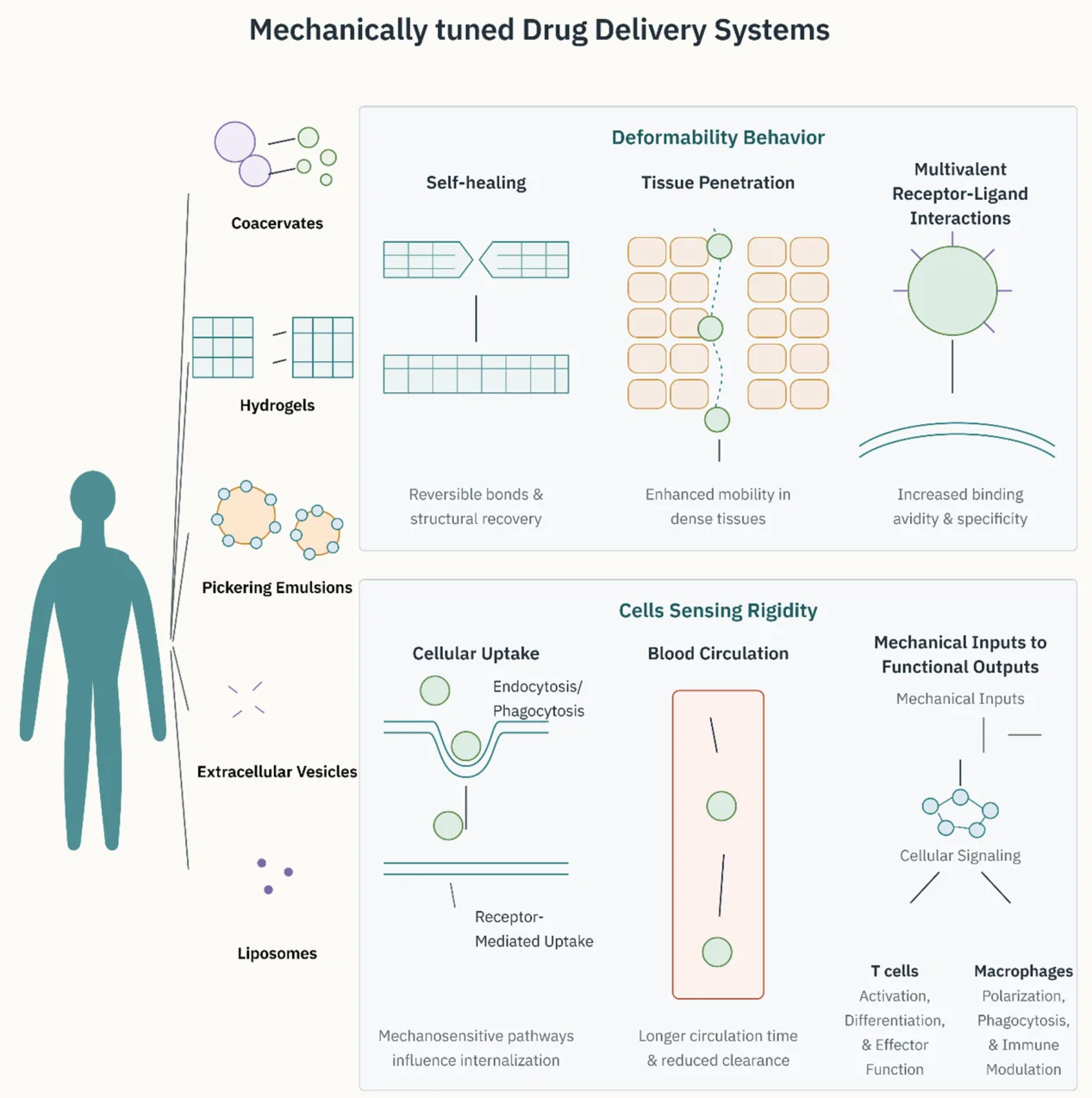
Schematic illustration of representative mechanically tuned DDS and their mechanisms of action. Adapted from [81].

The rational design of DDS for breast cancer represents a synthetic discipline that integrates in-depth knowledge of tumor biological barriers with rigorous engineering and pharmaceutical principles, with the aim of generating therapeutically effective, selective, and clinically translatable vectors [79,80]. Every design decision, from nanoparticle size and morphology to surface chemistry, active pharmaceutical ingredient release mechanism, and targeting strategy, is governed by the necessity of sequentially overcoming multiple, interdependent biological obstacles (Figure 3). From the vascular and matrix barriers of the tumor microenvironment to cellular and subcellular impediments, all of these collectively condition ultimate therapeutic efficacy [81].

### 3.1. Passive vs. Active Targeting: Current Evidence and Controversies

The dichotomy between passive and active nanoparticle targeting has structured the fundamental debate in oncological nanomedicine for three decades, generating both remarkable clinical successes and profound translational disappointments that have necessitated a critical re-evaluation of established paradigms [82]. A nuanced understanding of the mechanisms, limitations, and opportunities of each strategy, within the specific context of breast cancer biology, with its subtype-level heterogeneity and tumor microenvironment complexity, constitutes the prerequisite for any DDS design approach with genuine clinical relevance.

Passive targeting relies exclusively on the exploitation of the intrinsic pathophysiological properties of tumor vasculature, without involving any specific molecular interaction between the nanoparticle and the target cells. The fundamental principle of this strategy is the enhanced permeability and retention (EPR) effect, initially described by Matsumura and Maeda in 1986 and established as the cornerstone of the first generation of oncological nanomedicine.

Nevertheless, the limitations of passive targeting have become increasingly apparent. The frequently quoted value of approximately 0.7% ID, reported by Wilhelm et al. as the median across 232 preclinical datasets [54], illustrates the modest and highly variable tumor delivery observed in animal models. However, this value was derived from a non-standard, time-averaged %ID in tumor metric, and a subsequent reanalysis using classical pharmacokinetic parameters showed that this metric correlated poorly with standard measures of tumor exposure, with relative tumor delivery being approximately 100-fold greater when assessed by the AUC__tumor_/AUC__blood_ ratio than by %ID in tumor [82]. Consequently, the 0.7% figure should not be interpreted as the fraction of the injected dose delivered to the tumor, nor extrapolated to patients; it is best regarded as a heterogeneous preclinical benchmark. In the clinical setting, EPR-mediated accumulation varies markedly between and within patients, depending on tumor vascularization, stromal ECM density, interstitial fluid pressure, and disease stage, parameters that cannot be accurately predicted without individualized prior imaging evaluation [23,25].

Active targeting was conceptualized as a solution to the inherent limitations of the passive strategy, through the chemical or physical conjugation of specific molecular ligands, including monoclonal antibodies or Fab fragments, recognition peptides, aptamers, and small molecules, to the nanoparticle surface, enabling selective recognition of receptors overexpressed on the tumor cell membrane and preferential internalization via receptor-mediated endocytosis [83]. The targets most frequently exploited for this purpose in breast cancer differ substantially in their level of validation: HER2 is a clinically validated therapeutic target, whereas the folate receptor alpha (FOLR1), the transferrin receptor, CD44, and integrins such as αvβ3 and α5β1 remain experimental delivery targets whose utility has been demonstrated mainly in preclinical models (Section 5.1). Importantly, in preclinical models of HER2-positive breast cancer, anti-HER2 immunoliposomes showed enhanced antitumor efficacy compared with non-targeted liposomes [84], yet antibody targeting did not increase the overall tumor accumulation of long-circulating lipidic nanoparticles; instead, it increased their internalization by tumor cells [83].

The central controversy, however, concerns the extent to which active targeting genuinely adds value over passive targeting under real clinical conditions [83]. Critical arguments highlight that active targeting does not significantly improve overall intratumoral accumulation compared to non-targeted nanoparticles of similar dimensions. The principal transport mechanism remains passive extravasation via the EPR effect; active targeting rather selectively improves the cellular internalization step once the nanoparticle has reached the perivascular tumor space. Accordingly, the true benefit of active targeting manifests predominantly at the cellular and subcellular level, rather than at the level of systemic transport [83,85]. A further obstacle is constituted by protein corona formation, which can completely mask or alter the functionality of surface ligands under physiological conditions, dramatically diminishing the specificity of target receptor recognition and nullifying the theoretical advantage of molecular conjugation [85].

The emerging consensus in contemporary oncological nanomedicine transcends the binary opposition between the two strategies, promoting, instead, their complementary and synergistic application. Passive targeting, through optimization of physicochemical parameters and prolongation of circulation time, ensures intratumoral accumulation, while active targeting optimizes specific cellular internalization and drug release in the relevant intracellular compartment [85]. Hybrid platforms integrating both mechanisms, such as PEGylated nanoparticles bearing surface ligands concealed beneath an external layer cleavable within the tumor microenvironment, which exposes the active ligands exclusively after intratumoral accumulation, represent the leading-edge approach, combining the advantages of prolonged circulation characteristic of passive targeting with the cellular specificity of active targeting [85,86]. In the context of breast cancer, this integrative approach becomes indispensable given the marked subtype-level heterogeneity, which necessitates adaptation of the targeting strategy to the receptor-specific profile of each individual tumor, with genuine prospects for molecular-level therapeutic personalization [86].

### 3.2. Size, Shape, Surface Charge, and Protein Corona Effects

The physicochemical properties of nanoparticles—size, shape, surface charge, and chemical composition of the outer coating—are not merely descriptive attributes of the nanoparticulate material, but constitute primary functional determinants governing every stage of the biological journey of the DDS, from systemic circulation and vascular extravasation, to intratumoral penetration, cellular internalization, and ultimately drug release within the intracellular compartment [87]. Understanding the relationship between these properties and biological behavior under physiological conditions represents the foundation of rational design in modern oncological nanomedicine.

Nanoparticle size exerts simultaneous and often contradictory influence upon the multiple biological barriers that must be sequentially overcome. From the perspective of tumor extravasation via the EPR effect, the optimal size range lies between 20 and 200 nm, with the endothelial junctions of tumor vasculature permitting preferential passage of nanoparticles with diameters below 400–600 nm [88]. However, deep penetration into the tumor parenchyma, beyond the immediate proximity of peripheral vessels, requires significantly smaller dimensions: studies on three-dimensional tumor spheroid models have demonstrated that nanoparticles with diameters below 50–60 nm penetrate substantially deeper into tumor architecture compared to those of 100–150 nm, which remain sequestered in perivascular layers. At the opposite extreme, nanoparticles with diameters below 8 nm are subject to rapid renal clearance, with an extremely short plasma half-life incompatible with efficient intratumoral accumulation [87,88]. This tension between the requirement for prolonged circulation, favored by intermediate sizes of 100–200 nm, and that of deep matrix penetration, necessitating dimensions below 50 nm, has stimulated the design of nanoparticles with dynamic size behavior, capable of reducing their dimensions in situ under the action of tumor enzymatic or pH stimuli, transitioning from a circulation-favorable to a matrix penetration-favorable profile [88].

Nanoparticle shape profoundly and often counter-intuitively influences systemic distribution and cellular behavior. Spherical nanoparticles exhibit the longest overall circulation time and the highest levels of total tumor accumulation due to their minimal interaction with vascular endothelium and splenic macrophages [89]. Elongated geometries, such as rods, nanotubes, and discs, demonstrate distinct transport behavior, with a tendency to adopt preferential orientations relative to blood flow and to interact differently with cell membrane receptors, potentially accelerating cellular internalization through membrane curvature-mediated endocytosis. Comparative studies on nanosystems of identical size but differing morphology, such as nanospheres vs. nanorods, have demonstrated that spherical nanoparticles offer the most favorable profile for breast cancer in murine models, combining optimal circulation time with superior intratumoral accumulation [89,90].

Surface charge critically conditions both interactions with extracellular biological components and internalization efficiency. Cationic nanoparticles interact electrostatically with the negatively charged plasma membrane of tumor cells, facilitating adsorption and endocytosis, but also promoting rapid opsonization by serum proteins and recognition by macrophages of the mononuclear phagocyte system, with accelerated clearance from the circulation and significant non-specific toxicity [90]. Anionic nanoparticles exhibit a more favorable circulation profile and a reduced tendency toward aggregation but interact less strongly with the cell membrane. Systematic studies on three-dimensional tumor spheroids have paradoxically demonstrated that negatively charged nanoparticles achieve superior intratumoral accumulation and penetration compared to positively charged or neutral counterparts, possibly through preferential interactions with the glycosaminoglycan-rich extracellular matrix and more efficient evasion of macrophage clearance. Neutral nanoparticles, frequently obtained by PEGylation, exhibit the longest systemic circulation time by virtue of reduced opsonization, but at the cost of diminished specific interactions with target cells [90].

The protein corona represents one of the most important and most difficult to control phenomena shaping the in vivo behavior of DDS [90,91]. The composition of the protein corona is simultaneously determined by the physicochemical properties of the nanoparticle (size, charge, hydrophobicity, surface functionalization) and by the composition of the local biological milieu (plasma, tumor interstitial fluid, intracellular microenvironment), varying dynamically as the nanoparticle progresses through different biological compartments [92]. The dominant corona proteins include albumin, fibrinogen, immunoglobulins, and apolipoproteins, which can either opsonize nanoparticles, facilitating phagocytosis, or function as endogenous ligands, mediating specific interactions with cellular receptors. From the perspective of active targeting, the protein corona constitutes a fundamental obstacle: through adsorption onto the nanoparticle surface, it can completely mask conjugated molecular ligands (antibodies, peptides, aptamers), abolishing the recognition specificity for target receptors and transforming a rationally engineered, highly specific nanoparticle into a biological object with unpredictable behavior [92].

### 3.3. Stimuli-Responsive Systems (pH, Redox, Enzymatic, Thermal)

Stimuli-responsive nanoparticulate systems represent one of the most extensively investigated paradigms in oncological nanomedicine, designed to exploit biochemical and physical characteristics of the tumor microenvironment as endogenous stimuli for the controlled and preferential release of the therapeutic agent in proximity to, or within, neoplastic cells [92]. Unlike conventional delivery systems, which may release the drug continuously throughout the systemic circulation, stimuli-responsive nanoparticles are designed to remain relatively stable under normal physiological conditions and to release the drug preferentially upon encountering signals that are enriched, although not exclusive, in the tumor microenvironment [92]. This property may improve their selectivity profile and reduce systemic toxicity, with potential implications for the therapeutic index of the cytotoxic compounds employed in breast cancer [92]. It should be emphasized, however, that none of these endogenous triggers is tumor-specific: acidosis, hypoxia, protease activity, and elevated glutathione levels are spatially and temporally heterogeneous within tumors and may also occur in normal or pathological tissues, including inflamed, ischemic, or regenerating tissues, as well as in organs with physiologically high glutathione content, such as the liver and kidney. Stimulus-responsive release should therefore be regarded as a means of increasing, rather than guaranteeing, tumor selectivity.

pH-responsive systems are the most extensively studied and most advanced in terms of clinical translation, exploiting the significant pH gradient existing between normal physiological compartments (pH 7.4) and the tumor extracellular microenvironment (pH 6.0–6.8), as well as intracellular lysosomal compartments (pH 4.5–5.5) [92,93,94]. The molecular mechanisms of response to pH variations include protonation and deprotonation of amine or carboxyl groups within the structure of amphiphilic polymers, such as poly(β-amino esters) (PBAE) or polyacrylic acid, inducing conformational changes, micelle dissolution, or disintegration of the nanoparticulate matrix with consecutive drug release [93]. Charge-reversal nanoparticles, which carry a negative charge at physiological pH to avoid opsonization yet become positively charged at the acidic pH of the tumor microenvironment, thereby promoting membrane interaction and endocytosis, represent one of the most promising strategies for combining a favorable pharmacokinetic profile with efficient cellular internalization [93,94]. Acid-labile linkages within the structure of prodrugs or molecular linkers, including hydrazones, acetals, and citraconimides, are preferentially cleaved at endosomal or tumor extracellular pH, releasing the active drug predominantly within the compartments of interest. An important limitation of pH-responsive systems is the intratumoral variability of the pH gradient, which is not uniformly distributed throughout the entire tumor mass, and partial overlap with pH values found in certain inflamed or hypoxic normal tissues, which may generate nonspecific drug release [93,94].

Redox-responsive systems exploit the redox gradient existing between the extracellular environment and the cytoplasm of tumor cells. Intracellular glutathione (GSH) concentrations reach approximately 2–10 mM, 100- to 1000-fold higher than extracellular concentrations of 2–20 μM [94]; this gradient is also present in normal cells, although GSH levels are frequently further elevated in tumor cells. This differential is exploited as a stimulus for the cleavage of disulfide bonds within the nanoparticle structure: –S–S– linkages remain relatively stable in the weakly oxidizing extracellular environment but are rapidly cleaved by intracellular GSH, leading to nanoparticle disintegration and intracellular drug release. Integration of disulfide linkages into the polymer backbone of nanoparticles or at the lipid–nucleus interface of lipid–polymer nanosystems has enabled the development of platforms with efficient intracellular release, demonstrated in preclinical models of doxorubicin-resistant TNBC breast cancer. A potential advantage of redox-responsive systems is that drug release is triggered predominantly within the reducing intracellular compartment. However, elevated glutathione is not restricted to tumor cells, as millimolar intracellular concentrations are also present in normal cells, particularly in the liver, and glutathione levels vary considerably among and within tumors. Moreover, intracellular release does not by itself demonstrate endosomal escape or avoidance of efflux transporters; such claims require direct experimental evidence, for example, subcellular colocalization studies or evaluation in P-gp-overexpressing resistant models [94,95].

Enzymatically responsive systems constitute a category of high molecular precision, based on the overexpression within the tumor microenvironment of specific enzymes, including matrix metalloproteinases (MMP-2, MMP-9), lysosomal cathepsins, phospholipases, and hyaluronidases, which cleave peptide sequences or polysaccharide structures from the nanoparticle coating, triggering the exposure of targeting ligands, dimensional modification, or direct release of the therapeutic agent [60]. Peptides cleavable by MMP-2 and MMP-9, enzymes frequently overexpressed in the stroma of invasive breast tumors and in TNBC but also active during physiological tissue remodeling and inflammation, have been employed as coupling agents between the outer shell of PEGylated nanoparticles and the drug compound, enabling cleavage of the PEG layer within the tumor microenvironment concomitant with exposure of cell-penetrating functionalities [95]. Hyaluronate-functionalized systems respond to tumor hyaluronidase through depolymerization of the hyaluronic acid coating and simultaneous exposure of anti-CD44 ligands, combining enzymatic responsiveness with active targeting [95,96].

Thermoresponsive systems exploit the phase transition of polymers with a lower critical solution temperature (LCST), most commonly poly(N-isopropylacrylamide) (PNIPAAm) and its derivatives, which undergo a reversible sol–gel transition at temperatures slightly above normal body temperature (32–37 °C), calibrated to respond to localized intratumoral hyperthermia [96]. Combination of thermoresponsive systems with hyperthermia induced by magnetic nanoparticles or radiofrequency radiation generates advanced theranostic platforms in which drug release is synchronized with the direct thermal cytotoxic effect [96].

### 3.4. Pharmacokinetics, Biodistribution, and Tumor Penetration

Pharmacokinetics, biodistribution, and tumor penetration constitute an interdependent functional triad whose components decisively determine whether a nanoparticulate system, designed with pharmaceutical rigor, will or will not succeed in exerting a real therapeutic effect at the level of the tumor mass [97]. Even systems with optimal physicochemical properties and sophisticated molecular targeting strategies may fail clinically if their pharmacokinetic profile is incompatible with sustained intratumoral accumulation. Equally, when intratumoral penetration remains restricted to perivascular layers without reaching the deep, hypoxic cell subpopulations with high therapeutic resistance potential, the cumulative effect falls below the therapeutic threshold [97].

The systemic biodistribution of nanoparticles is dominated, in the absence of effective strategies, by preferential sequestration in organs of the mononuclear phagocyte system, liver, spleen, and lung, where resident macrophages rapidly recognize and internalize opsonized nanoparticles, reducing the fraction available for intratumoral accumulation [98]. Quantitative assessment of biodistribution by nuclear imaging techniques, using radiolabeled nanoparticles with technetium-99m or positron emission tomography isotopes, provides irreplaceable spatiotemporal information regarding the biological trajectory of therapeutic vehicles under in vivo conditions, enabling quantification of the fraction of the injected dose that achieves significant concentrations within tumor tissue and various elimination organs [99]. Biodistribution studies with methotrexate-loaded chitosan nanoparticles radiolabeled with [^99m^Tc] demonstrate that nanoparticulate vectors achieve tumor-to-muscle ratios significantly superior to those of conventional free preparations, confirming that nanometric encapsulation favorably alters the tissue distribution profile and increases accumulation specificity at the tumor site [100].

Hyaluronidase (HAase), an FDA-approved enzyme with selective degrading activity against hyaluronic acid in the extracellular matrix, has proven to be an effective adjuvant for intratumoral penetration, facilitating the diffusion of drugs and nanoparticulate vectors into tumor depth through reduction in the physical barrier posed by the hyaluronic acid-rich ECM. Innovative systems integrating HAase with cytotoxic agent-loaded nanoparticles within injectable hydrogel matrices have demonstrated both superior intratumoral penetration compared to conventional formulations and prolonged drug retention at the tumor site [101]. Enzymatically responsive nanoparticulate platforms exploit the selective overexpression of γ-glutamyltranspeptidase (GGT) in the tumor microenvironment as a surface charge-reversal stimulus. The nanoparticles circulate with a neutral/negative profile favorable for avoiding macrophage clearance and acquire a positive charge exclusively at the tumor site, facilitating electrostatic interaction with the cell membrane and penetration of the deep tumor stroma [102]. Near-infrared imaging has confirmed that these enzymatically responsive nanoplatforms achieve fluorescence gradients from the tumor periphery to the hypoxic center that are significantly superior to those of non-responsive nanoparticles or free agents, demonstrating that the enzyme-mediated penetration strategy overcomes the desmoplastic barrier characteristic of TNBC more effectively than passive accumulation via the EPR effect [103].

### 3.5. Safety, Immunogenicity, and Biodegradability Considerations

Assessment of the safety profile of nanoparticulate systems constitutes a mandatory and frequently limiting condition for their clinical translation, as the complexity of nanoparticle interactions with the immune system, elimination organs, and healthy tissues can generate unpredictable adverse effects not captured by conventional in vitro studies [104]. The immune response to nanoparticles manifests through two principal mechanisms: activation of the complement system, including the complement activation-related pseudoallergy (CARPA) reaction, characteristic of liposomal formulations and frequently observed clinically in the form of acute infusion-associated reactions with Doxil^®^ or Abraxane^®^, and recognition by the mononuclear phagocyte system, with sequestration of nanoparticles in the liver, spleen, and bone marrow, reducing the fraction available for intratumoral accumulation [105]. The immunogenicity of surface ligands, particularly conjugated monoclonal antibodies or protein fragments, can induce the formation of anti-drug antibodies that neutralize therapeutic efficacy upon repeated administration, a problem of direct relevance in the chronic therapeutic regimens of breast cancer [106].

The biodegradability of the nanoparticulate material involves the elimination kinetics and the potential for long-term tissue accumulation. Platforms based on PLGA, chitosan, hyaluronic acid, and ionizable lipids degrade into non-toxic, metabolizable products, offering a markedly superior safety profile compared to inorganic nanoparticles, including iron oxide, carbon nanotubes, and semiconductor nanocrystals, which can induce chronic oxidative stress, tissue inflammation, and genotoxicity upon accumulation [107]. Current guidelines from regulatory agencies (FDA, EMA) mandate exhaustive characterization of the nanoparticulate safety profile, including hemocompatibility, genotoxicity, immunotoxicity, and long-term tissue distribution studies, as obligatory requirements for clinical approval, underscoring the importance of integrating safety considerations from the earliest stages of molecular DDS design [108].

## 4. Major Drug Delivery Platforms in Breast Cancer

The advances achieved in understanding tumor biology and the delivery barriers specific to breast cancer have driven the development of a broad and diverse spectrum of nanoparticulate platforms, each offering distinct advantages in terms of loading capacity, pharmacokinetic profile, functionalization potential, and biological compatibility [109]. The current classification of these platforms reflects both the chemical nature of their constituent materials, lipids, synthetic or natural polymers, inorganic materials, and biological structures, and the physicochemical principles governing their assembly, stability, and in vivo behavior [110]. Each platform category covers a distinct therapeutic space: lipid-based systems excel in biocompatibility and clinical validation; polymeric systems offer structural versatility and precise release control; inorganic nanocarriers and organic–inorganic hybrids introduce additional imaging and external stimulation functionalities; and biomimetic systems transfer the biological properties of native cellular components to structures with controlled delivery capacity [111].

### 4.1. Lipid-Based Drug Delivery Systems

Lipid-based systems represent the class with the longest clinical history and the most robust translational validation profile among all DDS categories employed in breast oncology. Their fundamental advantage resides in excellent biocompatibility, intrinsic biodegradability, and capacity to mimic components of natural biological membranes [112,113]. The progression of these systems, from early simple liposomal formulations to current third-generation platforms, including ligand-functionalized liposomes, optimized solid lipid nanoparticles, and nanostructured lipid carriers with multi-stimulus controlled release, illustrates how a fundamental molecular architecture can be iteratively adapted to address the increasingly complex challenges of breast cancer tumor biology [113,114].

#### 4.1.1. Liposomes

Liposomes represent the oldest and most thoroughly characterized nanoparticulate platform in the oncological nanomedicine arsenal, with a clinical history of approximately three decades and several regulatory approvals [115].

PEGylated liposomal doxorubicin (PLD; Doxil^®^), approved by the FDA in 1995, was the first nanomedicine to reach clinical use and established the foundational pharmacological principles of modern oncological nanomedicine: prolonged systemic circulation through reduced opsonization and macrophage clearance, preferential tumor accumulation via the EPR effect, and reduced cardiotoxicity compared with conventional doxorubicin [115]. The polyethylene glycol (PEG) layer grafted onto the liposomal surface through the “stealth” strategy creates a steric hydrophilic coating that impedes the adsorption of opsonic proteins, markedly prolonging the plasma half-life of doxorubicin [115]. Importantly, the regulatory status of liposomal anthracyclines in breast cancer differs between jurisdictions (Table 1): in the United States, Doxil^®^ is not approved for breast cancer, whereas in the European Union the same formulation (Caelyx^®^) is approved as monotherapy for metastatic breast cancer in patients with increased cardiac risk. The second major clinical formulation, non-PEGylated liposomal doxorubicin (Myocet^®^), is approved in the European Union, but not in the United States, for the first-line treatment of metastatic breast cancer in combination with cyclophosphamide, based on a randomized phase III trial demonstrating preserved antitumor efficacy and reduced cardiotoxicity compared with conventional doxorubicin plus cyclophosphamide [116]. Because Caelyx^®^ and Myocet^®^ differ in formulation, pharmacokinetics, and approved indications, they should not be regarded as interchangeable.

The second generation of functionalized liposomes integrates active targeting strategies through conjugation of molecular ligands, such as anti-HER2 antibodies, folate, transferrin, and aptamers, to the liposomal membrane surface, conferring receptor recognition specificity in addition to passive intratumoral accumulation [113]. However, clinical evidence for ligand-targeted liposomes in breast cancer remains limited. The HER2-targeted antibody–liposomal doxorubicin conjugate MM-302 was evaluated only in a non-comparative phase I study (Section 5.1.1), and the subsequent randomized phase II HERMIONE trial (NCT02213744) was stopped in 2016 after the data and safety monitoring board concluded that continuation was unlikely to demonstrate benefit over the comparator treatment (Table 1); consequently, no clinical superiority of targeted over non-targeted liposomes has been demonstrated. Thermoresponsive liposomal systems release the pharmacological agent in a controlled manner within zones of localized hyperthermia (40–42 °C), synchronizing drug release with radiofrequency- or focused ultrasound-induced hyperthermia. pH-sensitive liposomes, designed to destabilize at endosomal or tumor extracellular acidic pH, have been proposed to promote cytosolic release of doxorubicin and to reduce its susceptibility to efflux-mediated resistance, although the extent of endosomal escape and efflux avoidance must be demonstrated experimentally for each formulation.

The major limitations of the classical liposomal platform include long-term physical instability with a tendency toward aggregation and loss of hydrophilic therapeutic agent, high production costs at industrial scale, and uncertain superiority over conventional chemotherapy in terms of overall survival, although improvement of the toxicity profile is well documented [115,116].

#### 4.1.2. Solid Lipid Nanoparticles (SLNs)

Solid lipid nanoparticles (SLNs) were developed in the early 1990s as an alternative to liposomal platforms, offering a distinctive combination of biocompatibility with significant technological advantages: reduced production costs, satisfactory industrial scalability via high-pressure homogenization, superior physicochemical stability compared to liposomes, and the capacity for efficient sterilization, features that have positioned them as a platform of clinical interest in breast oncology [140]. Structurally, SLNs are spherical nanoparticles with diameters ranging from 50 to 1000 nm, comprising a lipid core that is solid at body temperature, composed of triglycerides, waxes, fatty acids, or partial glycerides, stabilized by a surfactant coating that prevents aggregation and ensures dispersibility in aqueous media. Unlike liposomes, which possess a distinct internal aqueous compartment, SLNs incorporate the drug directly into the solid lipid matrix, either in molecular solution or in an amorphous or crystalline state, depending on the degree of ordering of the lipid crystalline lattice [140].

The fundamental structural limitation of SLNs resides in their relatively low drug loading capacity, determined by the highly ordered crystalline organization of the solid lipid matrix, which leaves limited space available for drug molecule insertion without destabilizing the crystalline lattice [140,141]. The tendency toward progressive drug expulsion following lipid polymorphic transitions, with modification of crystalline organization during storage, can lead to formulation instability and variability in the long-term release profile, necessitating advanced formulation solutions or strict storage conditions. These limitations constituted the principal motivation for the development of the next generation of lipid-based systems—nanostructured lipid carriers [141].

#### 4.1.3. Nanostructured Lipid Carriers (NLCs)

Nanostructured lipid carriers (NLCs) represent the second generation of solid lipid nanoparticles, developed in the late 1990s through the deliberate introduction of a liquid-state lipid into the solid lipid matrix of SLNs, with the explicit aim of overcoming the loading capacity and stability limitations of their predecessors [141]. The fundamental structural principle of NLCs resides in the intentional creation of an imperfect and disordered lipid matrix at the nanometric scale. The mixture of solid and liquid lipids in variable ratios, typically 70:30 to 99.9:0.1, generates a crystalline network with multiple imperfections and broad intermolecular spaces, within which drug molecules can be accommodated in significantly greater quantities compared to the perfect crystalline matrix of classical SLNs [141].

This structural innovation translates into concrete and clinically relevant pharmaceutical advantages for breast oncology. The drug loading capacity of NLCs is 2- to 5-fold higher than that of SLNs of comparable lipid composition, enabling attainment of the intratumoral therapeutic concentrations necessary for cytotoxic effect with reduced administration volumes [113]. The physicochemical stability of NLC formulations under ambient storage conditions is markedly superior to both liposomes, prone to unsaturated lipid oxidation and vesicular fusion, and SLNs, susceptible to polymorphic transitions and drug expulsion. The biphasic release profile characteristic of NLCs, featuring an initial rapid release from the liquid lipid phase, followed by slow and prolonged release from the solid lipid matrix, allows both rapid achievement of therapeutic concentration and its sustained maintenance over extended periods, providing an optimal kinetic profile for administration.

From the perspective of applicability across molecular subtypes of breast cancer, NLCs have demonstrated significant preclinical efficacy in the delivery of taxanes (paclitaxel, docetaxel), substantially improving solubility, plasma stability, and therapeutic index compared to conventional clinical formulations such as Cremophor EL or nab-paclitaxel [142]. Surface functionalization of NLCs with targeting ligands, including folic acid for FOLR1 receptors overexpressed in TNBC, and anti-CD44 antibodies for tumor stem cells, has demonstrated significantly improved cellular specificity and antitumoral efficacy in in vitro and in vivo models of triple-negative breast cancer. The compatibility of NLCs with large-scale industrial production techniques through high-pressure homogenization or the hot emulsion method, without the requirement for toxic organic solvents, constitutes a major advantage from the perspective of clinical translation [142].

### 4.2. Polymeric DDS

Polymeric systems represent the most diverse and versatile category of nanoparticulate platforms employed in breast oncology, offering a degree of structural and functional control superior to that of classical lipid-based platforms. The chemical nature of the polymer, molecular weight, degree of crosslinking, supramolecular architecture, and type of functionalization can be independently modulated to obtain release profiles, biodistribution, and targeting specificity adapted to the biological requirements of each tumor subtype [143]. The polymers employed in these systems fall within two major categories: synthetic biodegradable polymers, such as PLGA, polycaprolactone, and polyaminoesters, and natural polymers, including chitosan, hyaluronic acid, and albumin, each with distinct advantages and limitations in the context of oncological applications. The chemical versatility of polymeric platforms additionally permits the native integration of stimuli-responsive functionalities, multivalent co-delivery systems, and active targeting ligands without compromising the structural integrity of the nanoparticulate vector [144].

#### 4.2.1. Polymeric Nanoparticles

Polymeric nanoparticles represent the most extensively studied subcategory of polymeric delivery systems, with a positive translational balance reflected by multiple clinically approved formulations or those in advanced-phase clinical trials [145].

Poly(lactic-co-glycolic acid) (PLGA) represents the reference polymer in nanomedicine oncology, uniquely approved by both FDA and EMA for parenteral and oral administration, benefiting from an extensively documented biodegradability and biocompatibility profile. Hydrolysis of PLGA generates lactic acid and glycolic acid, endogenous metabolites of the Krebs cycle, eliminated through normal physiological pathways without long-term tissue accumulation [146]. Precise control of the lactic acid:glycolic acid ratio and of PLGA molecular weight allows modulation of the degradation duration from several weeks to several months, with direct implications for the sustained release profile of the therapeutic agent. PLGA nanoparticles loaded with paclitaxel or docetaxel have demonstrated superior antitumoral efficacy compared to conventional formulations in preclinical breast cancer models, with a significantly improved toxicity profile through reduction in systemic exposure to peak concentrations [146]. Surface functionalization of PLGA with trastuzumab, the anti-HER2 monoclonal antibody, has generated nanoparticulate systems with high targeting specificity for HER2^+^ cells and a biphasic release profile (initial rapid release followed by sustained release over 14 days) [147].

Human serum albumin nanoparticles, exemplified clinically by nab-paclitaxel (Abraxane^®^), which is an FDA-approved formulation for metastatic breast cancer, exploit the endogenous trans-endothelial albumin transport pathways mediated by SPARC receptors overexpressed in the tumor stroma, thereby circumventing the need for toxic solvents (Cremophor EL) and significantly improving the tolerability profile compared to conventional paclitaxel [147]. The principal limitations of polymeric nanoparticles include the potential for immunological activation upon repeated administration, variability in batch-to-batch reproducibility in industrial-scale production, and the complexity of functionalization processes, which can compromise the colloidal stability of the final formulation [148].

#### 4.2.2. Polymeric Micelles

Polymeric micelles represent a distinctive subcategory of polymeric systems, formed through the spontaneous self-assembly of amphiphilic copolymers in aqueous media at concentrations above the critical micelle concentration (CMC) [149]. The architecture of polymeric micelles is characterized by a hydrophobic core, which selectively encapsulates lipophilic drugs of limited solubility, and an outer hydrophilic shell ensuring colloidal stability, evasion of macrophage clearance, and solubilization of the system in physiological media. The dimensions of polymeric micelles are typically below 100 nm (5–100 nm), smaller than solid polymeric nanoparticles, conferring deep intratumoral penetration capacity and a reduced tendency for sequestration within the perivascular layers of the dense ECM [149].

**Figure 4:**
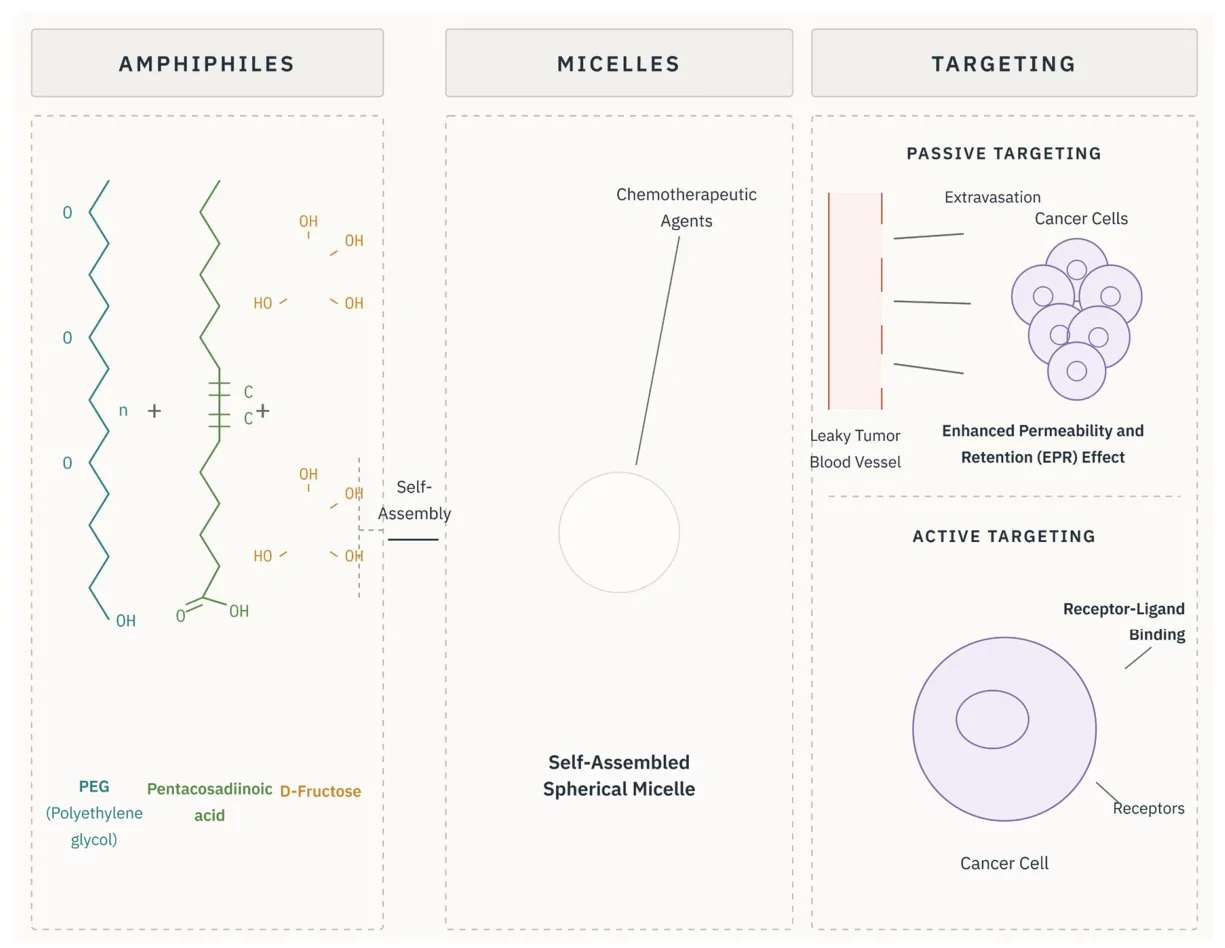
Scheme for the preparation of the novel micelles and their passive and active targeting. Adapted from [151].

The most frequently employed micellar systems in breast oncology are PEG-PLGA, PEG-PCL (polycaprolactone), and PEG-PBAE (poly(β-amino ester)) block copolymers, in which the hydrophilic PEG block provides the external anti-opsonizing shell and the hydrophobic block forms the drug encapsulation core [150]. A significant clinical advantage of polymeric micelles (Figure 4) resides in the elimination of toxic excipients from conventional taxane formulations, Cremophor EL for paclitaxel and Tween 80 for docetaxel, responsible for severe hypersensitivity reactions. Genexol-PM^®^, a Cremophor-free paclitaxel formulation based on poly(ethylene glycol)–poly(D,L-lactide) (PEG-PDLLA) micelles, was approved in South Korea in 2007 for breast, lung, and ovarian cancers but has not been approved by the FDA; in a randomized phase III trial in metastatic breast cancer, it showed non-inferior efficacy compared with conventional paclitaxel (Table 1).

Stimuli-responsive polymeric micelles, designed to lose their structural integrity at the acidic tumor pH or in the presence of intracellular glutathione, add a further level of selectivity. Micelles composed of mPEG-PBAE copolymers progressively destabilize at pH 6.5–5.5, releasing the co-loaded agent (paclitaxel and triptolide) selectively within the acidic tumor microenvironment, with demonstrated synergistic effects in in vitro models [151]. The principal limitations of polymeric micelles are their limited stability under conditions of systemic dilution; the CMC may be exceeded in the circulation, leading to premature micelle dissociation and uncontrolled drug release, and their relatively low loading capacity compared to matrix nanoparticles [128].

#### 4.2.3. Dendrimers

Dendrimers represent an entirely distinct category of polymeric systems, characterized by a highly branched three-dimensional architecture with a regular, monodisperse structure, in contrast to the linear or crosslinked polymers of nanoparticles and micelles, obtained through controlled repetitive syntheses that generate successive branching sequences [152]. The star-spherical morphology of dendrimers, with a cavernous internal structure and an extremely high density of surface functional groups, confers upon them distinct transport and functionalization properties. Drugs can be incorporated either through physical encapsulation within internal cavities, specific for hydrophobic molecules, or through covalent conjugation to terminal surface groups specific for prodrugs and targeting ligands.

The most thoroughly characterized dendrimer employed in breast oncology is PAMAM (polyamidoamine), available in generations G1–G10, with dimensions increasing from ~1 nm (G1) to ~14 nm (G10) and an exponentially increasing number of terminal amine groups at the surface [153]. The high density of positive charges on PAMAM dendrimers permits strong electrostatic interactions with nucleic acids, plasmid DNA, siRNA, and miRNA, generating stable dendriplexes with potential in gene therapeutic delivery. Conjugation of PAMAM dendrimers with trastuzumab and docetaxel or paclitaxel has generated systems with high HER2^+^ targeting specificity, with predominantly preferential internalization in SKBR-3 (HER2^+^) cells compared to MCF-7 (HER2^−^) cells, demonstrating the concept of combined multivalent drug-antibody delivery [153].

The principal limitations of dendrimers in the context of clinical translation reside in the intrinsic cytotoxicity of higher generations of unmodified PAMAM, determined by the density of positive surface charges, which disrupt cellular and erythrocyte membranes, and in the synthetic complexity generating high production costs and standardization difficulties [154].

### 4.3. Inorganic Nanocarriers and Organic–Inorganic Hybrid Systems

Inorganic nanocarriers and organic–inorganic hybrid systems represent a distinct and rapidly expanding category of the oncological nanomedicine arsenal, introducing unique therapeutic and diagnostic functionalities inaccessible to purely organic platforms. Photothermal conversion capacity, superparamagnetic behavior, luminescence, and controllable radiopacity constitute properties exploitable both in therapy and in integrated multimodal imaging [155]. The defining characteristic of this category resides in the capacity to combine multiple therapeutic modalities: chemotherapy, photothermal therapy, photodynamic therapy, and magnetic hyperthermia, within a single nanoparticulate platform, generating synergistic effects superior to monotherapy and opening the path toward integrated theranostic approaches [156,157]. The principal limitation common to all unmodified inorganic nanocarriers is slow clearance and the potential for long-term tissue accumulation, which has driven their hybridization with biocompatible organic coatings as a strategy for reconciling inorganic functionality with organic biocompatibility [157].

#### 4.3.1. Gold, Mesoporous Silica, and Iron Oxide Nanoparticles

Gold nanoparticles (AuNPs) occupy a central position in oncological nanomedicine by virtue of their remarkable physical properties and highly versatile surface chemistry. The most therapeutically exploited property is localized surface plasmon resonance (LSPR), the collective oscillation of conduction electrons under light irradiation, which generates highly efficient optical absorption and converts photonic energy into localized heat with efficiency superior to any known organic system [135]. The morphology of AuNPs (nanospheres, nanorods, nanostars) modulates the LSPR absorption wavelength: spherical nanoparticles absorb in the visible range (520–530 nm), while gold nanorods (GNRs) exhibit absorption in the near-infrared (NIR, 650–950 nm), the biological window of maximum tissue penetration, essential for photothermal therapy (PTT) applications [158]. NIR-irradiated AuNP-based photothermal therapy generates local temperatures of 42–55 °C within the tumor, producing protein denaturation, mitochondrial dysfunction, and cell death through apoptosis or necrosis, with increased selectivity relative to normal tissues that do not accumulate the nanoparticles. Conjugation of gold nanorods with cytotoxic drugs, including doxorubicin and methotrexate, and active targeting ligands (MUC1 aptamers, anti-HER2 antibodies, folic acid) has generated combined chemo-photothermal theranostic platforms with demonstrated synergistic antitumoral efficacy in HR^+^ and TNBC breast cancer models [158].

Mesoporous silica nanoparticles (MSNs) constitute a distinct inorganic category, valued not for optical or magnetic properties but for exceptional drug loading capacity, conferred by high specific surface area (500–1200 m^2^/g), controllable pore volume (0.5–2.5 cm^3^/g), and pore size (2–50 nm), which permit the simultaneous incorporation of multiple therapeutic agents [159]. Versatile chemical modification of the surface, through amine, carboxyl, thiol functionalities, or through grafting of stimuli-responsive polymers, permits the design of systems with pH-, redox-, or enzymatically controlled release, directly applicable to the acidic and oxidative tumor microenvironment [159]. Surface functionalization of MSNs with targeting ligands, such as aptamers or antibodies, has been explored to enhance their recognition and internalization by breast cancer cells in preclinical models [159]. Au-MSN hybrids, with gold nanorods internalized within the mesoporous silica structure, combine the photothermal conversion capacity of gold with the loading capacity of silica, generating synergistic chemo-photothermal platforms with GSH-responsive release [159].

Superparamagnetic iron oxide nanoparticles (SPIONs) exploit magnetic behavior at the nanometric scale: below the critical dimensions of magnetic domains (<20 nm), Fe_3_O_4_ or γ-Fe_2_O_3_ particles exhibit superparamagnetism, magnetization in an external field without remanent magnetization upon field removal, a property essential for two major applications in oncological therapy [160]. The first is magnetic hyperthermia: subjected to a high-frequency alternating magnetic field, SPIONs generate localized heat through Néel and Brown relaxation mechanisms, with potential for selective tumor ablation. The second is magnetic guidance of the delivery system: through the application of an external magnetic field, SPIONs and hybrid nanoparticles incorporating them can be preferentially accumulated at the tumor site, augmenting intratumoral accumulation beyond the limits of the EPR effect. SPIONs simultaneously function as T2 contrast agents for MRI, enabling non-invasive monitoring of the intratumoral distribution of DDS [160].

#### 4.3.2. Organic–Inorganic Hybrid Systems

Organic–inorganic hybrid systems represent a conceptual and material synthesis of the most valuable properties of both categories, the therapeutic and diagnostic functionalities specific to inorganic components combined with the biocompatibility, biodegradability, and functionalization versatility of organic components, generating nanoparticulate platforms with performance profiles inaccessible to either category individually [161]. The principal organic–inorganic hybrid system architectures investigated in breast cancer include lipid–polymer nanoparticles (LPHNPs), nanoparticles with an inorganic core and polymeric or lipidic coating, and inorganic-biomolecular systems.

Hybrid lipid–polymer nanoparticles (LPHNPs) combine a polymeric core, typically PLGA, PCL, or PLA, with an outer lipid coating, simultaneously achieving the advantages of the kinetic release control characteristic of polymeric matrices and the excellent biocompatibility, colloidal stability, and immunological evasion properties of the lipid shell [162,163]. Preclinical studies in MCF-7 and MDA-MB-231 breast cancer models have demonstrated that in preclinical breast cancer models, fucose-anchored LPHNPs co-loaded with methotrexate and aceclofenac showed enhanced cellular uptake in MCF-7 and MDA-MB-231 cells, synergistic antiproliferative effects, improved pharmacokinetic parameters compared with the free drugs, and reduced tumor volume in a chemically induced murine breast tumor model [162]. Co-delivery of chemotherapeutic agents with photothermal agents in LPHNPs, for example, doxorubicin and indocyanine green (ICG) co-assembled in lipid–polymer nanoparticles, has generated chemo-photothermal platforms with NIR-triggered synergistic antitumor efficacy in preclinical models [163].

Systems with an inorganic core and organic coating, AuNPs coated with PLGA or lipids, SPIONs functionalized with PEG and stimuli-responsive polymers, MSNs enveloped in liposomal membranes, combine the optical or magnetic properties of the inorganic core with the capacity of the coating to prolong circulation, reduce nonspecific toxicity, and permit functionalization with molecular targeting ligands. Hybrid systems, based on bilayered lipid nanostructures partially coated with silica, integrate the biomimetic properties of liposomes with the mechanical and chemical stability of silica, overcoming the tendency toward fusion and aggregation of classical liposomes, with promising applications in the simultaneous delivery of imaging agents and cytotoxic compounds in breast cancer. The principal translational challenges of organic–inorganic hybrid systems include synthesis complexity, difficulty in standardizing organic–inorganic interfacial properties, and rigorous evaluation of the biodegradability of the inorganic component under long-term in vivo conditions [158,159,160].

### 4.4. Biomimetic DDS

Biomimetic systems represent the most recent and conceptually innovative category of delivery platforms, based on the principle that the most effective biological navigation strategies are those that mimic or directly utilize structures and mechanisms already optimized by evolution within the human organism [164]. In contrast to synthetic lipidic, polymeric, or inorganic systems, which seek to reproduce specific biological properties through molecular engineering, biomimetic platforms directly exploit functional components of biological origin, including cell membranes, extracellular vesicles, and surface proteins. When integrated as nanoparticle coatings or therapeutic vectors, these components can confer the molecular recognition and immunological navigation capabilities of the source cells to the resulting nanocarriers [165,166]. The fundamental competitive advantage of these platforms resides in their intrinsic capacity for immunological clearance evasion and homotopic targeting. These properties are inaccessible to any synthetic platform, regardless of the degree of functionalization [166].

#### 4.4.1. Cell Membrane-Coated Nanoparticles

Cell membrane-coated nanoparticles (CMCNPs) are hybrid systems combining a synthetic nanoparticulate core, polymeric (PLGA, PCL), lipidic, or inorganic, with an outer coating formed from native cell membranes isolated from diverse cell types, including erythrocytes, leukocytes, macrophages, cytotoxic T lymphocytes, stem cells, and tumor cells [167].

Different cell membrane sources confer distinct functional properties upon CMCNPs in the context of breast cancer. Erythrocyte membranes transfer to the nanoparticle the exceptional immunological evasion character and circulatory longevity of red blood cells (120-day circulatory lifespan), through the transmission of CD47 molecules that inhibit recognition and phagocytosis by splenic macrophages [167,168]. Tumor cell membranes, particularly those from 4T1 (murine breast cancer) and MCF-7 or MDA-MB-231 (human breast cancer) lines, confer upon nanoparticles the property of homotopic targeting. The molecular complementarity between the nanoparticle membrane and tumor cells of the same origin leads to intratumoral accumulation 20-fold higher than that of nanoparticles coated with membranes from other cell types, mediated through galectin-3/carcinoembryonic antigen interactions and other homolog-specific adhesion molecules [168]. The therapeutic applications of CMCNPs in breast cancer demonstrate that systems with a PLGA polymeric core coated with 4T1 cell membranes and loaded with paclitaxel inhibit macrophage phagocytosis and increase intracellular uptake in 4T1 cells, with synergistic antitumoral effects in combined photothermal models [111]. Macrophage membranes transfer inflammatory tropism properties and blood–brain barrier crossing capacity, relevant for therapeutic delivery to brain metastases [169]. Cytotoxic T lymphocyte membranes can be employed to camouflage nanoparticles as immune effectors of the adaptive system, facilitating penetration into sites with high intratumoral lymphocyte density.

#### 4.4.2. Extracellular Vesicles and Exosomes

Extracellular vesicles (EVs), a category encompassing exosomes (30–150 nm), microvesicles (150–1000 nm), and apoptotic bodies (>1000 nm), represent endogenous biological nanocarriers par excellence, naturally secreted by virtually all cell types as mediators of intercellular communication and transfer of bioactive molecules [170]. Exosomes, the subcategory of highest therapeutic interest, are vesicles of endosomal origin enveloped by a lipid bilayer accompanied by a characteristic proteome, such as tetraspanins (CD9, CD63, CD81), heat shock proteins, Alix, and TSG101, which confer upon them biostability, biocompatibility, and the capacity to traverse biological barriers superior to any synthetic platform [170]. Their reduced dimensions (50–150 nm), negative surface charge, lipid composition rich in cholesterol and sphingomyelin, and absence of xeno-antigenic components confer upon them a practically immunologically invisible profile with respect to the host immune system, with prolonged circulation time and broad tissue distribution [170,171].

The capacity of exosomes to encapsulate and deliver diverse categories of therapeutic agents, such as small hydrophobic and hydrophilic molecules, proteins, messenger RNA, siRNA, microRNA, CRISPR-Cas constructs, and plasmid DNA, through the unique combination of lipid membranes permeable at physiological temperature and an internal aqueous compartment, positions them as universal vehicles in oncological nanomedicine [171]. Strategies for loading exosomes with therapeutic agents are divided into exogenous methods, including electroporation, sonication, saponin permeabilization, and passive incubation, and endogenous methods, including transfection of the source cell to produce pre-loaded exosomes through natural biogenesis [171,172].

Surface functionalization of exosomes with molecular targeting ligands, such as iRGD peptides, anti-HER2 aptamers, and anti-folate antibodies, augments delivery specificity beyond the natural tropism of the source cell, generating systems with two complementary levels of selectivity: biological, through membrane composition, and molecular, through conjugated ligands [172]. A particular advantage in breast cancer is the capacity of exosomes to traverse the blood–brain barrier, which is relevant for the treatment of brain metastases, frequent in the HER2^+^ and TNBC subtypes. This is a property inaccessible to conventional synthetic nanoparticles of comparable dimensions. The main characteristics, advantages, limitations, and clinical status of the drug delivery platforms discussed in this section are summarized in Table 2.

## 5. Breast Cancer-Specific Targeting Strategies

Molecular targeting strategies represent the most individualized and most subtly adapted dimension of nanomedicine applied to breast cancer, translating knowledge of the receptor-specific profile of each tumor subtype into delivery solutions with markedly increased selectivity compared to conventional platforms [173]. Unlike general design principles, such as size, charge, and stimuli-responsiveness, which define the physicochemical behavior of the nanoparticulate vehicle, targeting strategies operate at the molecular interface between the nanoparticle and the target cell, exploiting the overexpression or qualitative modification of receptors, surface markers, or tumor microenvironment characteristics to direct the therapeutic agent with precision. This approach is particularly important in breast cancer given that the molecular heterogeneity of the disease renders a universal targeting strategy impossible and necessitates individualization of therapeutic vectors in accordance with the tumor biology of each patient [173].

### 5.1. Receptor-Mediated Targeting

Receptor-mediated targeting aims to enhance the recognition and internalization of nanocarriers by tumor cells expressing specific surface molecules. However, potential targets differ markedly in cellular localization, expression in normal tissues, and level of clinical validation [174]. A distinction should therefore be made between clinically validated therapeutic targets, such as HER2, which has been successfully exploited by monoclonal antibodies and antibody–drug conjugates, and experimental delivery targets, such as the folate receptor alpha (FOLR1), the transferrin receptor, and CD44, whose value as nanocarrier targets has so far been demonstrated predominantly in preclinical studies. Estrogen and progesterone receptors, although clinically validated targets of endocrine therapy, are predominantly intracellular nuclear receptors and are therefore not conventional surface targets for receptor-mediated nanocarrier internalization.

#### 5.1.1. The HER2 Receptor

The HER2 receptor represents the most extensively exploited surface target in breast oncology. HER2 overexpression or ERBB2 amplification occurs in approximately 13–15% of breast cancers [6,42], and the markedly higher receptor density on HER2-positive tumor cells compared with normal epithelial cells makes HER2 an attractive target for drug delivery systems, although its low-level expression in normal tissues, including cardiomyocytes, must be considered with respect to off-target toxicity [42]. Trastuzumab, the humanized anti-HER2 monoclonal antibody approved by both the FDA and EMA, has constituted the prototype targeting ligand for the second generation of oncological nanomedicines. Its conjugation to the surface of liposomes and other nanocarriers has been shown in preclinical models of HER2-overexpressing breast cancer to enhance receptor-mediated internalization and antitumor efficacy; however, antibody targeting did not increase overall tumor accumulation compared with non-targeted counterparts (Section 3.1) [83,84]. The clinical translation of HER2-targeted nanocarriers has so far been limited: MM-302, a HER2-targeted antibody–liposomal doxorubicin conjugate, was evaluated in a non-comparative phase I study in patients with advanced HER2-positive breast cancer [125], and no clinical evidence of superiority over non-targeted liposomal formulations is currently available. By contrast, antibody–drug conjugates, notably trastuzumab deruxtecan (T-DXd), have validated the principle that vectorization of a potent cytotoxic payload via an anti-HER2 antibody can generate remarkable antitumor efficacy, even in tumors with low HER2 expression, significantly extending the spectrum of patients eligible for HER2-directed therapy [42].

#### 5.1.2. Estrogen and Progesterone Receptors

Estrogen and progesterone receptors are expressed in approximately 70–80% of breast cancers and represent clinically validated targets of endocrine therapy [6,40]. From a delivery perspective, however, ER and PR are predominantly intracellular, nuclear, ligand-activated transcription factors, as reflected by their nuclear immunohistochemical staining in routine diagnostics [40], rather than surface receptors capable of mediating classical receptor-mediated endocytosis. Although membrane-initiated estrogen signaling, including that mediated by the G-protein-coupled estrogen receptor (GPER), has been described [175], its contribution to nanocarrier internalization has not been clearly established. Consequently, nanocarriers functionalized with estrogen receptor ligands, such as estradiol, estrone, or tamoxifen [174], should be regarded as experimental systems, and their enhanced uptake in ER-positive cells cannot be automatically attributed to receptor-mediated endocytosis without direct mechanistic evidence. Nanocarriers co-delivering endocrine agents, such as tamoxifen, with cytotoxic drugs represent a distinct, pharmacologically motivated strategy aimed at simultaneously targeting estrogen signaling and tumor cell proliferation, which likewise remains at the preclinical stage.

#### 5.1.3. Folate, Transferrin, and CD44 Receptors

The alpha-type folate receptor (FOLR1) is overexpressed in a subset of breast cancers, particularly TNBC, whereas its expression in normal tissues is restricted mainly to the apical surface of polarized epithelia, such as the renal proximal tubules, lung, and choroid plexus, which makes it an attractive, although not tumor-exclusive, experimental delivery target [176]. Conjugation of folic acid to the surface of PLGA nanoparticles, polymeric micelles, or liposomes enables specific recognition of the FOLR1 receptor and receptor-mediated endocytic internalization of the cytotoxic agent, with significant reduction in exposure in normal tissues. The transferrin receptor (TfR, CD71) is overexpressed in tumor cells with elevated proliferative activity due to the substantial iron requirement for DNA synthesis, and has been investigated as an experimental target for the delivery of nanoparticles loaded with cisplatin, doxorubicin, or siRNA in TNBC and HER2^+^ cell lines; however, TfR1 is also highly expressed in normal proliferating cells, erythroid precursors, hepatocytes, and the endothelium of the blood–brain barrier, which must be considered with respect to off-target delivery [176]. CD44, the transmembrane glycoprotein implicated in cell adhesion, migration, and intracellular signaling, is overexpressed both in differentiated TNBC cells and in breast cancer stem cells (CD44^+^/CD24^−^/low), conferring a dual role as a marker of aggressiveness and as a therapeutic target. Hyaluronic acid (HA) nanoparticles or HA-functionalized nanoparticles exploit the specific interaction between HA and CD44 for preferential internalization in CD44^+^ cells compared to CD44^−^/low cells, with antitumor efficacy demonstrated in preclinical in vitro and in vivo models of TNBC and HR^+^ breast cancer, although CD44 is also expressed on hematopoietic, epithelial, and stromal cells [176].

### 5.2. Targeting via Peptides, Antibodies, and Aptamers

Beyond small molecular ligands, including folic acid, transferrin, and hyaluronic acid, a superior category of molecular specificity is conferred by recognition peptides, monoclonal antibodies, and aptamers: biomolecules with epitope-specific recognition capacity and conjugation versatility applicable to diverse nanoparticulate platforms [176]. Targeting peptides, particularly RGD (Arg-Gly-Asp) and iRGD (a cyclic RGD analog with tumor-penetrating properties), bind with high affinity to integrins αvβ3 and αvβ5, overexpressed on the tumor vascular endothelium and on invasive breast carcinoma cells, mediating both selective vascular accumulation and active penetration into the deep tumor parenchyma through NRP-1-dependent transcytosis mechanisms [177]. Aptamers are oligonucleotides with three-dimensional structures capable of recognizing molecular targets with antibody-comparable specificity, with advantages of chemical stability, minimal immunogenicity, and reduced synthesis cost. Moreover, they have shown promising results in preclinical breast cancer targeting: the AS1411 aptamer (anti-nucleolin), the anti-MUC1 aptamer, and the sTN58 aptamer (anti-CD44) achieve selective recognition of breast tumor cells and chemoresistant tumor stem cells, facilitating the internalization of functionalized nanoparticles while simultaneously inhibiting signaling pathways implicated in tumor aggressiveness. Nanobodies, which are variable domain fragments of camelid antibodies with dimensions of 2–4 nm, combine antibody specificity with ultra-small dimensions compatible with deep matrix penetration, representing an emerging direction for addressing the physical barriers of desmoplastic breast cancer [177]. It should be noted, however, that although these ligands have shown promising results in preclinical breast cancer models, none has yet been clinically validated as a nanocarrier-targeting moiety in breast cancer [177].

### 5.3. Tumor Microenvironment Targeting

The tumor microenvironment, with its distinctive biochemical characteristics, hypoxia, extracellular acidity, overexpression of matrix metalloproteinases and hyaluronidase, and elevated intracellular glutathione concentrations, constitutes not only a delivery barrier but also an opportunity for the active targeting of non-neoplastic stromal components [178]. Cancer-associated fibroblasts (CAFs), the principal producers of desmoplastic ECM and pro-tumoral factors, can be targeted by nanoparticles functionalized with ligands of the fibroblast activation protein receptor (FAP) or with TGF-β inhibitors, partially normalizing the stroma and improving the access of therapeutic vehicles into the tumor depth. Tumor-associated macrophages polarized toward the immunosuppressive M2 phenotype constitute another valuable stromal target: nanoparticles that repolarize M2 macrophages toward the pro-inflammatory M1 phenotype restore local anti-tumor immunity and transform immunologically “cold” tumors into “hot” tumors susceptible to immunotherapy response [179]. Tumor vasculature can be targeted for the selective delivery of anti-angiogenic or photothermal agents, with potential for vascular normalization and secondary improvement of conventional DDS penetration. Dual nanoparticulate systems combining stromal targeting with anti-neoplastic drug delivery, such as nanoparticles co-delivering ECM-degrading enzymes and cytotoxics, represent the most advanced expression of this integrated strategy [179].

### 5.4. Targeted Delivery to Tumor Stem Cells

Breast cancer stem cells (BCSCs), identified by the CD44^+^/CD24^−^/low phenotype and by elevated expression of aldehyde dehydrogenase (ALDH1), represent the cellular subpopulation with the highest potential for relapse, metastasis, and resistance to conventional therapy, constituting, accordingly, a priority target of next-generation nanomedicine strategies. BCSCs are positively selected by standard chemotherapy, which preferentially eliminates differentiated, proliferative tumor cells, maintaining a residual quiescent population with elevated activity of ABC efflux pumps and DNA repair mechanisms, underlying post-treatment relapse [180]. Hyaluronic acid nanoparticles or HA-functionalized nanoparticles exploit CD44 overexpression on BCSCs for preferential internalization into this subpopulation, releasing agents that inhibit Notch, Wnt/β-catenin, and Hedgehog signaling pathways, which are implicated in the maintenance of the stem phenotype, or ALDH inhibitors [180,181]. Dual nanoparticulate systems combining anti-proliferative chemotherapy with differentiating agents or stem niche inhibitors simultaneously address the proliferative tumor mass and the residual stem subpopulation, reducing the probability of resistant clonal selection and post-treatment relapse. The sTN58 anti-CD44 aptamer, recently characterized in chemoresistant TNBC models, demonstrates that highly selective molecular recognition of BCSCs can be achieved through bioreceptors with superior specificity compared to conventional polysaccharide ligands, while simultaneously directly inhibiting cellular invasiveness [181].

### 5.5. Targeting of Metastatic Niches (Bone, Brain, Lung)

**Figure 5:**
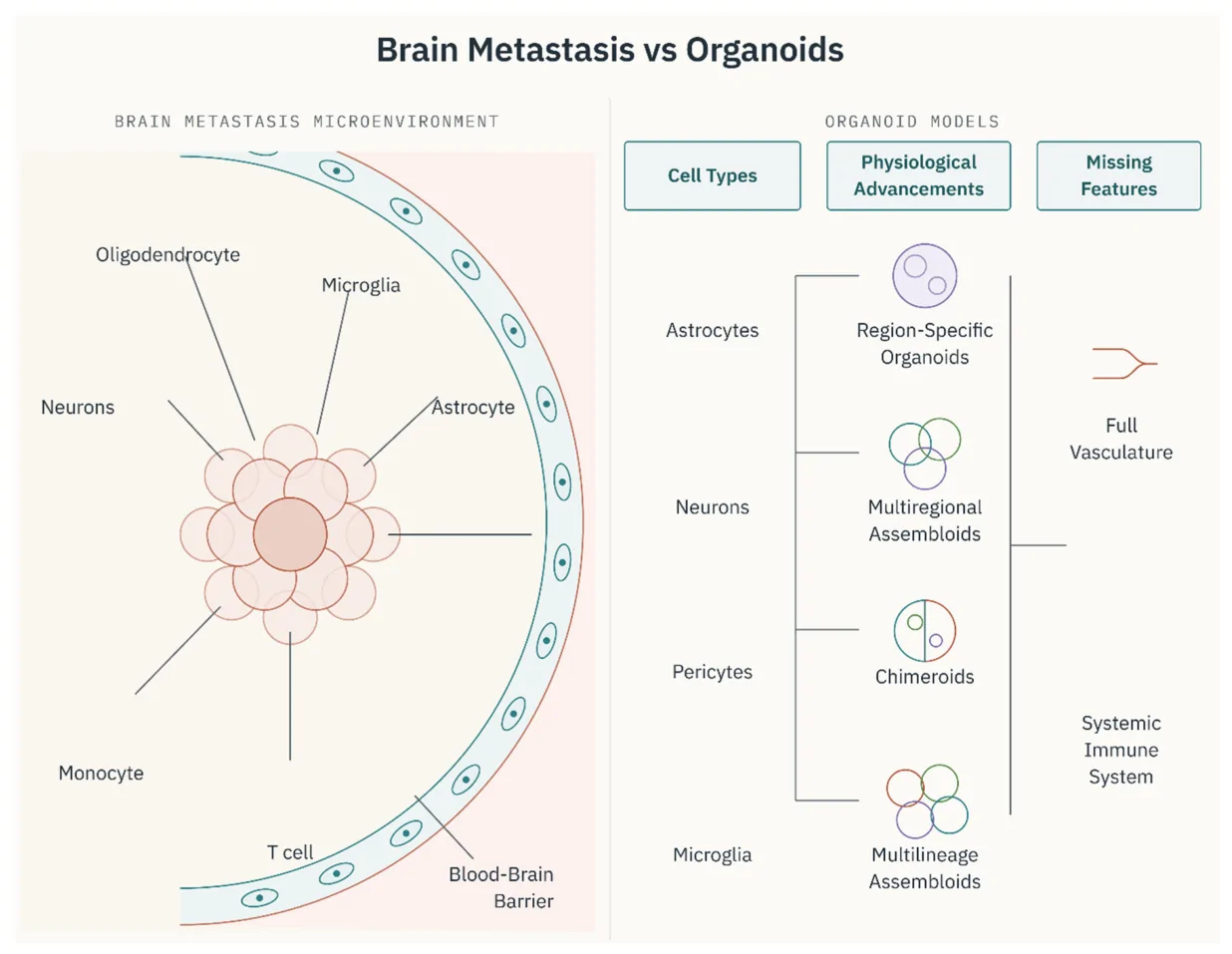
Comparison of the metastatic brain cellular microenvironment and brain organoid models. Adapted from [160].

Distant metastases, particularly at the osseous, cerebral, and pulmonary levels, represent the most severe evolutionary stage of breast cancer and the most difficult to treat, as each metastatic niche presents a distinct local biology that necessitates individualized targeting strategies radically different from those optimized for the primary breast tumor [182]. Bone metastases, frequent in the HR^+^ subtype (50–70% of cases), exploit the “vicious cycle” mechanism between tumor cells and activated osteoclasts: bone-avid nanoparticles functionalized with alendronic acid, zoledronic acid, or hydroxyapatite-affinity peptides achieve preferential concentration in the bone niche and simultaneously deliver anti-resorptive agents and cytotoxics, interrupting the vicious cycle of osteolysis and tumor proliferation. Brain metastases (Figure 5), particularly frequent in the HER2^+^ subtype (20–40%) and TNBC (30–46%), necessitate traversal of the blood–brain barrier (BBB), one of the most selective biological barriers in the organism, through a range of strategies, including receptor-mediated transcytosis (transferrin, low-density lipoproteins, insulin), the use of exosomes with natural BBB tropism, and nanoparticulate systems with active charge reversal in the cerebral microenvironment [182].

Nanoparticles functionalized with peptides that bind the LRP1 receptor (Low-Density Lipoprotein Receptor-related Protein 1), overexpressed on the BBB endothelium, have been investigated for transcytosis and cerebral intratumoral concentration [183]; however, considering the findings of Khan et al., LRP1-mediated delivery should be regarded as formulation-, model-, and lesion-dependent rather than an established efficient strategy, as LRP1-targeted peptide distribution was found to be poor in the JIMT1-BR and 231-BR breast cancer brain metastasis models [184].

## 6. Cargo-Specific Delivery Approaches for Therapeutic Agents

The biological diversity of breast cancer, expressed through the multiplicity of molecular subtypes, resistance mechanisms, and available therapeutic targets, has driven the development of an equally diverse spectrum of therapeutic cargo delivery strategies, with each category of active agent imposing distinct requirements in terms of formulation, chemical protection, controlled release, and intracellular compartment specificity [185]. From the small hydrophobic molecules of classical cytotoxic chemotherapy, to the hormonal agents and molecular inhibitors of targeted therapy, to the fragile, negatively charged nucleic acids of gene therapy, and to therapeutic proteins and peptides—each category requires loading solutions adapted to its physicochemical properties, mechanism of action, and the biological compartment in which the therapeutic effect must be exerted. Understanding these specific requirements and rationally designing systems capable of simultaneously satisfying them constitutes the core of cargo-specific delivery approaches, transforming the nanoparticulate carriers from a simple passive mechanism into an active, adaptable, and intelligent system [185].

### 6.1. Delivery of Chemotherapeutic Drugs

Cytotoxic chemotherapeutic drugs, including anthracyclines (doxorubicin, epirubicin), taxanes (paclitaxel, docetaxel), platinum compounds (cisplatin, carboplatin), and vinca alkaloids, represent the most extensively studied chapter in oncological nanomedicine, by virtue of their well-characterized pharmacological profile and the significant systemic toxicity that necessitates their encapsulation within delivery systems [186]. The principal challenges associated with this category derive from the frequently unfavorable physicochemical properties of cytotoxic compounds: extreme hydrophobicity; paclitaxel and docetaxel require toxic solvents (Cremophor EL, Tween 80) in conventional formulations; reduced chemical stability; short plasma half-life; and an inadequate volume of distribution. Solid lipid nanoparticles and NLCs have demonstrated superior taxane incorporation capacity compared to liposomes, with significant improvement of the bioavailability profile and reduction in peripheral neurotoxicity compared to Cremophor EL-based formulations [186]. Albumin-bound paclitaxel (nab-paclitaxel, Abraxane^®^), approved by the FDA and EMA for metastatic breast cancer, and the micellar paclitaxel formulation Genexol-PM^®^, approved in South Korea, represent clinically established examples of this concept, with Genexol-PM^®^ showing non-inferior efficacy compared with conventional paclitaxel in a phase III trial in metastatic breast cancer [124] (Table 1). Doxorubicin, the prototypic anthracycline, has generated several clinically approved liposomal formulations (Doxil^®^/Caelyx^®^ and Myocet^®^), which differ in formulation, regulatory status, and approved indications (Table 1) and which have shown that nanometric encapsulation can improve the cardiac safety profile of a high-potency cytotoxic agent. Stimuli-responsive nanoparticulate systems for chemotherapeutic delivery, with pH-dependent release in the tumor endosome, redox-dependent release in the presence of intracellular GSH, or photoactivated release via NIR irradiation, may help to overcome pharmacokinetic limitations and, in selected preclinical models, efflux-mediated resistance, although the extent of cytosolic delivery and efflux avoidance must be demonstrated experimentally for each system [93].

### 6.2. Delivery Systems for Hormonal Therapy

Hormonal therapy, employing aromatase inhibitors (anastrozole, letrozole, exemestane), selective estrogen receptor modulators (tamoxifen, raloxifene), and selective estrogen receptor degraders (fulvestrant), remains the central therapeutic pillar of HR^+^ breast cancer, conferring significant long-term survival benefits [8,15]. The pharmacokinetic limitations of these agents are, however, compound-specific and should not be generalized. Tamoxifen is well absorbed after oral administration but acts largely as a prodrug, requiring CYP2D6-mediated bioactivation to its active metabolite endoxifen; interindividual variability in CYP2D6 activity, rather than poor oral bioavailability, therefore influences exposure to its active metabolite [187]. Letrozole, in contrast, has nearly complete oral bioavailability [188], whereas fulvestrant has low oral bioavailability owing to poor absorption and presystemic metabolism and is therefore administered as a long-acting intramuscular injection [189]. In addition, the emergence of resistance to endocrine therapy through activation of the PI3K/AKT/mTOR and CDK4/6 pathways limits long-term clinical efficacy [15]. Accordingly, the rationale for nanoformulating endocrine agents differs between compounds: rather than overcoming poor oral bioavailability, which is not a limitation for most of these drugs, nanocarriers have been investigated mainly to modify release kinetics and tissue distribution, to enable local or alternative routes of administration, or to co-deliver endocrine agents with inhibitors of resistance pathways, such as mTOR, CDK4/6, or PI3K inhibitors. For fulvestrant, sustained-release nanoparticulate or depot formulations have been explored as potential alternatives to large-volume intramuscular injections, although their clinical value remains to be established. Overall, nanocarrier-based endocrine delivery remains at the preclinical stage.

### 6.3. Delivery of Targeted Therapy and Small Molecule Inhibitors

Small molecule inhibitors of intracellular kinases implicated in oncogenic signaling, such as CDK4/6 inhibitors (palbociclib, ribociclib, abemaciclib), PI3K/AKT/mTOR inhibitors (alpelisib, everolimus), poly(ADP-ribose) polymerase (PARP) inhibitors (olaparib, talazoparib for BRCA1/2-mutant tumors), and tyrosine kinase inhibitors (lapatinib, neratinib for HER2^+^), have transformed the management of metastatic breast cancer, yet present pharmacokinetic challenges of their own that justify nanoparticulate formulation [190]. Variable oral bioavailability, pronounced hepatic first-pass metabolism, heterogeneous tissue distribution, and dose-limiting toxicities, including hematological, hepatic, and gastrointestinal toxicity, constrain administrable doses and necessitate frequent treatment interruptions [191]. Nanoparticles co-delivering CDK4/6 inhibitors with chemotherapeutics, such as palbociclib and doxorubicin in hybrid lipid–polymer systems, generate documented antiproliferative synergism in models of endocrine therapy-resistant HR^+^ breast cancer, through simultaneous G1-phase cell cycle arrest and induction of DNA damage [192]. Nanoparticulate delivery of PARP inhibitors in patients with BRCA1/2 mutations may improve the therapeutic index through preferential concentration of olaparib in tumors already exhibiting DNA repair deficiency, maximizing the synthetic lethality effect and minimizing systemic medullar toxicity [192].

### 6.4. Delivery of Gene Therapy

Gene therapy, based on the delivery of nucleic acid molecules capable of silencing oncogenic genes (via RNA interference), restoring the function of lost tumor suppressor genes, or editing aberrant genomic sequences, represents the most advanced and most promising frontier of molecular oncology applied to breast cancer, with potential to address disease mechanisms inaccessible to conventional pharmacology [193,194]. The principal challenge of therapeutic delivery at the nucleic acid level resides in the intrinsic fragility of RNA and DNA molecules in biological environments, characterized by rapid degradation in the presence of plasma nucleases with a half-life on the order of minutes; a negative charge that impedes traversal of cell membranes; and an incapacity for spontaneous endosomal escape, rendering nanoparticulate carriers an absolute requirement for any clinical application [194].

**Figure 6:**
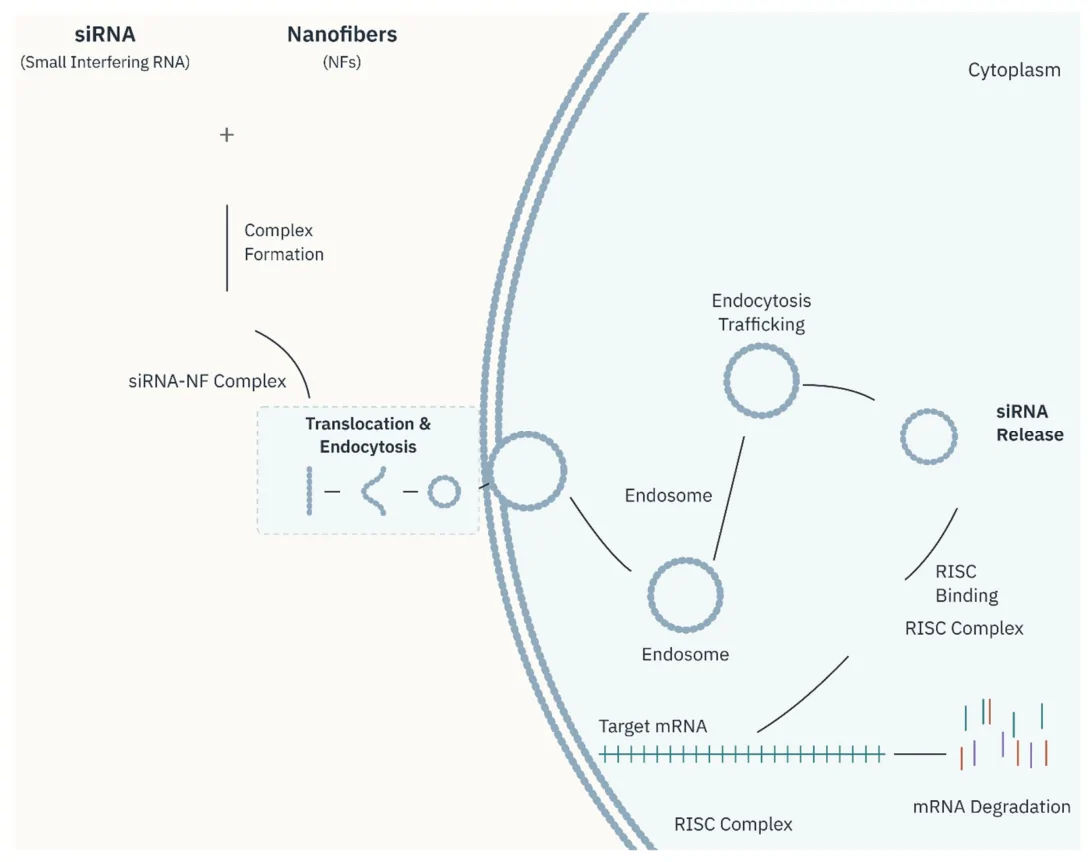
Representation of the hypothesized internalization mechanism of the complex siRNA. Adapted from [174].

Small interfering RNA (siRNA), which are double-stranded RNA molecules of 21–23 nucleotides that mediate sequence-specific degradation of target messenger RNA through the RNA interference (RNAi) mechanism (Figure 6), have demonstrated remarkable preclinical efficacy in silencing oncogenic genes of central importance in breast cancer: VEGF (anti-angiogenic), HER2 (anti-proliferative in the HER2^+^ subtype), Bcl-2 (pro-apoptotic), MDM2 (p53 restoration), ABC efflux pumps (anti-MDR), and genes implicated in the Notch, Wnt, and Hedgehog pathways of BCSCs [195]. Loading of siRNA in ionizable lipid nanoparticles, which is the platform clinically validated in anti-COVID-19 mRNA vaccines, generates transfection efficiencies superior to classical cationic lipoplexes, with a significantly improved toxicity profile. MicroRNA (miRNA), which are endogenous single-stranded RNA molecules of 19–25 nucleotides that post-transcriptionally regulate the expression of multiple target genes simultaneously, offer unique therapeutic versatility in the context of breast cancer. Restoration of miR-34a (a tumor suppressor frequently lost in TNBC), inhibition of oncomiR-21 (overexpressed in 90% of cases and associated with chemotherapy resistance), or restoration of miR-200 (implicated in suppression of epithelial-to-mesenchymal transition) represent strategies with demonstrated clinical potential in preclinical models [196].

Anti-nucleolin aptamers conjugated to nanoparticles co-loaded with anti-Bcl-2 siRNA and doxorubicin have demonstrated synergistic antitumoral efficacy in resistant TNBC models, combining anti-apoptotic silencing, direct cytotoxicity, and cellular recognition specificity [196].

CRISPR/Cas9 (Clustered Regularly Interspaced Short Palindromic Repeats-associated Protein 9), being the precision genome editing system enabling the induction of double-strand breaks at defined DNA sequences with unprecedented specificity in molecular biology, has opened revolutionary perspectives for breast cancer therapy, with the potential for permanent correction of oncogenic mutations, restoration of tumor suppressor genes, and recalibration of DNA repair mechanisms [197]. The components of the CRISPR/Cas9 system, such as the Cas9 protein (or its encoding mRNA), the guide RNA (sgRNA), and, in the case of homology-directed repair editing, the donor DNA, present multiple delivery challenges, including the large dimensions of Cas9 (~160 kDa), the degradability of guide RNA, and the necessity of co-delivering all components to the same intracellular compartment. Dendrimer-type ionizable lipid nanoparticles (dLNPs) and PBAE polymeric nanoparticles have demonstrated genome editing efficiencies of 40–60% in breast cancer cell lines through co-encapsulation of Cas9 mRNA and sgRNA, with facilitated endosomal escape enabled by the ionizable structure of the lipidic components [198]. Specific applications in breast cancer include BRCA2 mutations to induce sensitivity to PARP inhibitors, silencing of the MDR1/ABCB1 gene to re-sensitize cells to chemotherapy, editing of the programmed death-ligand 1 (PD-L1) promoter to reduce tumor immunosuppression, and correction of gain-of-function (GOF) TP53 mutations frequently encountered in TNBC.

### 6.5. Delivery of Proteins and Peptides

Therapeutic proteins, including monoclonal antibodies, enzymes, cytokines, growth factors, and bioactive peptides, constitute a category of agents with exceptional therapeutic potential, yet with severe pharmacokinetic challenges that limit their clinical efficacy in the absence of nanoparticulate carriers [199]. Therapeutic monoclonal antibodies exhibit plasma half-lives on the order of weeks, but extremely limited deep intratumoral penetration due to their large dimensions (~150 kDa), which reduces therapeutic concentration at the level of perivascular tumor cells and favors the emergence of resistance through sublethal pressure mechanisms [178]. Nanobodies (VHH) represent an alternative with superior intratumoral penetration, reduced plasma circulation time (favorable for repeated administrations), and versatile conjugation capacity to nanoparticulate platforms. Therapeutic peptides with pro-apoptotic activity (BH3-mimetics, Smac peptides), anti-angiogenic activity (iRGD peptides, anti-VEGF peptides), or BCSC reprogramming effects benefit from nanoparticulate loading through protection against plasma proteolysis and facilitation of cell membrane penetration, which constitutes the primary barrier to their systemic application [200].

### 6.6. Co-Delivery and Combined Delivery Systems

The simultaneous co-delivery of two or more therapeutic agents with complementary or synergistic mechanisms of action within a single nanoparticulate vehicle represents the most advanced and most promising paradigm of therapeutic cargo delivery in breast cancer, simultaneously addressing multiple pathological mechanisms and reducing the probability of resistance selection [201]. The principal advantages of nanoparticulate co-delivery over combinations of free drugs include maintenance of the optimal molar ratio of the two agents at the tumor site; guaranteed intracellular co-localization of both agents; and the potential for temporal synchronization of their release.

Nanoparticulate co-delivery systems combining chemotherapeutic agents with efflux inhibitors have shown promise for overcoming P-glycoprotein (P-gp)-mediated multidrug resistance (MDR). For example, polymeric nanovesicles and polymer–lipid hybrid nanoparticles co-delivering doxorubicin and elacridar have improved intracellular drug accumulation and antitumour activity in drug-resistant breast cancer models [202,203]. Similarly, the co-delivery of MDR1 siRNA and paclitaxel using solid lipid nanoparticles, or the combination of siMDR1-loaded dendrimer complexes with paclitaxel, has enhanced chemosensitivity through the downregulation of P-gp expression and improved cytotoxic activity [204]. Nanoparticle-mediated chemo-immunotherapy represents another promising strategy, combining immunogenic cell death induced by chemotherapeutic agents with immune checkpoint blockade. Doxorubicin-based prodrug nanoparticles incorporating an anti-PD-L1 peptide have demonstrated concurrent induction of immunogenic cell death and relief of PD-L1-mediated immunosuppression in breast tumour models [205]. Finally, theranostic nanoplatforms incorporating therapeutic agents and imaging components, such as SPIONs or gold nanoparticles, offer opportunities to integrate drug delivery with non-invasive imaging. MRI-visible iron oxide nanocarriers have demonstrated the feasibility of imaging-assisted nucleic acid delivery in multidrug-resistant breast cancer models. Nevertheless, further validation is required before these approaches can reliably guide individualized dosing or treatment regimens [206,207].

## 7. Clinically Approved DDS for Breast Cancer

The clinical translation of nanomedicine and targeted drug-delivery technologies has progressively transformed breast cancer therapy by modifying drug pharmacokinetics, biodistribution, tumor exposure, and toxicity. Among the most clinically established platforms are albumin-bound nanoparticles, liposomal formulations, and antibody–drug conjugates (ADCs), with the latter representing one of the most successful forms of biologically mediated drug delivery [122,208,209]. Unlike conventional chemotherapy, which is associated with substantial nonspecific exposure of healthy tissues, these systems are designed to improve the therapeutic index by altering drug disposition or selectively transporting highly potent payloads to tumor cells.

One of the earliest clinically established nanotechnology-based formulations in breast cancer is Abraxane (nab-paclitaxel), an albumin-bound nanoparticle formulation of paclitaxel. Unlike conventional paclitaxel formulations, nab-paclitaxel does not require Cremophor EL-based solvent systems, thereby eliminating solvent-associated hypersensitivity and permitting administration without routine steroid premedication [122]. In a phase III trial in metastatic breast cancer, nab-paclitaxel demonstrated higher response rates and longer time to progression than conventional solvent-based paclitaxel, supporting its clinical development as an alternative paclitaxel formulation. Abraxane is FDA-approved for metastatic breast cancer after failure of combination chemotherapy or relapse within six months of adjuvant chemotherapy. Thus, nab-paclitaxel illustrates how reformulation of an established cytotoxic drug can modify its pharmacological properties and improve its clinical utility.

Another clinically important delivery strategy is represented by liposomal anthracyclines, particularly pegylated liposomal doxorubicin (PLD; Doxil^®^/Caelyx^®^). Encapsulation of doxorubicin within a PEGylated lipid bilayer prolongs circulation and alters tissue distribution, reducing exposure of some normal tissues to free doxorubicin [119,210]. Importantly, liposomal formulations can substantially reduce anthracycline-associated cardiotoxicity while retaining antitumor activity [119,211]. In the United States, Doxil^®^ is approved for ovarian cancer, AIDS-related Kaposi’s sarcoma, and multiple myeloma, but not for breast cancer; in the European Union, however, the same formulation (Caelyx^®^) is approved as monotherapy for metastatic breast cancer in patients with increased cardiac risk, based on a phase III trial demonstrating comparable efficacy and reduced cardiotoxicity compared with conventional doxorubicin [119]. In addition, non-PEGylated liposomal doxorubicin (Myocet^®^) is approved in the European Union, but not in the United States, for the first-line treatment of metastatic breast cancer in combination with cyclophosphamide [116]. These formulations differ in composition, pharmacokinetics, and approved indications and should therefore not be regarded as interchangeable (Table 1). This distinction is important because the clinical relevance of a DDS should not be equated with the existence of a breast-cancer-specific FDA indication.

While liposomes and nanoparticles primarily modify pharmacokinetics and tissue distribution, ADCs provide a more sophisticated form of targeted drug delivery by combining a tumor-recognizing monoclonal antibody, a cytotoxic payload, and a chemical linker [208,209]. Following antigen binding, ADCs undergo internalization and intracellular processing, releasing the cytotoxic payload within or near the target cell. Depending on the properties of the payload and linker, membrane-permeable drugs may additionally produce a bystander effect, allowing cytotoxic activity against neighboring antigen-low or antigen-negative cells and potentially improving efficacy in heterogeneous tumors.

Among the most established breast cancer ADCs is Kadcyla (ado-trastuzumab emtansine; T-DM1), which combines the HER2-targeting antibody trastuzumab with the microtubule inhibitor DM1. T-DM1 provides selective delivery of a highly potent cytotoxic payload to HER2-expressing cells and has demonstrated substantial clinical benefit in both metastatic and early HER2-positive breast cancer [127,128]. In the KATHERINE trial, adjuvant T-DM1 significantly reduced the risk of invasive disease recurrence or death compared with trastuzumab in patients with residual invasive disease following neoadjuvant therapy, establishing targeted payload delivery as an important component of curative-intent treatment [128].

The next generation of HER2-directed ADCs has further expanded the potential of targeted delivery. Enhertu (fam-trastuzumab deruxtecan-nxki; T-DXd) consists of a HER2-directed antibody linked through a cleavable linker to the topoisomerase I inhibitor deruxtecan. Its high drug-to-antibody ratio of approximately 8 and membrane-permeable payload enable potent cytotoxic activity and a bystander effect, which may be particularly advantageous in tumors with heterogeneous HER2 expression [132,133]. In DESTINY-Breast03, T-DXd significantly improved progression-free survival compared with T-DM1 in previously treated HER2-positive metastatic breast cancer. Importantly, DESTINY-Breast04 subsequently demonstrated significant improvements in progression-free and overall survival in patients with HER2-low metastatic breast cancer, expanding the concept of HER2-directed delivery beyond conventionally HER2-positive disease. Consistent with these findings, the FDA approved T-DXd in January 2025 for unresectable or metastatic HR-positive, HER2-low or HER2-ultralow breast cancer after progression on endocrine therapy [129].

The clinical role of T-DXd has subsequently expanded into earlier and first-line disease settings. In December 2025, the FDA approved T-DXd plus pertuzumab for first-line treatment of unresectable or metastatic HER2-positive breast cancer based on DESTINY-Breast09, in which the combination significantly prolonged progression-free survival compared with taxane, trastuzumab, and pertuzumab [130,134]. In May 2026, the FDA further approved T-DXd in two HER2-positive early breast cancer settings: as part of a sequential neoadjuvant regimen for stage II–III disease and as adjuvant therapy for patients with residual invasive disease following neoadjuvant trastuzumab-based therapy [131]. These developments illustrate the rapid transition of ADCs from predominantly salvage therapies toward biomarker-guided treatment across the continuum of breast cancer care.

Another clinically important targeted delivery platform is Trodelvy (sacituzumab govitecan-hziy), an ADC directed against TROP-2 and conjugated to SN-38, the active metabolite of irinotecan and a potent topoisomerase I inhibitor. TROP-2 is broadly expressed in breast cancer, making it an attractive target for payload delivery [136]. In the phase III ASCENT trial, sacituzumab govitecan significantly improved progression-free and overall survival compared with single-agent chemotherapy in previously treated metastatic TNBC. Its clinical role has subsequently expanded: in June 2026, the FDA approved sacituzumab govitecan as first-line monotherapy for unresectable locally advanced or metastatic TNBC in patients who are not candidates for PD-1/PD-L1 inhibitor therapy and in combination with pembrolizumab for selected PD-L1-positive TNBC [135]. These developments further demonstrate the evolution of ADCs from conventional cytotoxic delivery systems toward biomarker-guided therapeutic platforms.

The development of Datroway (datopotamab deruxtecan-dlnk) further illustrates the rapid expansion of TROP-2-directed delivery. This ADC combines a TROP-2-directed antibody with a topoisomerase I inhibitor payload. It received FDA approval in January 2025 for unresectable or metastatic HR-positive/HER2-negative breast cancer after prior endocrine therapy and chemotherapy, based on the TROPION-Breast01 trial [137,139]. In May 2026, its indication was expanded to unresectable or metastatic TNBC in patients who are not candidates for PD-1/PD-L1 inhibitor therapy, based on TROPION-Breast02 [138]. Thus, TROP-2-directed ADCs are emerging as clinically relevant delivery platforms across multiple breast cancer subtypes rather than being restricted to a single molecular population.

Collectively, these clinically validated systems demonstrate several fundamental advantages of advanced drug delivery. First, they can modify pharmacokinetics and biodistribution, as demonstrated by albumin-bound and liposomal formulations. Second, they can reduce formulation-associated toxicity, as exemplified by solvent-free nab-paclitaxel. Third, they can improve the therapeutic index of highly potent cytotoxic agents by coupling them to tumor-targeting molecules, as demonstrated by ADCs. Fourth, they enable delivery according to tumor-associated molecular characteristics, such as HER2 or TROP-2 expression, thereby integrating drug delivery with precision oncology. Companion diagnostics have consequently become increasingly important; for example, FDA-authorized HER2 assays are used to identify patients eligible for several HER2-directed therapies, including T-DM1 and T-DXd [212]. The regulatory status and key clinical evidence of the main nanocarrier formulations and antibody–drug conjugates discussed in this review are summarized in Table 1.

To clearly distinguish clinically validated platforms from those that remain investigational, the current evidence can be summarized as follows. Clinical benefit in breast cancer has been demonstrated for a limited number of delivery systems: liposomal anthracyclines, which reduce cardiotoxicity while maintaining antitumor efficacy but have not shown superior survival compared with conventional doxorubicin [116,119]; albumin-bound paclitaxel, which improved the response rate and time to progression compared with solvent-based paclitaxel [122]; micellar paclitaxel, which showed non-inferior efficacy in a phase III trial and is approved only in South Korea [164]; and antibody–drug conjugates, which have produced improvements in progression-free and/or overall survival in randomized phase III trials [127,128,132]. In contrast, ligand-targeted nanocarriers have not yet demonstrated clinical benefit, as illustrated by MM-302 [152], and the remaining platforms discussed in this review, including solid lipid nanoparticles, nanostructured lipid carriers, polymeric nanoparticles other than albumin-bound formulations, dendrimers, inorganic and hybrid nanoparticles, biomimetic systems, extracellular vesicles, stimuli-responsive systems, and nanocarrier-based gene delivery, remain predominantly at the preclinical stage in breast cancer (Table 2). Throughout this review, findings obtained in cell culture or animal models should therefore be interpreted as proof-of-concept rather than as evidence of clinical efficacy.

The enhanced permeability and retention (EPR) effect has historically provided an important rationale for nanoparticle-based cancer drug delivery, as abnormal tumor vasculature and impaired lymphatic drainage can promote nanoparticle accumulation within tumors. However, increasing clinical evidence indicates that the EPR effect is highly heterogeneous and considerably less predictable in humans than in experimental tumor models [25]. Variations in vascular permeability, extracellular matrix density, interstitial pressure, tumor perfusion, and immune-cell composition can markedly influence nanoparticle accumulation and penetration. Therefore, EPR alone should not be considered a universal mechanism of clinical tumor targeting. Instead, clinically successful systems increasingly combine favorable pharmacokinetic properties with biologically mediated targeting, controlled payload release, or both. In this context, ADCs are best regarded as molecularly targeted drug-delivery platforms rather than conventional nanocarriers, although they clearly fall within the broader pharmacological concept of targeted DDS.

It is also important to distinguish clinically approved DDS from molecularly targeted breast cancer therapeutics that do not themselves function as drug-delivery systems. CDK4/6 inhibitors, selective estrogen receptor degraders, PARP inhibitors, and PI3K/AKT-pathway inhibitors are examples of precision therapeutics selected according to tumor biology but are not, strictly speaking, DDS. Their distinction from ADCs is conceptually important: whereas conventional targeted drugs act through their own pharmacological properties, ADCs directly integrate molecular recognition, drug transport, and intracellular payload delivery within a single therapeutic entity.

Overall, the clinical development of nab-paclitaxel, liposomal anthracyclines, and particularly HER2- and TROP-2-directed ADCs demonstrates that advanced drug-delivery technologies have progressed from experimental concepts to integral components of modern breast cancer treatment. Their success establishes a central translational principle: therapeutic benefit can be achieved not only by discovering new drugs, but also by controlling where, when, and in which cells potent drugs are delivered. The rapid expansion of T-DXd, sacituzumab govitecan, and datopotamab deruxtecan across increasingly defined molecular populations further indicates that future DDS development will increasingly integrate targeted delivery, biomarker-guided patient selection, intracellular payload release, bystander activity, and companion diagnostics, thereby linking nanomedicine more closely with precision and personalized breast cancer therapy.

## 8. Emerging Trends and Future Directions

The future of breast cancer drug delivery is shifting from conventional carriers designed primarily to improve solubility or circulation toward precision, multifunctional, and adaptive systems integrating nanotechnology, molecular targeting, immunotherapy, nucleic-acid therapeutics, biomimetic materials, and artificial intelligence (AI). Increasing tumor heterogeneity and treatment resistance highlight the need for patient-specific DDSs guided by molecular biomarkers such as HER2, TROP-2, ER, PD-L1, BRCA1/2, and PIK3CA, while AI and machine learning may accelerate formulation optimization and prediction of tumor accumulation, toxicity, and therapeutic response. Biomimetic carriers, particularly extracellular vesicles, and stimuli-responsive systems capable of responding to tumor-associated pH, redox conditions, enzymes, hypoxia, or reactive oxygen species offer opportunities for more selective and controlled drug release, although manufacturing, reproducibility, and regulatory challenges remain. Improving delivery across biological barriers, particularly the blood–brain barrier, is also critical for patients with brain metastases and will increasingly rely on receptor-targeted and BBB-penetrating nanocarriers. Multifunctional DDSs enabling co-delivery of chemotherapeutics, targeted agents, immune modulators, or nucleic acids may help overcome multifactorial resistance while enhancing tumor co-localization and therapeutic synergy. At the same time, ADCs, including T-DM1, trastuzumab deruxtecan, sacituzumab govitecan, and datopotamab deruxtecan, are likely to remain a major driver of innovation through improved targets, linkers, payloads, and strategies to overcome antigen heterogeneity and ADC resistance. Nucleic-acid delivery and theranostic platforms may further enable gene modulation, real-time monitoring, and treatment adaptation, while integration with liquid biopsy and molecular profiling could ultimately support dynamic precision drug delivery that responds to tumor evolution. Despite these advances, successful translation will depend on overcoming tumor heterogeneity, limited penetration, manufacturing complexity, safety, scalability, regulatory barriers, and cost. Ultimately, next-generation DDSs will be defined not by technological sophistication alone but by their ability to deliver reproducible, patient-specific, clinically meaningful improvements in efficacy, survival, quality of life, and treatment safety.

## 9. Conclusions

The next generation of breast cancer drug delivery systems (DDSs) will move from “delivering drugs to tumors” toward “intelligently delivering the right therapeutic payload to the right tumor cell, at the right time, while adapting to tumor heterogeneity and treatment-induced evolution.” This transition reflects a fundamental shift from conventional drug delivery toward precision and adaptive drug delivery, in which the carrier, therapeutic payload, targeting strategy, and release profile are increasingly designed according to the molecular and biological characteristics of individual tumors.

The clinical success of liposomal formulations, albumin-bound nanoparticles, and, most notably, antibody–drug conjugates (ADCs) has already demonstrated the therapeutic value of controlling drug distribution and selectively delivering highly potent agents to cancer cells. The expanding clinical application of HER2- and TROP-2-directed ADCs further illustrates the potential of targeted delivery to improve therapeutic outcomes across multiple breast cancer subtypes and disease stages. Nevertheless, substantial challenges remain, particularly with respect to tumor heterogeneity, treatment resistance, limited penetration into metastatic sites, systemic toxicity, and the variable relationship between nanocarrier accumulation and clinical benefit.

Future DDSs are therefore expected to increasingly integrate molecular targeting, biomarker-guided patient selection, artificial intelligence, bioinspired materials, stimuli-responsive release, nucleic-acid delivery, immunotherapy, and multifunctional combination strategies. Such systems may enable simultaneous targeting of tumor cells and their microenvironment, modulation of resistance mechanisms, and improved delivery to anatomically challenging sites such as the brain. The incorporation of theranostic capabilities and real-time monitoring could further facilitate treatment-response assessment and dynamic adjustment of therapeutic strategies.

At the same time, technological sophistication must be accompanied by clinical translatability. Reproducible large-scale manufacturing, physicochemical and biological characterization, long-term safety, regulatory compliance, cost-effectiveness, and patient-specific variability remain critical barriers to the widespread implementation of advanced DDSs. Future research should therefore move beyond demonstrating enhanced drug loading, cellular uptake, or tumor accumulation in experimental models and focus increasingly on clinically meaningful endpoints, including improved survival, reduced toxicity, prevention of resistance, and enhanced quality of life.

Ultimately, the future of breast cancer drug delivery will depend on the successful convergence of nanotechnology, precision oncology, computational science, molecular diagnostics, and clinical medicine. The most impactful DDSs are unlikely to be those that merely maximize drug accumulation, but rather those capable of integrating targeting, controlled release, biological responsiveness, and adaptability while remaining manufacturable and clinically practical. Such systems have the potential to transform drug delivery from a predominantly passive pharmaceutical technology into an active component of personalized cancer treatment, offering more precise, effective, and safer therapeutic strategies for patients with both early-stage and advanced breast cancer.

## Figures and Tables

**Table 1:** Clinically approved and clinical-stage drug delivery systems relevant to breast cancer, including nanocarrier formulations and antibody–drug conjugates: formulation, regulatory status, and key clinical evidence.

Product	Formulation	Regulatory Status in Breast Cancer	Key Clinical Evidence in Breast Cancer	Status
**Nanocarrier formulations**				
Doxil^®^ (USA)/Caelyx^®^ (EU)	PEGylated liposomal doxorubicin	FDA: no breast cancer indication (approved for ovarian cancer, AIDS-related Kaposi’s sarcoma, multiple myeloma) [117]; EMA: monotherapy for MBC in patients with increased cardiac risk [118]	Phase III vs. conventional doxorubicin in MBC: comparable efficacy, reduced cardiotoxicity [119]	Marketed
Myocet^®^	Non-PEGylated liposomal doxorubicin	EMA (2000): first-line MBC, in combination with cyclophosphamide [120]; not approved by the FDA	Phase III vs. doxorubicin + cyclophosphamide in MBC: preserved efficacy, reduced cardiotoxicity [116]	Marketed (EU)
Abraxane^®^	Albumin-bound paclitaxel nanoparticles	FDA: MBC after failure of combination chemotherapy for metastatic disease or relapse within 6 months of adjuvant chemotherapy; EMA: MBC after failure of first-line treatment when anthracycline-containing therapy is not indicated [121]	Phase III vs. solvent-based paclitaxel in MBC: higher response rate and longer time to progression [122]	Marketed
Genexol-PM^®^	Paclitaxel in PEG-PDLLA polymeric micelles	South Korea: breast, lung, and ovarian cancers [123]; not approved by the FDA	Phase III vs. conventional paclitaxel in MBC: non-inferior efficacy [124]	Marketed (South Korea)
MM-302	HER2-targeted antibody–liposomal doxorubicin conjugate	Investigational; not approved	Non-comparative phase I in advanced HER2-positive breast cancer [125]; randomized phase II HERMIONE (NCT02213744) stopped in 2016 for lack of expected benefit [126]	Discontinued
**Antibody–drug conjugates**				
Kadcyla^®^ (T-DM1)	Anti-HER2 ADC (trastuzumab–DM1)	FDA: HER2-positive MBC; adjuvant treatment of HER2-positive early breast cancer with residual invasive disease	Phase III in HER2-positive advanced breast cancer [127]; phase III KATHERINE in early disease [128]	Marketed
Enhertu^®^ (T-DXd)	Anti-HER2 ADC (trastuzumab–topoisomerase I inhibitor)	FDA: HER2-positive, HER2-low, and HER2-ultralow MBC; first-line HER2-positive disease with pertuzumab; HER2-positive early breast cancer [129,130,131]	DESTINY-Breast03 [132]; DESTINY-Breast04 [133]; DESTINY-Breast09 [134]	Marketed
Trodelvy^®^ (sacituzumab govitecan)	Anti-TROP-2 ADC (SN-38 payload)	FDA: metastatic TNBC, including first-line use in selected patients [135]	Phase III ASCENT in previously treated metastatic TNBC [136]	Marketed
Datroway^®^ (datopotamab deruxtecan)	Anti-TROP-2 ADC (topoisomerase I inhibitor)	FDA: HR-positive/HER2-negative MBC after endocrine therapy and chemotherapy; metastatic TNBC in patients not eligible for PD-1/PD-L1 inhibitors [137,138]	Phase III TROPION-Breast01 [139]	Marketed

ADC, antibody–drug conjugate; EMA, European Medicines Agency; FDA, U.S. Food and Drug Administration; MBC, metastatic breast cancer; PEG-PDLLA, poly(ethylene glycol)–poly(D,L-lactide); TNBC, triple-negative breast cancer.

**Table 2:** Overview of the main drug delivery platforms investigated for breast cancer: composition, size, advantages, limitations, and clinical status.

Platform	Typical Composition	Typical Size	Main Advantages	Main Limitations	Clinical Status in Breast Cancer
**Lipid-based systems**					
Liposomes [115,116]	Phospholipid bilayer(s), cholesterol, ± PEG	~80–200 nm	Biocompatible; hydrophilic and lipophilic cargo; prolonged circulation (PEGylated); reduced cardiotoxicity of anthracyclines	Physical instability; drug leakage; manufacturing cost; no proven overall survival benefit over conventional chemotherapy	Approved (Caelyx^®^, Myocet^®^; EU) (Table 1); ligand-targeted liposomes not approved
Solid lipid nanoparticles (SLNs) [140]	Solid lipid core (triglycerides, waxes, fatty acids) + surfactant	50–1000 nm	Low cost; scalable (high-pressure homogenization); good stability; no organic solvents	Low drug loading; drug expulsion during storage (lipid polymorphism)	Preclinical
Nanostructured lipid carriers (NLCs) [140]	Blend of solid and liquid lipids + surfactant	Similar to SLNs	Higher loading and better storage stability than SLNs; biphasic release	Limited in vivo and clinical data	Preclinical
**Polymeric systems**					
Polymeric nanoparticles (e.g., PLGA) [147]	Biodegradable synthetic (PLGA, PCL) or natural (chitosan) polymers	~50–300 nm	Tunable degradation and sustained release; versatile functionalization	Batch-to-batch variability; scale-up complexity; potential immunogenicity of coatings	Preclinical
Albumin-bound nanoparticles	Human serum albumin + drug	~130 nm	Solvent-free (no Cremophor EL); improved tolerability	Limited to albumin-binding drugs	Approved (nab-paclitaxel) (Table 1)
Polymeric micelles [144]	Amphiphilic block copolymers (e.g., PEG-PDLLA, PEG-PCL)	5–100 nm	Solubilization of hydrophobic drugs; excipient-free taxane formulations; small size	Dissociation upon dilution below the CMC; limited loading	Approved in South Korea (Genexol-PM^®^) (Table 1)
Dendrimers [152,153,154]	Branched polymers (e.g., PAMAM)	~1–14 nm	Monodisperse; high density of surface groups; nucleic acid complexation	Cytotoxicity of cationic higher generations; complex synthesis and cost	Preclinical
**Inorganic and hybrid systems**					
Gold nanoparticles [158]	Au spheres, rods, stars	Shape-dependent	Photothermal conversion (NIR); imaging; versatile surface chemistry	Non-biodegradable; long-term tissue accumulation	Preclinical
Mesoporous silica nanoparticles [159]	Porous SiO_2_	Pore size 2–50 nm	Very high loading capacity; gated or stimulus-responsive release	Biodegradation and long-term safety insufficiently characterized	Preclinical
Superparamagnetic iron oxide nanoparticles [160]	Fe_3_O_4_/γ-Fe_2_O_3_ core ± coating	Core < 20 nm	Magnetic hyperthermia; magnetic guidance; MRI contrast (theranostics)	Heterogeneous heating; tissue accumulation; need for external equipment	Preclinical (therapeutic applications)
Lipid–polymer hybrid nanoparticles [162,163]	Polymeric core + lipid shell	Nanoscale (formulation-dependent)	Combines controlled release with lipid-shell biocompatibility; co-delivery	More complex manufacturing and characterization	Preclinical
**Biomimetic systems**					
Cell membrane-coated nanoparticles [169]	Synthetic core + erythrocyte, leukocyte, or tumor cell membrane	Core-dependent	Immune evasion; homotypic targeting	Membrane source standardization; scale-up; batch variability	Preclinical
Extracellular vesicles/exosomes [111,170]	Cell-derived lipid bilayer vesicles	30–150 nm (exosomes)	Endogenous origin; low immunogenicity; nucleic acid and protein cargo	Low isolation yield; heterogeneity; loading efficiency; standardization	Preclinical

CMC, critical micelle concentration; MRI, magnetic resonance imaging; NIR, near-infrared; PAMAM, polyamidoamine; PCL, polycaprolactone; PEG, poly(ethylene glycol); PEG-PDLLA, poly(ethylene glycol)–poly(D,L-lactide); PLGA, poly(lactic-co-glycolic acid).

## Data Availability

No new data were created or analyzed in this study. Data sharing is not applicable to this article.

## References

[1] Kim J., Harper A., McCormack V., Sung H., Houssami N., Morgan E., Mutebi M., Garvey G., Soerjomataram I., Fidler-Benaoudia M.M. (2025). Global Patterns and Trends in Breast Cancer Incidence and Mortality across 185 Countries. Nat. Med..

[2] Sung H., Ferlay J., Siegel R.L., Laversanne M., Soerjomataram I., Jemal A., Bray F. (2021). Global Cancer Statistics 2020: GLOBOCAN Estimates of Incidence and Mortality Worldwide for 36 Cancers in 185 Countries. CA Cancer J. Clin..

[3] Arnold M., Morgan E., Rumgay H., Mafra A., Singh D., Laversanne M., Vignat J., Gralow J.R., Cardoso F., Siesling S. (2022). Current and Future Burden of Breast Cancer: Global Statistics for 2020 and 2040. Breast.

[4] Heer E., Harper A., Escandor N., Sung H., McCormack V., Fidler-Benaoudia M.M. (2020). Global Burden and Trends in Premenopausal and Postmenopausal Breast Cancer: A Population-Based Study. Lancet Glob. Health.

[5] American Cancer Society Key Statistics for Breast Cancer. https://www.cancer.org/cancer/types/breast-cancer/key-statistics.html.

[6] Giaquinto A.N., Sung H., Newman L.A., Freedman R.A., Smith R.A., Star J., Jemal A., Siegel R.L. (2024). Breast Cancer Statistics 2024. CA Cancer J. Clin..

[7] Prat A., Pineda E., Adamo B., Galván P., Fernández A., Gaba L., Diez M., Viladot M., Arance A., Munoz M. (2015). Clinical Implications of the Intrinsic Molecular Subtypes of Breast Cancer. Breast.

[8] Harbeck N., Penault-Llorca F., Cortes J., Gnant M., Houssami N., Poortmans P., Ruddy K., Tsang J., Cardoso F. (2019). Breast Cancer. Nat. Rev. Dis. Primers.

[9] Foulkes W.D., Smith I.E., Reis-Filho J.S. (2010). Triple-Negative Breast Cancer. N. Engl. J. Med..

[10] Bianchini G., Balko J.M., Mayer I.A., Sanders M.E., Gianni L. (2016). Triple-Negative Breast Cancer: Challenges and Opportunities of a Heterogeneous Disease. Nat. Rev. Clin. Oncol..

[11] Quail D.F., Joyce J.A. (2013). Microenvironmental Regulation of Tumor Progression and Metastasis. Nat. Med..

[12] Binnewies M., Roberts E.W., Kersten K., Chan V., Fearon D.F., Merad M., Coussens L.M., Gabrilovich D.I., Ostrand-Rosenberg S., Hedrick C.C. (2018). Understanding the Tumor Immune Microenvironment (TIME) for Effective Therapy. Nat. Med..

[13] Loibl S., Poortmans P., Morrow M., Denkert C., Curigliano G. (2021). Breast Cancer. Lancet.

[14] Jeselsohn R., Buchwalter G., De Angelis C., Brown M., Schiff R. (2015). ESR1 Mutations—A Mechanism for Acquired Endocrine Resistance in Breast Cancer. Nat. Rev. Clin. Oncol..

[15] Hanker A.B., Sudhan D.R., Arteaga C.L. (2020). Overcoming Endocrine Resistance in Breast Cancer. Cancer Cell.

[16] Luo M., Wicha M.S. (2015). Epithelial-Mesenchymal Plasticity of Breast Cancer Stem Cells: Implications for Metastasis and Therapeutic Resistance. Curr. Pharm. Des..

[17] Kong D., Hughes K., Ford H.L. (2020). Cellular Plasticity in Breast Cancer Progression and Therapy. Front. Oncol..

[18] Vasan N., Baselga J., Hyman D.M. (2019). A View on Drug Resistance in Cancer. Nature.

[19] Armenian S.H., Lacchetti C., Barac A., Carver J., Constine L.S., Denduluri N., Dent S., Douglas P.S., Durand J.B., Ewer M. (2017). Prevention and Monitoring of Cardiac Dysfunction in Survivors of Adult Cancers: ASCO Clinical Practice Guideline. J. Clin. Oncol..

[20] Staff N.P., Grisold A., Grisold W., Windebank A.J. (2017). Chemotherapy-Induced Peripheral Neuropathy: A Current Review. Ann. Neurol..

[21] Burstein H.J. (2020). Systemic Therapy for Estrogen Receptor-Positive, HER2-Negative Breast Cancer. N. Engl. J. Med..

[22] Cardoso F., Spence D., Mertz S., Corneliussen-James D., Sabelko K., Gralow J., Cardoso M.J., Peccatori F., Paonessa D., Benares A. (2018). Global Analysis of Advanced/Metastatic Breast Cancer: Decade Report (2005–2015). Breast.

[23] Jain R.K., Stylianopoulos T. (2010). Delivering Nanomedicine to Solid Tumors. Nat. Rev. Clin. Oncol..

[24] Chauhan V.P., Stylianopoulos T., Boucher Y., Jain R.K. (2011). Delivery of Molecular and Nanoscale Medicine to Tumors: Transport Barriers and Strategies. Annu. Rev. Chem. Biomol. Eng..

[25] Sun R., Xiang J., Zhou Q., Piao Y., Tang J., Shao S., Zhou Z., Bae Y.H., Shen Y. (2022). The Tumor EPR Effect for Cancer Drug Delivery: Current Status, Limitations, and Alternatives. Adv. Drug Deliv. Rev..

[26] Mitchell M.J., Billingsley M.M., Haley R.M., Wechsler M.E., Peppas N.A., Langer R. (2021). Engineering Precision Nanoparticles for Drug Delivery. Nat. Rev. Drug Discov..

[27] Bae Y.H., Park K. (2011). Targeted Drug Delivery to Tumors: Myths, Reality and Possibility. J. Control Release.

[28] Anselmo A.C., Mitragotri S. (2016). Nanoparticles in the Clinic. Bioeng. Transl. Med..

[29] Kimta N., Majdalawieh M.A., Majdalawieh A.F. (2026). The Future of Breast Cancer Treatment: From Conventional Wisdom to Nanotechnology-Enabled Precision. Int. J. Mol. Sci..

[30] Danhier F., Feron O., Préat V. (2010). To Exploit the Tumor Microenvironment: Passive and Active Tumor Targeting of Nanomedicines. J. Control Release.

[31] Mura S., Nicolas J., Couvreur P. (2013). Stimuli-Responsive Nanocarriers for Drug Delivery. Nat. Mater..

[32] Karimi M., Ghasemi A., Zangabad P.S., Rahighi R., Basri S.M.M., Mirshekari H., Amiri M., Pishabad Z.S., Aslani A., Bozorgomid M. (2016). Smart Micro/Nanoparticles in Stimulus-Responsive Drug/Gene Delivery Systems. Chem. Soc. Rev..

[33] Hare J.I., Lammers T., Ashford M.B., Puri S., Storm G., Barry S.T. (2017). Challenges and Strategies in Anti-Cancer Nanomedicine Development: An Industry Perspective. Adv. Drug Deliv. Rev..

[34] Agrahari V., Agrahari V. (2018). Facilitating the Translation of Nanomedicines to a Clinical Product: Challenges and Opportunities. Drug Discov. Today.

[35] Araújo J.L., Vieira J.A., Silva M.D.S., Lima A.K.O., Luz G.V.d.S., Carneiro M.L.B., Azevedo R.B. (2023). Benefits of Using Polymeric Nanoparticles in Breast Cancer Treatment: A Systematic Review. 3 Biotech.

[36] Choi S.H., Ramesh S., Reed S., Menyah G., Ganschow P., Henderson V., Dunnenberger H.M. (2026). Breaking Barriers to Completing Genetic Testing for Inherited Breast Cancer among At-Risk Black Women Using a Community-Based Participatory Research Approach. Hum. Genom. Adv..

[37] Kong X., Cheng R., Zhang W., Lu Y., Kan Y., Fang Y., Gao J., Wang J., Hwang K.C. (2026). Nanoparticle-Based Immunotherapeutic Strategies to Overcome Cancer Drug Resistance: From Biological Barriers to Artificial Intelligence-Driven Design. Drug Resist. Updat..

[38] Ramos-Castaneda J.A., Joven-Gonzalez D.A., Cardozo-Vasquez O.Y., Perez-Becerra V. (2026). Health System Barriers to Early Detection and Timely Treatment of Breast Cancer: A Scoping Review. J. Cancer Policy.

[39] Antonini M., Santos G.M., Mattar A., Teixeira M.D., Amorim A.G., de Figueiredo M.F., do Nascimento Moreira Ramos M., Cavalcante F.P., Millen E.C., Frasson A.L. (2025). Barriers to Breast Cancer Treatment in Brazil: A Study on Migration and Regional Disparities. Public Health Pract..

[40] Allison K.H., Hammond M.E.H., Dowsett M., McKernin S.E., Carey L.A., Fitzgibbons P.L., Hayes D.F., Lakhani S.R., Chavez-MacGregor M., Perlmutter J. (2020). Estrogen and Progesterone Receptor Testing in Breast Cancer: ASCO/CAP Guideline Update. J. Clin. Oncol..

[41] Wolff A.C., Hammond M.E.H., Allison K.H., Harvey B.E., Mangu P.B., Bartlett J.M.S., Bilous M., Ellis I.O., Fitzgibbons P., Hanna W. (2018). Human Epidermal Growth Factor Receptor 2 Testing in Breast Cancer: American Society of Clinical Oncology/College of American Pathologists Clinical Practice Guideline Focused Update. J. Clin. Oncol..

[42] Zhao J., Zhou Z., Saw P.E., Song E. (2025). Silver Jubilee of HER2 Targeting: A Clinical Success in Breast Cancer. J. Natl. Cancer Cent..

[43] Murrell D.H., Hamilton A.M., Mallett C.L., van Gorkum R., Chambers A.F., Foster P.J. (2015). Understanding Heterogeneity and Permeability of Brain Metastases in Murine Models of HER2-Positive Breast Cancer through Magnetic Resonance Imaging: Implications for Detection and Therapy. Transl. Oncol..

[44] Lehmann B.D., Bauer J.A., Chen X., Sanders M.E., Chakravarthy A.B., Shyr Y., Pietenpol J.A. (2011). Identification of Human Triple-Negative Breast Cancer Subtypes and Preclinical Models for Selection of Targeted Therapies. J. Clin. Investig..

[45] (2012). Cancer Genome Atlas Network. Comprehensive Molecular Portraits of Human Breast Tumours. Nature.

[46] Sabit H., Adel A., Abdelfattah M.M., Ramadan R.M., Nazih M., Abdel-Ghany S., El-Hashash A., Arneth B. (2025). The Role of Tumor Microenvironment and Immune Cell Crosstalk in Triple-Negative Breast Cancer (TNBC): Emerging Therapeutic Opportunities. Cancer Lett..

[47] Liu B., Zhou G., Su F. (2025). Breast Cancer Tumor Immune Microenvironment Landscape Identifies Prognostic Risk Genes and Potential Drugs. Sci. Rep..

[48] Rocha-Vieira T.C., Meyer-Fernandes J.R. (2025). Ectonucleotidases: Possible Roles in the Tumor Microenvironment and Influence on Tumor Progression in Breast Cancer. Biochim. Biophys. Acta Gen. Subj..

[49] Dai Y., Pan X., Guo S., Ji S., Cao S., Montierth M.D., Jiang Y., Chang J.T., Shi L., Shalapour S. (2026). Tumor Microenvironment Transcriptional Activity Enables Robust Stratification of Chemotherapy Response in Triple-Negative Breast Cancer. Cell Rep. Med..

[50] Wang C., Xu J., Zhang Y., Nie G. (2023). Emerging Nanotechnological Approaches to Regulating Tumor Vasculature for Cancer Therapy. J. Control Release.

[51] Lunca S., Morarasu S., Dimofte G.M. (2026). Nanoparticle-Mediated Radiosensitization in Breast Cancer: A Systematic Review of Preclinical Evidence and Translational Challenges. Int. J. Mol. Sci..

[52] Fu X., Zhang H., Liu W., Wu T., Zheng Y. (2026). Hypoxia-responsive self-assembled nanoparticles for reprogramming tumor-associated macrophages into an anti-tumor M1 phenotype in breast cancer. Mater. Today Bio.

[53] Wang Z., Hasan I., Zhang Y., Peng T., Guo B. (2025). Photothermal Combination Therapy for Metastatic Breast Cancer: A New Strategy and Future Perspectives. Biomedicines.

[54] Wilhelm S., Tavares A.J., Dai Q., Ohta S., Audet J., Dvorak H.F., Chan W.C.W. (2016). Analysis of Nanoparticle Delivery to Tumours. Nat. Rev. Mater..

[55] Esmaeilizadeh N., Maleki-Kakelar H., Sharifi R., Valilou S., Jalili A., Asgharzadeh M.R. (2024). Effects of Different Glucose Concentrations and Hypoxia and Its Impact on the Expression of Sox2 and CAIX in Breast Cancer: In Vitro and In Silico Analysis. Biomed. Pharmacother..

[56] Qi Y., Lv S., Xie C., Du S., Yao J. (2025). Dual-Phase Nanoscissors Disrupt Vasculature-Breast Cancer Stem Cell Crosstalk for Breast Cancer Treatment. J. Control Release.

[57] Li P., Dong Z., Jiang Q., Yu C., Liu Y., Yuan R., Qiu Y., Meng T., Hu F., Yuan H. (2026). Sunitinib Lipid Nanoparticles Remodel Tumor Vasculature to Synergize with Two-Stage Radiotherapy for Enhanced Radiosensitivity in Breast Cancer. J. Control Release.

[58] Qi X., Lian Y., Fan Z., Wang H., Jiang H., He M., Li L., Huang J., Wan Y. (2025). Electroacupuncture Normalized Tumor Vasculature by Downregulating Glyoxalase-1 to Polarize Tumor-Associated Macrophage to M1 Phenotype in Triple-Negative Breast Cancer. Int. Immunopharmacol..

[59] Malarz K., Borzęcka W., Ziola P., Domiński A., Rawicka P., Bialik-Wąs K., Kurcok P., Torres T., Mrozek-Wilczkiewicz A. (2025). pH-Sensitive Phthalocyanine-Loaded Polymeric Nanoparticles as a Novel Treatment Strategy for Breast Cancer. Bioorg. Chem..

[60] Yu T.Y., Zhang G., Chai X.X., Ren L., Yin D.C., Zhang C.Y. (2023). Recent Progress on the Effect of Extracellular Matrix on Occurrence and Progression of Breast Cancer. Life Sci..

[61] Lin M., Jiang Y., Chen Y., Yang H., Xun Y. (2026). Multidimensional Regulatory Mechanisms of Extracellular Matrix Stiffness in Breast Cancer and Its Prospects for Clinical Translation. Clin. Breast Cancer.

[62] Chitty J.L., Cox T.R. (2025). The Extracellular Matrix in Cancer: From Understanding to Targeting. Trends Cancer.

[63] Safaei S., Heydari S., Dehghan Manshadi M., Ashtari B., Gholipourmalekabadi M., Sasani F., Hashemi F., Vosoogh M., Madjd Z., Ghods R. (2025). Tumor Matrix Stiffness Drives Malignant Progression in Murine Breast Cancer: Enhanced Stemness, Tumorigenesis and Metastasis. Mater. Adv..

[64] Tam N.W., Dimova R. (2024). Breast Cancer Cell-Derived Extracellular Vesicles Accelerate Collagen Fibrillogenesis and Integrate into the Matrix. bioRxiv.

[65] Srivastava A., Mishra S., Avadhesh, Shekher A., Rai V., Dhasmana A., Das J., Perenzoni D., Iori R., Gupta S.C. (2024). Moringin, an Isothiocyanate Modulates Multiple Cellular Signalling Molecules in Breast Cancer Cells. Cell Signal..

[66] Coullomb A., Foulon C., van Haastrecht B., Monsarrat P., Pancaldi V. (2026). Mosna Reveals Different Types of Cellular Interactions Predictive of Response to Immunotherapies and Survival in Cancer. Mol. Cell Proteom..

[67] Rahimi S., Chen Y., Zareian M., Pandit S., Mijakovic I. (2022). Cellular and Subcellular Interactions of Graphene-Based Materials with Cancerous and Non-Cancerous Cells. Adv. Drug Deliv. Rev..

[68] Lopes D., Lopes J., Serpico L., Santos H.A., Paiva-Santos A.C. (2026). Harnessing Nature’s Blueprint: Unlocking the Potential of Subcellular Structure Membrane-Coated Nanosystems for Precision Medicine. Bioact. Mater..

[69] Dang W., Cui T., Jiao G., Li J., Liu Z. (2025). Subcellular Organelle-Mediated Precise Delivery of Nanomedicines: Advances and Applications in Cancer Therapy. J. Drug Deliv. Sci. Technol..

[70] Avolio E., Bassani B., Campanile M., Mohammed K.A.K., Muti P., Bruno A., Spinetti G., Madeddu P. (2025). Shared Molecular, Cellular, and Environmental Hallmarks in Cardiovascular Disease and Cancer: Any Place for Drug Repurposing?. Pharmacol. Rev..

[71] Chen H., Ke J., Wang R., Li Y., Luo K., Zhang L., Song S., Jia S., Luo M., Liu D. (2026). Ticagrelor Reverses Multidrug Resistance in Breast Cancer by Inhibiting PI3K/AKT/mTOR Pathway and Suppressing ABCB1 Expression and Function. Biochem. Pharmacol..

[72] Mao J., Qiu L., Ge L., Zhou J., Ji Q., Yang Y., Long M., Wang D., Teng L., Chen J. (2021). Overcoming Multidrug Resistance by Intracellular Drug Release and Inhibiting P-Glycoprotein Efflux in Breast Cancer. Biomed. Pharmacother..

[73] Ativui S., Danquah C.A., Osafo N., Adu W., Ofori M. (2021). Palmatine Sensitizes Chemoresistant Triple Negative Breast Cancer Cells via Efflux Inhibition of Multidrug Resistant Protein 1. Sci. Afr..

[74] Bharathiraja P., Yadav P., Sajid A., Ambudkar S.V., Prasad N.R. (2023). Natural Medicinal Compounds Target Signal Transduction Pathways to Overcome ABC Drug Efflux Transporter-Mediated Multidrug Resistance in Cancer. Drug Resist. Updat..

[75] Deb Nath S., Akash M.M.H., Tanim M.T.H., Islam M.M., Rahman M.S., Rahman M.H., Islam M.R., Hossain M.S. (2025). Expression and Interaction of RNA Binding Protein HuR with Multidrug Transporters (P-GP, MRP1, and ABCG2) in Chemoresistant Breast and Colorectal Cancer Cells. J. Genet. Eng. Biotechnol..

[76] Nie Y., Wei Y., Zhang Y., Liang Z., Lei Z., Chang M., Peng Y. (2025). Design and Implication of a Breast Cancer-Targeted Drug Delivery System Utilizing the Kisspeptin/GPR54 System. Int. J. Pharm..

[77] Jayaraj S., Jayaprakash N., Devaraji M., Murugavel S., Robinson A.X., Murugesan L. (2026). Nanocarrier-Based Strategies for Advanced Cancer Drug Delivery and Therapeutics: Design Principles, Biological Interactions, and Clinical Potential. Nano Trends.

[78] Liu Y., Xie Y., Nie X., Yang W., Fu C. (2026). Research Progress on Metal-Organic Framework-Based Drug Delivery Systems for Breast Cancer Treatment. Colloid Interface Sci. Commun..

[79] Bishnoi A., Bonde G.V. (2025). Beyond Nanomicelles and Hydrogels: Exploring Micelle-Containing Hydrogels as Cutting-Edge Drug Delivery System for Effective Breast Cancer Treatment. Int. J. Pharm..

[80] Wang T., Chen Y., Zhang X., Dou Y., Chen Y., Xie M., Motta A., Zheng Z., Wang X., Kuang X. (2026). Nanotechnology-Driven Drug Delivery Systems for Breast Cancer: A Review. Biomater. Adv..

[81] Qi K., Zhang S., Bi X., Zhang Z., Yang C. (2026). Tuning Mechanical Softness as a Design Principle in Drug Delivery: A Biomechanical Perspective. Acta Pharm. Sin. B.

[82] Price L.S.L., Stern S.T., Deal A.M., Kabanov A.V., Zamboni W.C. (2020). A Reanalysis of Nanoparticle Tumor Delivery Using Classical Pharmacokinetic Metrics. Sci. Adv..

[83] Kirpotin D.B., Drummond D.C., Shao Y., Shalaby M.R., Hong K., Nielsen U.B., Marks J.D., Benz C.C., Park J.W. (2006). Antibody Targeting of Long-Circulating Lipidic Nanoparticles Does Not Increase Tumor Localization but Does Increase Internalization in Animal Models. Cancer Res..

[84] Park J.W., Hong K., Kirpotin D.B., Colbern G., Shalaby R., Baselga J., Shao Y., Nielsen U.B., Marks J.D., Moore D. (2002). Anti-HER2 Immunoliposomes: Enhanced Efficacy Attributable to Targeted Delivery. Clin. Cancer Res..

[85] Roscigno G., Jacobs S., Toledo B., Borea R., Russo G., Pepe F., Serrano M.J., Calabrò V., Troncone G., Giovannoni R. (2025). The Potential Application of Stroma Modulation in Targeting Tumor Cells: Focus on Pancreatic Cancer and Breast Cancer Models. Semin. Cancer Biol..

[86] Pérez-Herrero E., Fernández-Medarde A., Irache J.M. (2026). Albumin-Based Nanocarriers: Singularities, Synthesis Methods, Clinical Relevance and Targeting Strategies in Cancer. Acta Pharm. Sin. B.

[87] Spreen H., Barth C., Keuter L., Mulac D., Humpf H.-U., Langer K. (2022). Tuning the Protein Corona of PLGA Nanoparticles: Characterization of Trastuzumab Adsorption Behavior and Its Cellular Interaction with Breast Cancer Cell Lines. J. Drug Deliv. Sci. Technol..

[88] Chantada-Vázquez M.P., Castro López A., García Vence M., Vázquez-Estévez S., Acea-Nebril B., Calatayud D.G., Jardiel T., Bravo S.B., Núñez C. (2020). Proteomic Investigation on Bio-Corona of Au, Ag and Fe Nanoparticles for the Discovery of Triple Negative Breast Cancer Serum Protein Biomarkers. J. Proteom..

[89] Tok K., Ghorbanizamani F., Moulahoum H., Barlas F.B., Celik E.G., Gürsoy D., Memmedov R., Akcam T.I., Turhan K., Zihnioglu F. (2025). Corona-Like Nanostructures: Quantum Dots Meet Pleural Fluid Proteins/Peptides for Theranostic Applications. Colloids Surf. B Biointerfaces.

[90] Lu Y., Sun Y., Zhang D., He Z., Sun B., Sun J. (2025). Customization of Protein Corona to Reprogram Cancer Nanomedicines: Mechanistic Insights and Therapeutic Implications. J. Control Release.

[91] Mu X., Li Y., Wang S., Fu H., Gao M., Lu Y., Zhou X. (2025). A Dynamic Protein Corona Substitution Strategy for Activatable Imaging-Guided Synergistic Theranostics in Triple-Negative Breast Cancer. Mater. Today Bio.

[92] Keivany M.R., Shojaei Asrami E. (2026). Engineering Stimuli-Responsive Prodrugs to Overcome Barriers in Cancer Immunotherapy. Int. J. Pharm..

[93] Wu M., Sheng J., Xie Q., Qi Y., Zhao Y., Zhang S. (2025). Recent Advances in Stimuli-Responsive Hyaluronic Acid-Based Nanodelivery Systems for Cancer Treatment: A Review. Int. J. Biol. Macromol..

[94] Mousavi S.M., Yari Kalashgrani M., Yang C.-J., Neralla V.R., Lai J.-Y. (2026). Stimuli-Responsive, Prodrug-Activating Enzyme-Functionalized Metal-Organic Frameworks for Cancer Therapy and Tumor Microenvironment Modulation. Coord. Chem. Rev..

[95] Jha A.K., Arora S., Sahu R.C., Kumar D., Singh S., Agrawal A.K. (2025). A Review on Integrated Design and Application of Stimuli-Responsive ZIF-8 Metal-Organic Frameworks For breast cancer theranostics. Next Mater..

[96] Yilmaz A.S., Pepe A., Latifi N., Sawyer G.W., Dunne M.R. (2026). Stimuli-Responsive Hydrogels in Targeted Cancer Therapy: A Systematic Review of Materials, Mechanisms, and Delivery Strategies. Mater. Des..

[97] Wang J., Zhang T., Liao Y., Chen W., Liang S., Xu S., Fang H., Wang M., Zheng L., Gu Z. (2023). 3D-Imaging and Quantitative Assessment for Size-Related Penetration of HfO_2_ Nanoparticles in Breast Cancer Tumor by Synchrotron Radiation Microcomputed Tomography. Anal. Chim. Acta.

[98] Liu J., Xiao S., Huang C., Zhang X., Cai R., Qu S., Peng Y., Xie L. (2025). Emodin Nanoparticles-Loaded Injectable Hydrogel with Deep Tumor Penetration for Triple-Negative Breast Cancer Therapy. Biomater. Adv..

[99] Ekinci M., Koksal-Karayildirim C., Ilem-Ozdemir D. (2022). Radiolabeled Methotrexate Loaded Chitosan Nanoparticles as Imaging Probe for Breast Cancer: Biodistribution in Tumor-Bearing Mice. J. Drug Deliv. Sci. Technol..

[100] Kshirsagar R., Bule P., Kandula J.S., Kundu S., Pullapanthula R., Sahu B.D., Chella N. (2026). Unveiling the Potential of Telmisartan in Breast Cancer Using Nanocrystal Approach: Formulation, Optimization, In Vitro, and In Vivo Pharmacokinetic Evaluation. J. Drug Deliv. Sci. Technol..

[101] Zhang Z., Zhang Y., Gao R., Zhao C., Zhao M., Huang Y. (2026). Pharmacokinetic Profiling of the Bioactive Components of Sijunzi Decoction in a Rat Model of Breast Cancer: Insights into Herb-Drug Interactions with Cyclophosphamide. J. Pharm. Biomed. Anal..

[102] Yao X., Xiong F., Chen H., Cao X., Chen Q., He J., Zhang L., Zhang W., Fan W., Li X. (2025). GGT-Targeting Polysarcosine-Decorated CaCO_3_ Nanoplatform for ICD-Enhanced Breast Cancer Immunotherapy. Chem. Eng. J..

[103] Li S., Wang Q., Su C., Jia Z., Shen G., Da M., Yang R., Zhao J., Chen D. (2025). GGT and GSH-Triggered Nanoplatform for Efficient Gambogic Acid Delivery and Tumor Penetration in Triple-Negative Breast Cancer. Mater. Today Bio.

[104] Wang L., Deng Y., Zhou X., Song K., Jiang C., Zhao F., Li X. (2026). A Biodegradable Nanomedicine for Potentiated Cancer Therapy via Ca^2+^-Overload and Photothermal Dual-Amplifying Cuproptosis. J. Colloid Interface Sci..

[105] Huang Q., Feng N., Cao X., Xiao J., Li Q., Zhou B., Bai Y., Zhao Y., Zhang Z., Liu Y. (2026). Nano-Immunomodulators Co-Activate Immunogenic Cell Death and the cGAS-STING Pathway for Breast Cancer Immunotherapy. J. Control Release.

[106] Venugopal J., Panneerselvam S., Patan A., Theivendren P. (2026). Artificial Intelligence in Personalized Breast Cancer Drug Safety: From Preclinical Toxicology to Clinical Risk Management. Clin. Ther..

[107] Wang C., Sun J., Song J., Li S., Li Y. (2026). Functional Nanoplatforms Overcoming Immune Resistance in Skin Cancers: Targeted Immunomodulation, Immunogenic Cell Death, and Metabolic Remodeling. Cancer Lett..

[108] Zhang Z., Cao Q., Xia Y., Cui C., Qi Y., Zhang Q., Wu Y., Liu J., Liu W. (2024). Combination of Biodegradable Hydrogel and Antioxidant Bioadhesive for Treatment of Breast Cancer Recurrence and Radiation Skin Injury. Bioact. Mater..

[109] Gandhi N., Modi S., Soni S., Andey T. (2025). Modular Self-Emulsifying Drug Delivery Platform to Enhance Cellular Uptake Activity in Triple-Negative Breast Cancer. Eur. J. Pharm. Sci..

[110] Zhan L., Yin X., Qiu L., Li C., Wang Y. (2024). Application of Dual Chemotherapeutic Drug Delivery System Based on Metal-Organic Framework Platform in Enhancing Tumor Regression for Breast Cancer Research. Biochem. Biophys. Res. Commun..

[111] Gharavi A.T., Irian S., Niknejad A., Parang K., Salimi M. (2024). Harnessing Exosomes as a Platform for Drug Delivery in Breast Cancer: A Systematic Review for In Vivo and In Vitro Studies. Mol. Ther. Oncolytics.

[112] Huang X., Ali A., Yachioui D.E.I., Le Dévédec S.E., Hankemeier T. (2025). Lipid Dysregulation in Triple Negative Breast Cancer: Insights from Mass Spectrometry-Based Approaches. Prog. Lipid Res..

[113] Behravan N., Zahedipour F., Jaafari M.R., Johnston T.P., Sahebkar A. (2022). Lipid-Based Nanoparticulate Delivery Systems for HER2-Positive Breast Cancer Immunotherapy. Life Sci..

[114] Subburaj V., Kuppuswamy U., Veintraimuthu S., Saran, Velmurugan M. (2026). Development and Characterization of Lipid-Based Solid Dispersion of Quassinoid-Enriched Fraction of *Simarouba glauca* for Enhanced Lipid Membrane Permeability in MCF7 Breast Cancer Cells. Next Nanotechnol..

[115] Barenholz Y. (2012). Doxil^®^—The First FDA-Approved Nano-Drug: Lessons Learned. J. Control Release.

[116] Batist G., Ramakrishnan G., Rao C.S., Chandrasekharan A., Gutheil J., Guthrie T., Shah P., Khojasteh A., Nair M.K., Hoelzer K. (2001). Reduced Cardiotoxicity and Preserved Antitumor Efficacy of Liposome-Encapsulated Doxorubicin and Cyclophosphamide Compared with Conventional Doxorubicin and Cyclophosphamide in a Randomized, Multicenter Trial of Metastatic Breast Cancer. J. Clin. Oncol..

[117] U.S. Food and Drug Administration DOXIL (Doxorubicin Hydrochloride Liposome Injection): Prescribing Information. https://dailymed.nlm.nih.gov/dailymed/drugInfo.cfm?setid=917eddf9-1d3f-4abe-8c81-d33b066e5bae.

[118] European Medicines Agency Caelyx Pegylated Liposomal: EPAR. https://www.ema.europa.eu/en/medicines/human/EPAR/caelyx-pegylated-liposomal.

[119] O’Brien M.E.R., Wigler N., Inbar M., Rosso R., Grischke E., Santoro A., Catane R., Kieback D.G., Tomczak P., Orlandi F. (2004). Reduced Cardiotoxicity and Comparable Efficacy in a Phase III Trial of Pegylated Liposomal Doxorubicin HCl versus Conventional Doxorubicin for First-Line Treatment of Metastatic Breast Cancer. Ann. Oncol..

[120] European Medicines Agency Myocet Liposomal (Previously Myocet): EPAR. https://www.ema.europa.eu/en/medicines/human/EPAR/myocet-liposomal-previously-myocet.

[121] European Medicines Agency Abraxane: EPAR. https://www.ema.europa.eu/en/medicines/human/EPAR/abraxane.

[122] Gradishar W.J., Tjulandin S., Davidson N., Shaw H., Desai N., Bhar P., Hawkins M., O’Shaughnessy J. (2005). Phase III Trial of Nanoparticle Albumin-Bound Paclitaxel Compared with Polyethylated Castor Oil-Based Paclitaxel in Women with Breast Cancer. J. Clin. Oncol..

[123] Wang J., Li S., Han Y., Guan J., Chung S., Wang C., Li D. (2018). Poly(Ethylene Glycol)–Polylactide Micelles for Cancer Therapy. Front. Pharmacol..

[124] Park I.H., Sohn J.H., Kim S.B., Lee K.S., Chung J.S., Lee S.H., Kim T.Y., Jung K.H., Cho E.K., Kim Y.S. (2017). An Open-Label, Randomized, Parallel, Phase III Trial Evaluating the Efficacy and Safety of Polymeric Micelle-Formulated Paclitaxel Compared to Conventional Cremophor EL-Based Paclitaxel for Recurrent or Metastatic HER2-Negative Breast Cancer. Cancer Res. Treat..

[125] Munster P., Krop I.E., LoRusso P., Ma C., Siegel B.A., Shields A.F., Molnár I., Wickham T.J., Reynolds J., Campbell K. (2018). Safety and Pharmacokinetics of MM-302, a HER2-Targeted Antibody–Liposomal Doxorubicin Conjugate, in Patients with Advanced HER2-Positive Breast Cancer: A Phase 1 Dose-Escalation Study. Br. J. Cancer.

[126] Merrimack Pharmaceuticals (2016). Merrimack Stops the Phase 2 HERMIONE Trial of MM-302 in HER2-Positive Metastatic Breast Cancer Patients. Press Release. https://www.prnewswire.com/news-releases/merrimack-stops-the-phase-2-hermione-trial-of-mm-302-in-her2-positive-metastatic-breast-cancer-patients-300382177.html.

[127] Verma S., Miles D., Gianni L., Krop I.E., Welslau M., Baselga J., Pegram M., Oh D.-Y., Diéras V., Guardino E. (2012). Trastuzumab Emtansine for HER2-Positive Advanced Breast Cancer. N. Engl. J. Med..

[128] von Minckwitz G., Huang C.-S., Mano M.S., Loibl S., Mamounas E.P., Untch M., Wolmark N., Rastogi P., Schneeweiss A., Redondo A. (2019). Trastuzumab Emtansine for Residual Invasive HER2-Positive Breast Cancer. N. Engl. J. Med..

[129] U.S. Food and Drug Administration FDA Approves Fam-Trastuzumab Deruxtecan-nxki for Unresectable or Metastatic HR-Positive, HER2-Low or HER2-Ultralow Breast Cancer. https://www.fda.gov/drugs/resources-information-approved-drugs/fda-approves-fam-trastuzumab-deruxtecan-nxki-unresectable-or-metastatic-hr-positive-her2-low-or-her2.

[130] U.S. Food and Drug Administration FDA Approves Fam-Trastuzumab Deruxtecan-nxki with Pertuzumab for Unresectable or Metastatic HER2-Positive Breast Cancer. https://www.fda.gov/drugs/drug-approvals-and-databases/fda-approves-fam-trastuzumab-deruxtecan-nxki-pertuzumab-unresectable-or-metastatic-her2-positive.

[131] U.S. Food and Drug Administration FDA Approves Two Separate Indications for Fam-Trastuzumab Deruxtecan-nxki in HER2-Positive Early-Stage Breast Cancer. https://www.fda.gov/drugs/resources-information-approved-drugs/fda-approves-two-separate-indications-fam-trastuzumab-deruxtecan-nxki-her2-positive-early-stage.

[132] Cortés J., Kim S.-B., Chung W.-P., Im S.-A., Park Y.H., Hegg R., Kim M.H., Tseng L.-M., Petry V., Chung C.-F. (2022). Trastuzumab Deruxtecan versus Trastuzumab Emtansine for Breast Cancer. N. Engl. J. Med..

[133] Modi S., Jacot W., Yamashita T., Sohn J., Vidal M., Tokunaga E., Tsurutani J., Ueno N.T., Prat A., Chae Y.S. (2022). Trastuzumab Deruxtecan in Previously Treated HER2-Low Advanced Breast Cancer. N. Engl. J. Med..

[134] Tolaney S.M., Jiang Z., Zhang Q., Barroso-Sousa R., Park Y.H., Rimawi M.F., Saura C., Schneeweiss A., Toi M., Chae Y.S. (2026). Trastuzumab Deruxtecan Plus Pertuzumab for HER2-Positive Metastatic Breast Cancer. N. Engl. J. Med..

[135] U.S. Food and Drug Administration FDA Approves Sacituzumab Govitecan-hziy as Monotherapy and in Combination with Pembrolizumab for First-Line Treatment of Triple-Negative Breast Cancer. https://www.fda.gov/drugs/resources-information-approved-drugs/fda-approves-sacituzumab-govitecan-hziy-monotherapy-and-combination-pembrolizumab-first-line.

[136] Bardia A., Hurvitz S.A., Tolaney S.M., Loirat D., Punie K., Oliveira M., Brufsky A., Sardesai S.D., Kalinsky K., Zelnak A.B. (2021). Sacituzumab Govitecan in Metastatic Triple-Negative Breast Cancer. N. Engl. J. Med..

[137] U.S. Food and Drug Administration FDA Approves Datopotamab Deruxtecan-dlnk for Unresectable or Metastatic HR-Positive, HER2-Negative Breast Cancer. https://www.fda.gov/drugs/resources-information-approved-drugs/fda-approves-datopotamab-deruxtecan-dlnk-unresectable-or-metastatic-hr-positive-her2-negative-breast.

[138] U.S. Food and Drug Administration FDA Approves Datopotamab Deruxtecan-dlnk for Unresectable or Metastatic Triple-Negative Breast Cancer. https://www.fda.gov/drugs/resources-information-approved-drugs/fda-approves-datopotamab-deruxtecan-dlnk-unresectable-or-metastatic-triple-negative-breast-cancer.

[139] Pistilli B., Jhaveri K., Im S.-A., Pernas S., De Laurentiis M., Wang S., Martínez Jañez N., Borges G., Cescon D.W., Hattori M. (2026). Datopotamab Deruxtecan versus Chemotherapy in Previously Treated Inoperable/Metastatic Hormone Receptor-Positive, HER2-Negative Breast Cancer: Final Overall Survival Analysis of the Phase III TROPION-Breast01 Study. Ann. Oncol..

[140] Thakur A., Sharma R., Devi A. (2026). Solid Lipid Nanoparticles for Targeted Drug Delivery in Breast Cancer: Biointerface Interactions, Therapeutic Potential, and Translational Challenges. Pathol. Res. Pract..

[141] Barbosa M.V., Monteiro L.O.F., Carneiro G., Malagutti A.R., Vilela J.M.C., Andrade M.S., Oliveira M.C., Carvalho-Junior A.D., Leite E.A. (2015). Experimental Design of a Liposomal Lipid System: A Potential Strategy for Paclitaxel-Based Breast Cancer Treatment. Colloids Surf. B Biointerfaces.

[142] Fredericq L., Hamouda A.E.I., Zhong Z., Van Ginderachter J.A., De Geest B.G., Laoui D. (2026). Lipid-Based Delivery Systems for Dendritic Cell Targeting: Formulation Strategies and Cancer Applications. J. Control Release.

[143] Wang T., Cao Y., Xiong J., Li L. (2025). Construction of a Nano-Fluorescent Polymer Drug Delivery System for Breast Cancer Treatment. J. Mol. Struct..

[144] Chaudhuri A., Ramesh K., Kumar D.N., Dehari D., Singh S., Kumar D., Agrawal A.K. (2022). Polymeric Micelles: A Novel Drug Delivery System for the Treatment of Breast Cancer. J. Drug Deliv. Sci. Technol..

[145] Gupta U., Sonone P.R., Sharma A., Mungalasetty S., Yadav P., Bharath M., Bhandari V., Chourasia M.K., Singh P.K. (2026). Human Serum Albumin-Based Polymeric Nanoparticles for Ratiometric Co-Encapsulation and Co-Delivery of Palbociclib and Tamoxifen Citrate for Synergistic Effect in Breast Cancer Management. Int. J. Biol. Macromol..

[146] Hammouda S., Husien S., El Salakawy N., Mamdouh W. (2026). Influence of Polymer Hydrophilicity/Hydrophobicity and Drug Loading Strategy (Free vs. Polycaprolactone/Chitosan Nanoparticle-Encapsulated) on Tamoxifen-Loaded Polyvinyl Alcohol and Polycaprolactone Nanofibers for Breast Cancer Therapy. J. Drug Deliv. Sci. Technol..

[147] Kumar V., Jha S.K., Gupta G., Sahebkar A., Kesharwani P. (2025). Unveiling the Power of Polymeric Nanoparticles: Pioneering Advances in Breast Cancer Treatment. Adv. Colloid Interface Sci..

[148] Hughes C.S., Tayeb S., Muir D., Hayes A.J., Watson P., Jones A.T. (2026). Rapid Plasma Membrane Reorganisation and Endocytosis in HER2 Breast Cancer Cells Incubated with Trastuzumab Decorated Polymer Nanoparticles. RSC Pharm..

[149] Chen S., He X., Ye Y., Zhu S., Tian Z., Yang M., Pang S., Li M., Guo R., He Q. (2026). Arachidonic Acid-Functionalized Micelle Induces Ferroptosis of Tumor Cells to Overcome Chemotherapy Resistance for Breast Cancer Therapy. Int. J. Pharm..

[150] Ma M., Bo S., Xu X., Ye K., Bai L., Zhang D., Chen J., Xiao Z., Luo L., Shi C. (2026). A Multi-Modal Imaging Hyaluronic Acid Micelle for Enhanced Mild Photothermal Therapy of Triple-Negative Breast Cancer. Biomater. Adv..

[151] Castillejos M.C., Zuliani A., Cova C.M., Caro C., Lopez Noriega L., Comaills V., García-Martín M.L., Gauthier B.R., Khiar N. (2025). Assessing Small-Sized D-Fructose-Functionalized Micelles for Active Targeting of Triple-Negative Breast Cancer. J. Drug Deliv. Sci. Technol..

[152] Barajas-Mendoza I., Espinosa-Hernández A., Martínez-Klimova E., Ramírez-Ápan T., Martínez-García M. (2026). Bis-MPA Janus Dendrimers-Prednisone Conjugates against Human Breast Cancer Cells. Steroids.

[153] Ramadan D.R., Osman H.A., Madhy S.A., Teleb M., Darwish A.I., Abu-Serie M.M., Haiba N.S., Khattab S.N., Khalil H.H. (2025). A Tailored 4G s-Triazine-Based Dendrimer Vehicle for Quercetin Endowed with MMP-2/9 Inhibition and VEGF Downregulation for Targeting Breast Cancer Progression and Liver Metastasis. RSC Adv..

[154] Dabagh S., Ghorbanpoor H., Soykan M.N., Sarıboyacı A.E., Adinolfi B., Giannetti A., Li Z., Lan N., Guan B.O., Avci H. (2026). Multifunctional Dendrimer Nanocarrier Loaded with Ibuprofen for Synergistic Personalized Theranostics and Targeted Ablation in Breast Cancer. Mater. Today Nano.

[155] Manatunga D.C., de Silva R.M., de Silva K.M.N., Malavige G.N., Wijeratne D.T., Williams G.R., Jayasinghe C.D., Udagama P.V. (2018). Effective Delivery of Hydrophobic Drugs to Breast and Liver Cancer Cells Using a Hybrid Inorganic Nanocarrier: A Detailed Investigation Using Cytotoxicity Assays, Fluorescence Imaging and Flow Cytometry. Eur. J. Pharm. Biopharm..

[156] Rajput P., Khanchandani S. (2026). Chitosan-Functionalized Cu-Doped NiO/Reduced Graphene Oxide Nanocarrier for pH-Triggered Delivery of 5-Fluorouracil in Breast Cancer Cells. Int. J. Biol. Macromol..

[157] Sun Z., Martin T.A., Jiang W.G. (2026). Exploring Nonviral Nanocarriers as RNA Delivery Platforms for Breast Cancer Treatment. Chem. Eng. J..

[158] Mal S., Chakraborty S., Mahapatra M., Pakeeraiah K., Das S., Paidesetty S.K., Roy P. (2024). Tackling Breast Cancer with Gold Nanoparticles: Twinning Synthesis and Particle Engineering with Efficacy. Nanoscale Adv..

[159] Payamifar S., Khalili Y., Foroozandeh A., Abdouss M., Hasanzadeh M. (2025). Magnetic Mesoporous Silica Nanoparticles as Advanced Polymeric Scaffolds for Efficient Cancer Chemotherapy: Recent Progress and Challenges. RSC Adv..

[160] Garg P., Krishna M., Horne D., Salgia R., Singhal S.S. (2025). Hyperthermia-Induced Apoptosis: The Role of Magnetic Nanoparticles in Targeted Delivery for Breast Cancer Treatment. Biochim. Biophys. Acta Rev. Cancer.

[161] Herbsleb L., Wild D., Gröger H., Schubert T., Steyer A.M., Hennies J., Alves F., Feldmann C., Walter A. (2025). 3D Correlative Light and Electron Microscopy Reveals the Uptake and Processing of Inorganic-Organic Hybrid Nanoparticles into Cancer Cells. Nanomed. Nanotechnol. Biol. Med..

[162] Garg N.K., Tyagi R.K., Sharma G., Jain A., Singh B., Jain S., Katare O.P. (2017). Functionalized Lipid–Polymer Hybrid Nanoparticles Mediated Codelivery of Methotrexate and Aceclofenac: A Synergistic Effect in Breast Cancer with Improved Pharmacokinetics Attributes. Mol. Pharm..

[163] Zheng M., Yue C., Ma Y., Gong P., Zhao P., Zheng C., Sheng Z., Zhang P., Wang Z., Cai L. (2013). Single-Step Assembly of DOX/ICG Loaded Lipid–Polymer Nanoparticles for Highly Effective Chemo-Photothermal Combination Therapy. ACS Nano.

[164] Xu X., Song Y., Li M., Liu F., Zhang H., Xu J., Gao J., Lv Y., Zhang B., Garg S. (2025). Biomimetic Tumor Cell Membrane-Camouflaged Nanomicelles for Synergistic Chemo-Immunotherapy of Triple-Negative Breast Cancer. Mater. Today Bio.

[165] Gorbacheva V.I., Grabovoy A.S., Marukhina P.S., Syrocheva A.O., Kolesova E.P. (2026). Biomimetic Nanoparticles in Cancer Photodynamic Therapy: A Review of Targeted Delivery Systems and Therapeutic Outcomes. Beilstein J. Nanotechnol..

[166] Gu X., Gao Y., Wang P., Wang L., Peng H., He Y., Liu Y., Feng N. (2021). Nano-Delivery Systems Focused on Tumor Microenvironment Regulation and Biomimetic Strategies for Treatment of Breast Cancer Metastasis. J. Control Release.

[167] Ai H., Liu Y., Luo X., Zhang W., Liu X., Xiao S., Jin B., Liu J. (2025). Membrane-Coated BLM Gene Silencing Plasmids and Doxorubicin Nanoparticles Targeted for Synergistic Inhibition of Triple-Negative Breast Cancer. Biomed. Pharmacother..

[168] Zhang P., Li C., Gao H., Hu K. (2025). Biomimetic Hybrid Membrane-Coated Nanoparticles Loaded with Rhein-Iron Complex Enhance Ferroptosis and Immunotherapy for Triple-Negative Breast Cancer. Mater. Today Bio.

[169] Rout B., Agnihotri T.G., Jain A. (2024). Advancement in Triple-Negative Breast Cancer Therapeutics: A Comprehensive Review on the Potential of Cell Membrane-Coated Nanoparticles. J. Drug Deliv. Sci. Technol..

[170] Nakase I., Takatani-Nakase T. (2022). Exosomes: Breast Cancer-Derived Extracellular Vesicles; Recent Key Findings and Technologies in Disease Progression, Diagnostics, and Cancer Targeting. Drug Metab. Pharmacokinet..

[171] Zhang H., Li X., Pang H., Ma F., Hu A., Li Y., Cheng W., Chen J., Wang R., Guo B. (2025). CAR-T Exosomes-Platelet Hybrid Vesicles Dual Targeting against Breast Cancer Recurrence and Metastasis. Chem. Eng. J..

[172] Jahangiri L. (2025). The Impact of Extracellular Vesicles on Breast Cancer Metastasis and Therapeutics: Genetic Considerations. Cancer Genet..

[173] Mussa A., Hamid M., Talib M., Ehinnou I., Bragoli A., Hajissa K., Mokhtar N.F., Mohamud R., Al-Hatamleh M.A.I., Hassan R. (2026). Novel Targeted Therapies and Immunological Strategies for Breast Cancer Treatment. Biochem. Pharmacol..

[174] Ghosh R., Kumar M., Kumar S., Komal K., Sharma R., Kurmi B.D. (2025). Small Molecule Therapeutics for Receptor-Mediated Targeting through Liposomes in Breast Cancer Treatment: A Comprehensive Review. Bioorg. Chem..

[175] Prossnitz E.R., Barton M. (2011). The G-Protein-Coupled Estrogen Receptor GPER in Health and Disease. Nat. Rev. Endocrinol..

[176] Khan H.U., Raza N., Maheen S., Abbas H., Salem M.E., Khan I., Naseem M., Shafqat S.S. (2025). Advances in Targeted Drug Delivery Systems for Cancer Treatment: Current Trends and Future Prospects. J. Drug Deliv. Sci. Technol..

[177] Mashayekhi K., Rahnama M., Rahman M.S., Prasad A., Islam S.M. (2026). Targeted Biologics for TNBC: Advances in Nanobodies, Antibodies, Peptides, and Aptamers. Mol. Ther. Oncol..

[178] Wang C., Hu J., Huang Y., Chen Y., Zhan L., Liu H., Yao D., Wang D. (2026). FAP-targeting peptide-directed nanoprobes enable tumor microenvironment-activatable MR/NIRF imaging of breast cancer primary tumor and lung metastases. Mater. Today Bio.

[179] Zhang J., Chen Z. (2026). Integration of TWAS with Single-Cell and Spatial Transcriptomics Identifies TLR1 as a Susceptibility Gene and Therapeutic Target in the Breast Cancer Tumor Microenvironment. Int. J. Biol. Macromol..

[180] Yin C., Chen C., Chen Z., Song Z., Li F. (2026). Targeting Cancer Stem Cells: Dual-Drug Delivery Systems and Their Role in Cancer Therapy. Int. J. Pharm..

[181] Ting J.Z.S., Lim J., Cho T.Y.Y., Yeoh Z.M., Yeo W.W.Y., Lim H.Y. (2026). Therapeutic Potential of MicroRNAs in Targeting Breast Cancer Stem Cells: A Systematic Review. Crit. Rev. Oncol. Hematol..

[182] Baumann Z., Billy E., Scheidmann M.C. (2026). Advancements in Organoid Models Emulating Metastatic Niches. Trends Cancer.

[183] Liu Y., Yang F., Jin Y., Zhang Q., Zhao B., Liang Y., Hou N., Li Q. (2026). Dual Immunometabolic Regulation and Therapeutic Targeting of TAMs, Microglia and Astrocytes in Brain Metastasis of Non-Small Cell Lung Cancer. Biochim. Biophys. Acta Rev. Cancer.

[184] Khan I., Gril B., Paranjape A.N., Robinson C.M., Difilippantonio S., Biernat W., Bieńkowski M., Pęksa R., Duchnowska R., Jassem J. (2023). Comparison of Three Transcytotic Pathways for Distribution to Brain Metastases of Breast Cancer. Mol. Cancer Ther..

[185] Korram P., Sahu P., Satapathy T. (2026). Exosome Inspired Nanocarriers for Molecular Targeted Cancer Therapy: Controlled Cargo Loading, Release Pathways and Pharmacological Translation. J. Drug Deliv. Sci. Technol..

[186] Gupta P., Marwaha D., Sharma S., Sethi V.A. (2025). Hypothesizing the Co-Delivery of Raloxifene and Genistein by Bovine Milk Exosomes for Enhanced Drug Delivery in Breast Cancer Management. Med. Hypotheses.

[187] Goetz M.P., Sangkuhl K., Guchelaar H.-J., Schawab M., Province M., Whirl-Carrillo M., Symmans W.F., McLeod H.L., Ratain M.J., Zembutsu H. (2018). Clinical Pharmacogenetics Implementation Consortium (CPIC) Guideline for CYP2D6 and Tamoxifen Therapy. Clin. Pharmacol. Ther..

[188] Sioufi A., Gauducheau N., Pineau V., Marfil F., Jaouen A., Cardot J.M., Godbillon J., Czendlik C., Howard H., Pfister C. (1997). Absolute Bioavailability of Letrozole in Healthy Postmenopausal Women. Biopharm. Drug Dispos..

[189] Robertson J.F.R., Harrison M. (2004). Fulvestrant: Pharmacokinetics and Pharmacology. Br. J. Cancer.

[190] Kamali M.J., Aram C., Saeedi F., Khoshghiafeh A., Mir M.A., Aram C., Ahmadifard M. (2025). Therapeutic Targeting of Triple-Negative Breast Cancer: A Multi-Model Evaluation of LNA-Anti-miR-19b-3p and Small Molecule Inhibitors. Comput. Biol. Med..

[191] Wu C.-S., Chen H.-F., Huynh K.-T., Huang W.-C., Li Y.-C., Chang W.-C., Chang L.C., Jiang C.L., Huang K.C.Y., Liu L.C. (2026). Targeting PCK1 to Overcome CDK4/6 Inhibitor Resistance for Breast Cancer Therapy. Cancer Lett..

[192] Ge A., Niu L., Rubino R., Seaman K., Song X., Hao Y., Klupt K., Iaboni N., Jia Z., You L. (2025). Alexidine Is a TAZ-Specific Small-Molecule Inhibitor That Suppresses Breast Cancer Invasion and Metastasis. iScience.

[193] Hashemi H., Tayyebi R., Malek S., Jaafari M.R., Malaekeh-Nikouei B., Mohammadi M. (2025). Advanced Localized Gene Transfection and Drug Delivery Using an Injectable In Situ Forming Dextran-Based Hydrogel against Breast Cancer. Int. J. Pharm..

[194] Ragheb Y., Yahia S., Aboelmagd M., El Sherif R.M., El-Sherbiny I.M. (2026). Folic Acid-Conjugated Chitosan/Sodium Hyaluronate-Based Hybrid Nanocarriers as a Dual-Targeted Delivery System for Efficient Breast Cancer Therapy. Int. J. Biol. Macromol..

[195] Javed A., Ur-Rehman U., Yuan R., Chen X., Guo H., Fu Z. (2026). In Vivo Genetic Circuit Drives Systemic PARP1 siRNA Assembly into Small Extracellular Vesicles for BRCA2-Deficient Breast Cancer Therapy. J. Control Release.

[196] Bellavita R., Braccia S., Piccolo M., Bialecki P., Ferraro M.G., Graziano S.F., Esposito E., Donadio F., Bryszewska M., Irace C. (2025). Shielding siRNA by Peptide-Based Nanofibers: An Efficient Approach for Turning off EGFR Gene in Breast Cancer. Int. J. Biol. Macromol..

[197] Paranthaman S., Uthaiah C.A., Md S., Alkreathy H.M. (2025). Comprehensive Strategies for Constructing Efficient CRISPR/Cas Based Cancer Therapy: Target Gene Selection, sgRNA Optimization, Delivery Methods and Evaluation. Adv. Colloid Interface Sci..

[198] Farbiak L., Cheng Q., Wei T., Álvarez-Benedicto E., Johnson L.T., Lee S., Siegwart D.J. (2021). All-In-One Dendrimer-Based Lipid Nanoparticles Enable Precise HDR-Mediated Gene Editing In Vivo. Adv. Mater..

[199] Araki Y., Takatani-Nakase T., Ninomiya S., Matsumoto M., Hirose H., Kawaguchi Y., Fujiwara D., Michigami M., Katoh H., Wada T. (2026). Optimum Serum Concentration Enhances Migration of MDA-MB-231 Triple-Negative Breast Cancer Cells and Promotes Intracellular Delivery of Proapoptotic Domain via Cell-Penetrating Peptides. Mol. Pharm..

[200] Shahmoradipour P., Emamzadeh R. (2026). Advances in the Use of MT1-MMP-Targeted Nanobodies and Peptides for Therapeutic Delivery in Triple-Negative Breast Cancer. Biomed. Pharmacother..

[201] Yin L., Ye J., Du Z., Pan S., You R., Guan Y.-Q. (2026). Epigallocatechin Gallate-Modified Nanoliposome for Co-Delivery of Quercetin and 5-Aminolevulinic Acid Enhances the Inhibitory Efficiency of Growth and Metastasis in Breast Cancer Cells. J. Drug Deliv. Sci. Technol..

[202] Wong H.L., Bendayan R., Rauth A.M., Wu X.Y. (2006). Simultaneous delivery of doxorubicin and GG918 (Elacridar) by new polymer-lipid hybrid nanoparticles (PLN) for enhanced treatment of multidrug-resistant breast cancer. J. Control Release.

[203] Xiao H., He J., Li X., Li B., Zhang L., Wang Y., Cheng D., Shuai X. (2018). Polymeric nanovesicles as simultaneous delivery platforms with doxorubicin conjugation and elacridar encapsulation for enhanced treatment of multidrug-resistant breast cancer. J. Mater. Chem. B.

[204] Li J., Liu J., Guo N., Zhang X. (2016). Reversal of multidrug resistance in breast cancer MCF-7/ADR cells by h-R3-siMDR1-PAMAM complexes. Int. J. Pharm..

[205] Moon Y., Shim M.K., Choi J., Yang S., Kim J., Yun W.S., Cho H., Park J.Y., Kim Y., Seong J.K. (2022). Anti-PD-L1 peptide-conjugated prodrug nanoparticles for targeted cancer immunotherapy combining PD-L1 blockade with immunogenic cell death. Theranostics.

[206] Gautier J., Allard-Vannier E., Munnier E., Soucé M., Chourpa I. (2013). Recent advances in theranostic nanocarriers of doxorubicin based on iron oxide and gold nanoparticles. J. Control Release.

[207] Lin G., Zhu W., Yang L., Wu J., Lin B., Xu Y., Cheng Z., Xia C., Gong Q., Song B. (2014). Delivery of siRNA by MRI-visible nanovehicles to overcome drug resistance in MCF-7/ADR human breast cancer cells. Biomaterials.

[208] Valle I., Grinda T., Antonuzzo L., Pistilli B. (2025). Antibody-Drug Conjugates in Breast Cancer: Mechanisms of Resistance and Future Therapeutic Perspectives. npj Breast Cancer.

[209] Cerci B., Saatci O., Basik M., Sahin O. (2026). Mechanisms of Resistance to Antibody-Drug Conjugates in Breast Cancer. Drug Resist. Updat..

[210] Gabizon A.A. (2001). Pegylated Liposomal Doxorubicin: Metamorphosis of an Old Drug into a New Form of Chemotherapy. Clin. Cancer Res..

[211] van Dalen E.C., van der Pal H.J., Kremer L.C. (2016). Different Dosage Schedules for Reducing Cardiotoxicity in People with Cancer Receiving Anthracycline Chemotherapy. Cochrane Database Syst. Rev..

[212] U.S. Food and Drug Administration List of FDA-Authorized Companion Diagnostic Devices. https://www.fda.gov/medical-devices/in-vitro-diagnostics/list-fda-authorized-companion-diagnostic-devices-in-vitro-and-imaging-tools.

